# Author Correction: Fibroblast A20 governs fibrosis susceptibility and its repression by DREAM promotes fibrosis in multiple organs

**DOI:** 10.1038/s41467-023-36285-7

**Published:** 2023-01-31

**Authors:** Wenxia Wang, Swarna Bale, Jun Wei, Bharath Yalavarthi, Dibyendu Bhattacharyya, Jing Jing Yan, Hiam Abdala-Valencia, Dan Xu, Hanshi Sun, Roberta G. Marangoni, Erica Herzog, Sergejs Berdnikovs, Stephen D. Miller, Amr H. Sawalha, Pei-Suen Tsou, Kentaro Awaji, Takashi Yamashita, Shinichi Sato, Yoshihide Asano, Chinnaswamy Tiruppathi, Anjana Yeldandi, Bettina C. Schock, Swati Bhattacharyya, John Varga

**Affiliations:** 1 Northwestern Scleroderma Program, Department of Medicine, Feinberg School of Medicine, Chicago, IL USA; 2 Michigan Scleroderma Program, Department of Internal Medicine, University of Michigan, Ann Arbor, MI 48109 USA; 3 Division of Pulmonary and Critical Care Medicine, Department of Medicine, Feinberg School of Medicine, Northwestern University, Chicago, IL 60611 USA; 4 Department of Microbiology-Immunology, Northwestern University Feinberg School of Medicine, Chicago, IL 60611 USA; 5 Department of Medicine, Yale University School of Medicine, New Haven, CT USA; 6 Division of Allergy and Immunology, Department of Medicine, Northwestern University Feinberg School of Medicine, Chicago, IL 60611 USA; 7 Department of Medicine, University of Pittsburgh School of Medicine, Pittsburgh, PA USA; 8 Department of Dermatology, University of Tokyo Graduate School of Medicine, Bunkyo-ku, Tokyo, 113-8655 Japan; 9 Department of Pharmacology and Center for Lung and Vascular Biology, College of Medicine, University of Illinois, Chicago, IL USA; 10 Department of Pathology, Feinberg School of Medicine, Northwestern University, Chicago, IL 60611 USA; 11 Wellcome-Wolfson Institute for Experimental Medicine, Queens University Belfast, Belfast, UK

**Keywords:** Autoimmunity, Systemic sclerosis

Correction to: *Nature Communications* 10.1038/s41467-022-33767-y, published online 26 October 2022

The original version of this Article contained an error in Fig. 7F, in which the Western blot images for Smad 2 in the right column (DREAM KO) were a duplication the Smad 2 Western blot in the left column (wt). This has been corrected in both the PDF and HTML versions of the Article.

