# Supplementary material for: Fibroblast A20 governs fibrosis susceptibility and its repression by DREAM promotes fibrosis in multiple organs
Source: Nat Commun. 2022 Oct 26;13:6358. doi: 10.1038/s41467-022-33767-y (PMC9606375; doi:10.1038/s41467-022-33767-y)
Supplement: Supplementary file 1 — Supplementary Information [file 41467_2022_33767_MOESM1_ESM.pdf]

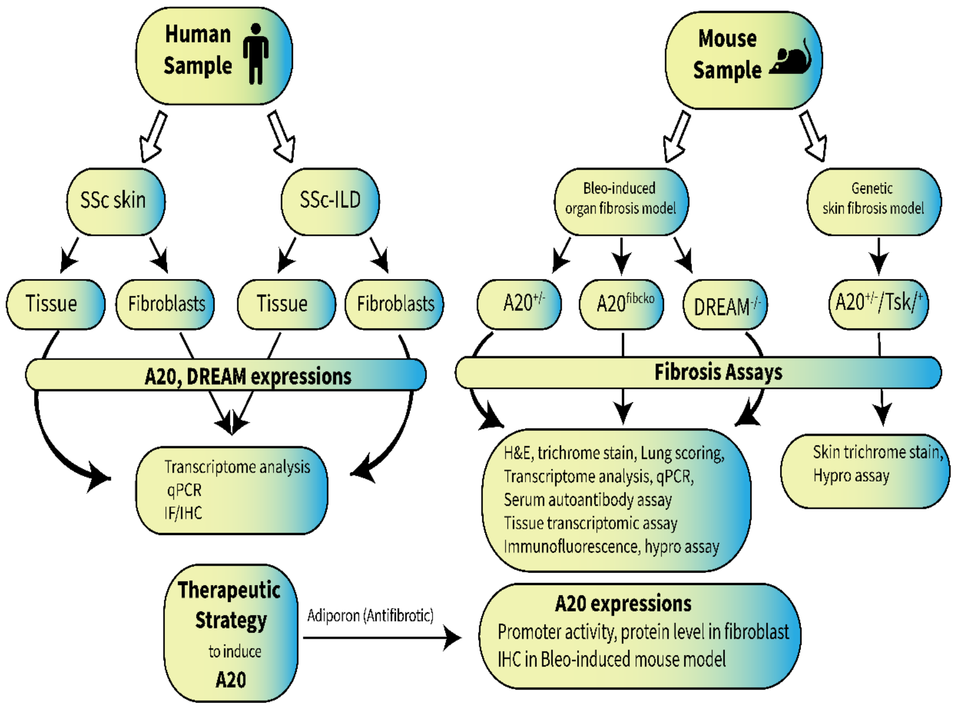

Supplementary Figure 1. Schematic overview of experimental workflow. The experimental approaches using human disease samples and mouse models in this project are illustrated. The graphic was created using University-licensed Adobe Illustrator v.26.3.1.

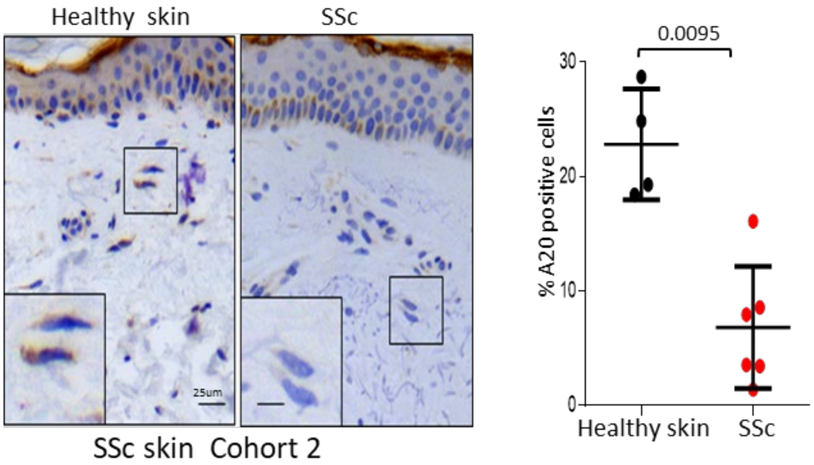

SUPPLEMENTARY FIGURE 2: REDUCED A20 IN AN INDEPENDENT SSC COHORT. Skin biopsies from SSc patients (n=6) and healthy controls (n=4) were immunolabelled with antibodies to A20. Left panels, representative images. Bar=25 µm. Right panel, A20-positive fibroblasts in the dermis were quantitated from four randomly selected fields/hpf. Two-tailed Mann-Whitney *U* test.

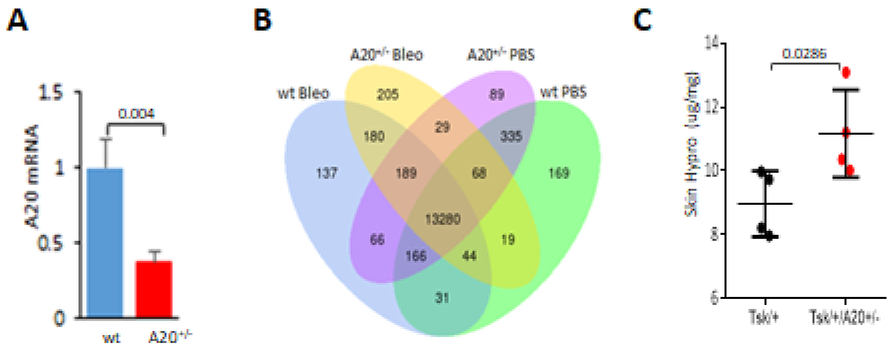

SUPPLEMENTARY FIG. 3. A20 EXPRESSION AND GENOME WIDE TRANSCRIPTOME IN A20$^{+/-}$ MICE AND COLLAGEN CONTENT IN *A20$^{+/-}$; TSK$^{1/+}$* and *TSK$^{1/+}$* MICE. A. A20 mRNA expression in A20$^{+/-}$ mice compared to A20$^{fl/fl}$ mice (n=3). Two tailed Student's t-test. **B.** Venn diagram of genome-wide transcript changes in the skin from PBS and bleo-treated wt (A20$^{fl/fl}$) and A20$^{+/-}$ mice. **C.** Collagen content in the skin of *A20$^{+/-}$; TSK$^{1/+}$* and *TSK$^{1/+}$* mice. Results represent the means ± s.d. from four mice/group**.** Two-tailed Mann-Whitney *U* test.

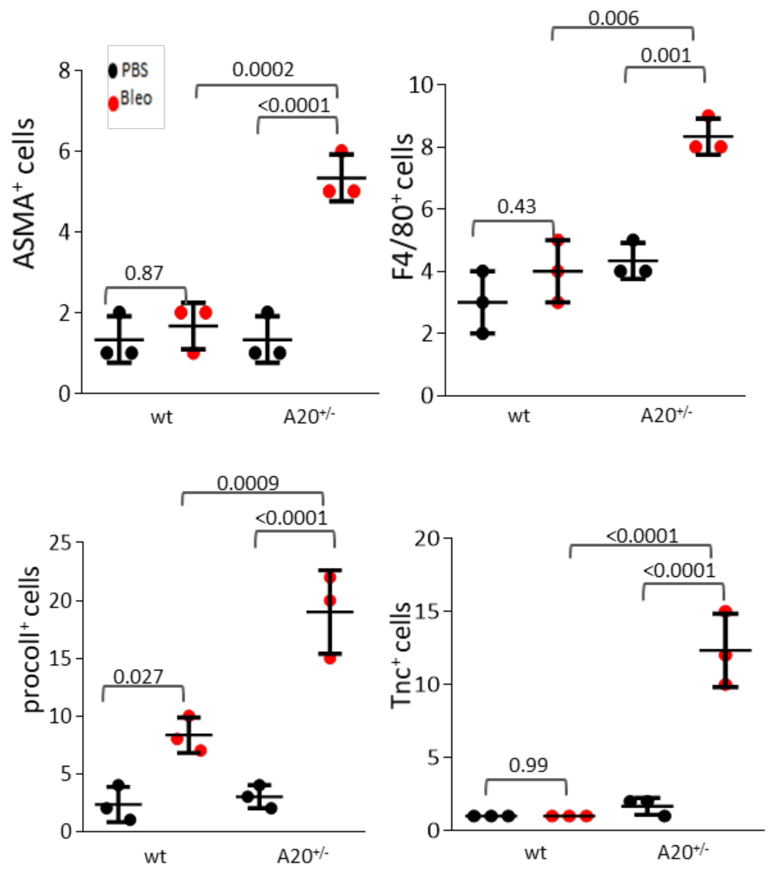

SUPPLEMENTARY FIG. 4. QUANTITATION OF CELLULAR COMPOSITION AND TENASCIN-C ACCUMULATION IN THE LUNGS.

A20$^{fl/fl}$ mice and A20$^{+/-}$ mice in parallel were treated with s.c. bleomycin (10 mg/kg) or PBS for two weeks, and lungs were harvested at day 22 for immunolabelling. Immunofluorescence was performed for the expression of ASMA/F4/80, Procollagen 1 and tenascin-C. Number of immunopositive cells (Fig. 3D) determined from four randomly selected locations/hpf. When quantifying myofibroblasts, ASMA+ cells with distinct vessel morphology were excluded from analysis and only cells with characteristic fibroblast spindle shape is counted. Scale bars=10µm. Results are means ± s.d. from three mice/group. One-way ANOVA followed by Sidak's multiple comparison test.

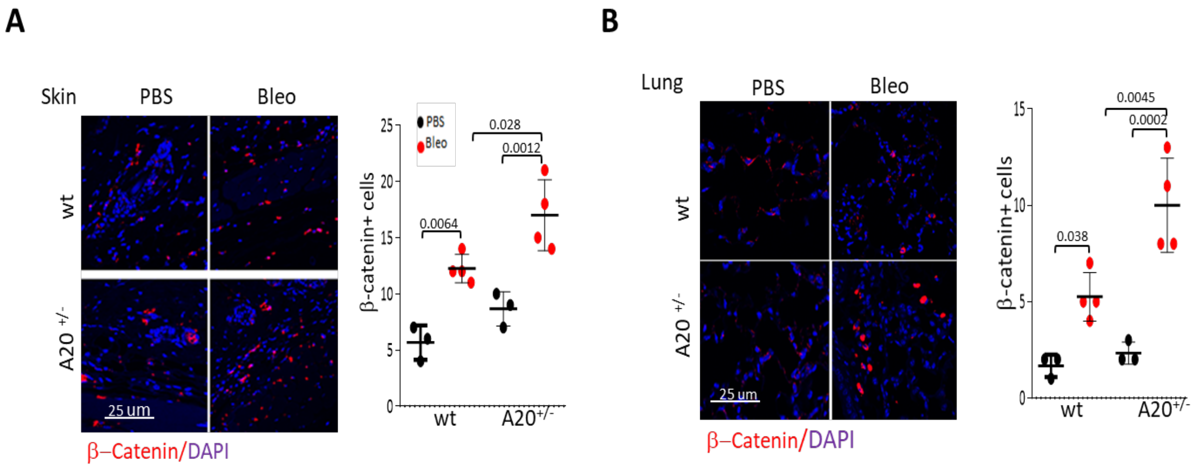

Supplementary Fig. 5. Bleomycin-treated A20$^{+/-}$ mice showed increased β-catenin accumulation in skin and lung. A20$^{fl/fl}$ mice and A20$^{+/-}$ mice in parallel were treated with s.c. bleomycin (10 mg/kg) or PBS for two weeks, and skin and lungs were harvested at day 22. Immunolabelling using antibodies to β-catenin. Number of immunopositive cells in the skin and lungs (A and B, right panels) determined from four randomly selected locations/hpf (n=2). Bars=25 µm. Results are means ± s.d. from three or four mice/group. One-way ANOVA followed by Sidak's multiple comparison test.

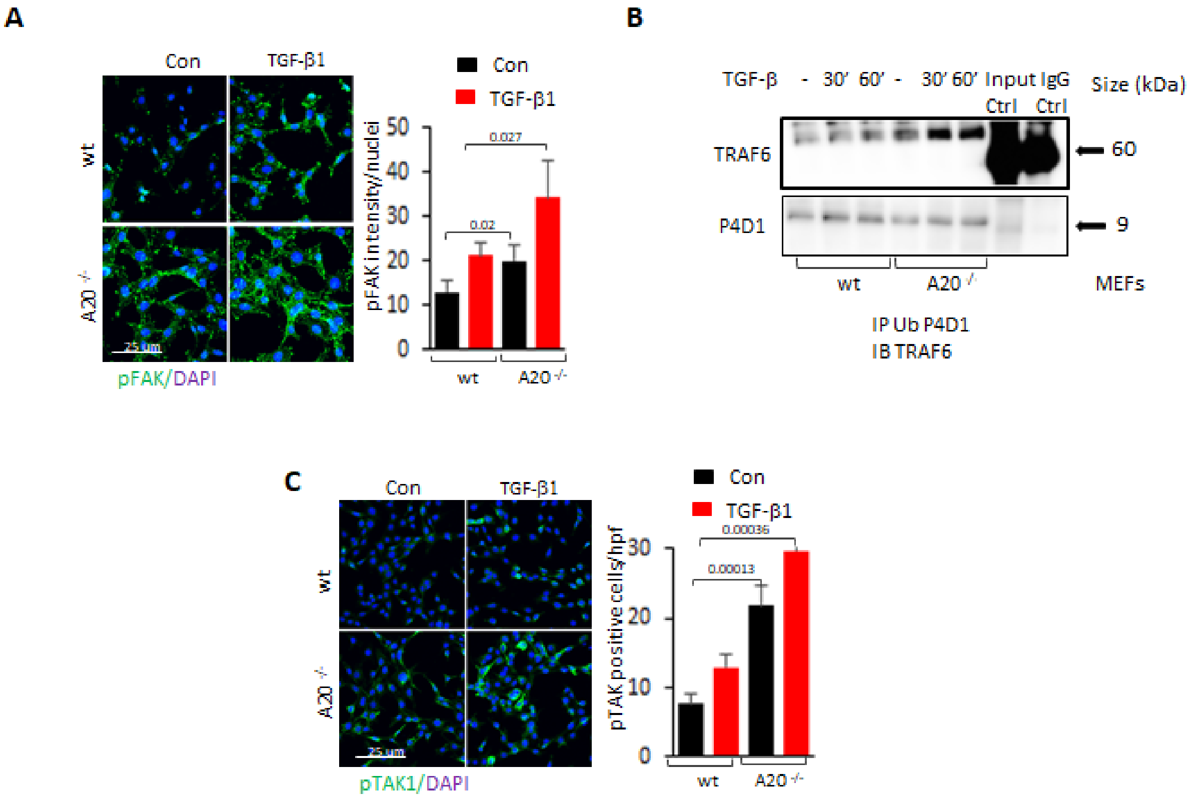

Supplementary Fig. 6. A20$^{-/-}$ murine embryonic fibroblasts showed increased FAK activation and TRAF6 ubiquitination. Confluent wild-type and A20$^{-/-}$ MEFs in parallel were treated with TGF-β1 for 30 min or indicated periods, and immunolabelled with antibodies to p-FAK (**A**) or p-TAK1 **(C).** Quantification of activated FAK (mean p-FAK) fluorescent intensity (A) and the numbers of p-TAK1+ cells/hpf (C) were determined from four randomly selected areas/hpf. Results are means ± s.d. from duplicate samples. One-way ANOVA followed by Sidak's multiple comparison test. **B.** Whole-cell lysates immunoprecipitated with antibodies to P4D1 followed by immunoblotting with antibodies to TRAF6 and ubiquitin (P4D1). Whole cell lysates with control IgG and IgG only without lysates were used as controls (n=2). The samples derive from the same experiment and blots were processed in parallel.

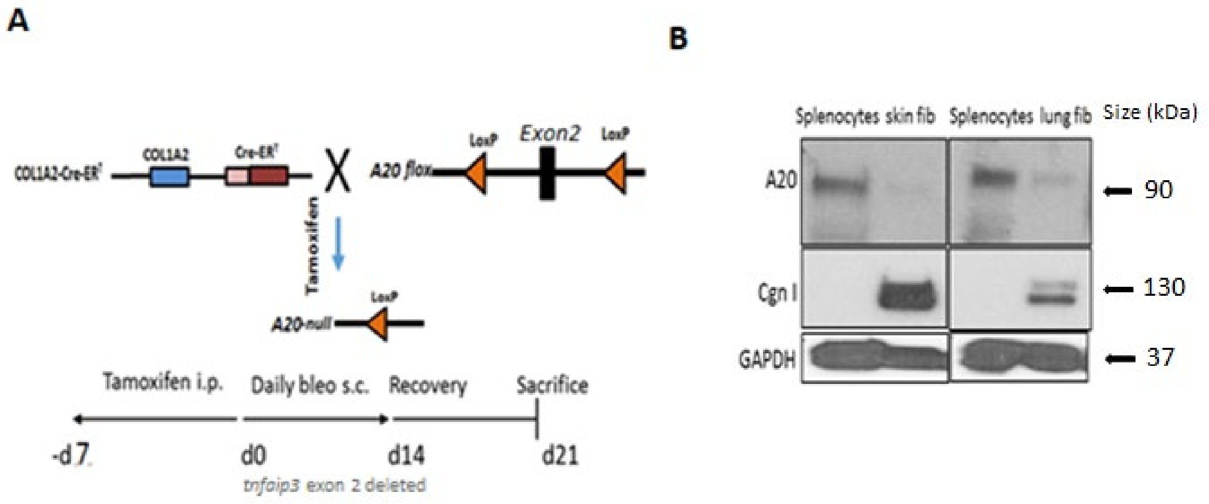

SUPPLEMENTARY FIG. 7. FIBROBLAST-SPECIFIC A20 ABLATION IN MICE. A. Schematic showing generation of mice with fibroblast-specific inducible A20 ablation (A20^{fibcko}) using COL1A2-CRE(ER)T**.** Fibroblast-specific A20 depletion was confirmed in 2 independent mice. **B.** Mice were sacrificed 7 days following last injection of tamoxifen or corn oil, and whole cell lysates from fibroblasts explanted from skin and lung, and splenocytes were explanted subjected to Western analysis using antibodies to A20. Representative immunoblots (n=2). The samples derive from the same experiment and blots were processed in parallel.

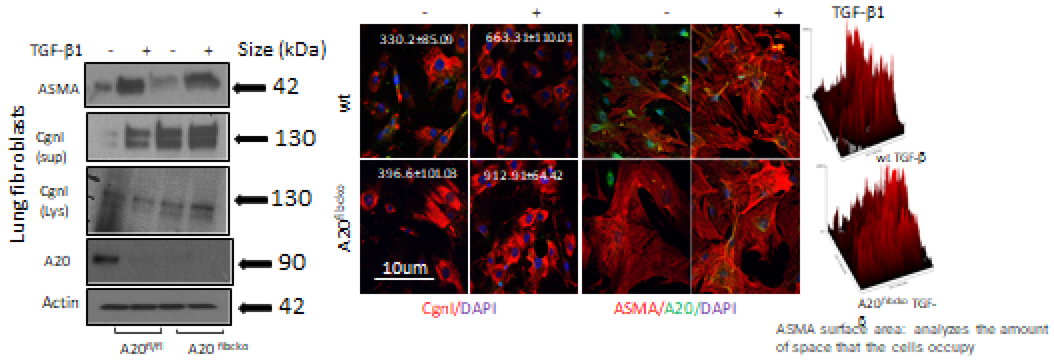

SUPPLEMENTARY FIG. 8. EXPLANTED A20^{fibcko} FIBROBLASTS SHOWED AUGMENTED FIBROTIC RESPONSES *IN VITRO*.

Lung fibroblasts were explanted from four-week-old A20$^{fl/fl}$ and A20^{fibcko} mice 7 days following final tamoxifen injection. Fibroblast cultures at confluence incubated with TGF-β1 for 24 h. Results were confirmed in two independent mice. The samples derive from the same experiment and blots were processed in parallel. **Left panels,** secreted media and whole cell lysates subjected to Western analysis. Representative immunoblots. **Right panels,** fibroblasts were immunolabelled using antibodies to Type I collagen (Cgn I), ASMA and A20. Representative immunofluorescent images; bars = 10 µm. Representative images; bars = 10 µm. Inset, Cgn I quantification (each point represents mean intensity from four randomly selected hpf). ASMA (surface area: analyzes the amount of space that the cells occupy was quantitated using ImageJ).

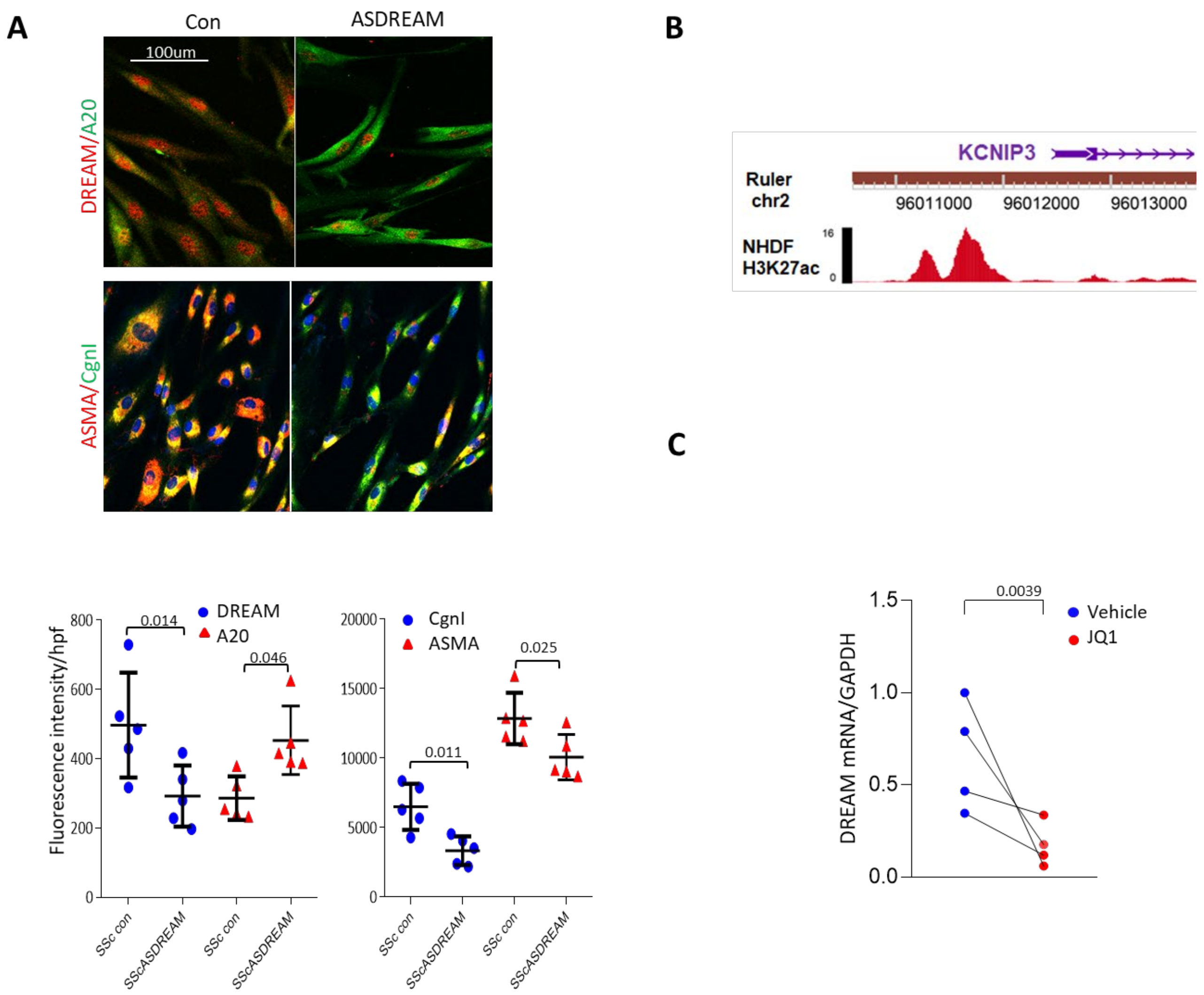

Supplementary Fig. 9. Expression of DREAM is regulated by epigenetic mechanisms in SSc fibroblasts. A. Subconfluent SSc fibroblasts (n=5) were transfected with antisense plasmid to knock down DREAM. Following 24 h incubation, fibroblasts were immunolabelled using antibodies to DREAM and A20 or Collagen I and ASMA. Upper panel: Representative images. Bars = 100 µm. Lower panels: Quantitation (each point represents mean immunofluorescence intensity from four randomly selected hpf/slide). Two-tailed Mann-Whitney U test. B. H3K27ac ChIP-seq tracks in normal human dermal fibroblasts (NHDF) extracted from ENCODE. C. Confluent cultures of SSc fibroblasts

(n=9) were incubated with JQ1 (1 μM) for 48 h, followed by qPCR. Results, normalized with actin, are means ± SD of triplicate determinations. Wilcoxon signed rank test, for paired samples.

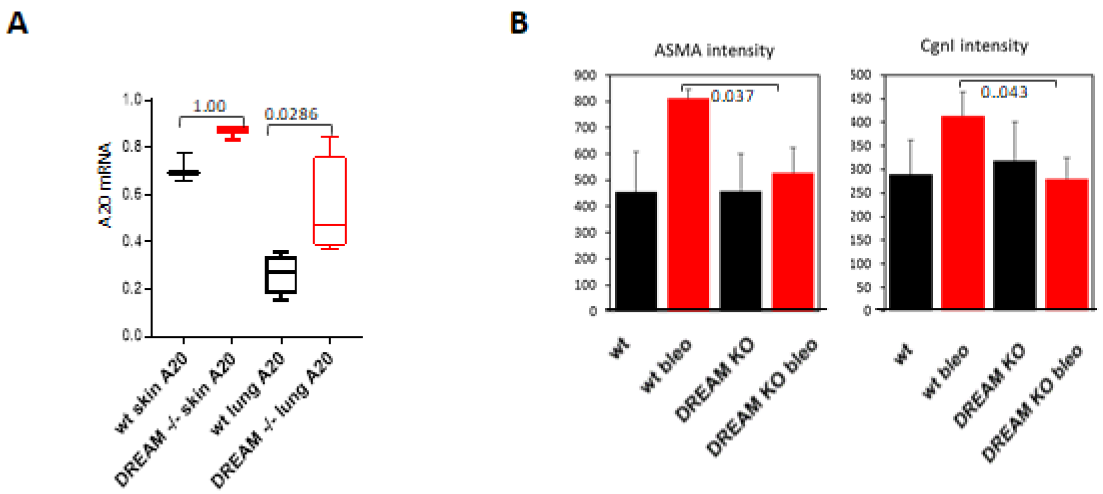

SUPPLEMENTARY FIG.10. DREAM$^{-/-}$ MICE SHOWED ATTENUATED BLEOMYCIN STIMULATION OF FIBROTIC RESPONSES IN THE SKIN AND LUNGS. DREAM$^{-/-}$ mice (n=4) and wildtype mice (n=3) were treated in parallel with s.c. bleomycin (10 mg/kg/d) or PBS for 2 weeks, and lesional skin was harvested at day 22. **A.** A20 mRNA expression in the skin and lungs from bleomycin-treated mice determined by qPCR. All data are presented as median ± IQR. p-values were determined by Kolmogorov-Smirnov test. **B.** Confluent lung fibroblasts explanted from DREAM$^{-/-}$ mice and wildtype mice were immunolabelled. Quantitation of fluorescence intensity (from Fig. 7F) from 4 hpf/section from each mouse (n=2). Two-tailed Student's t-test.

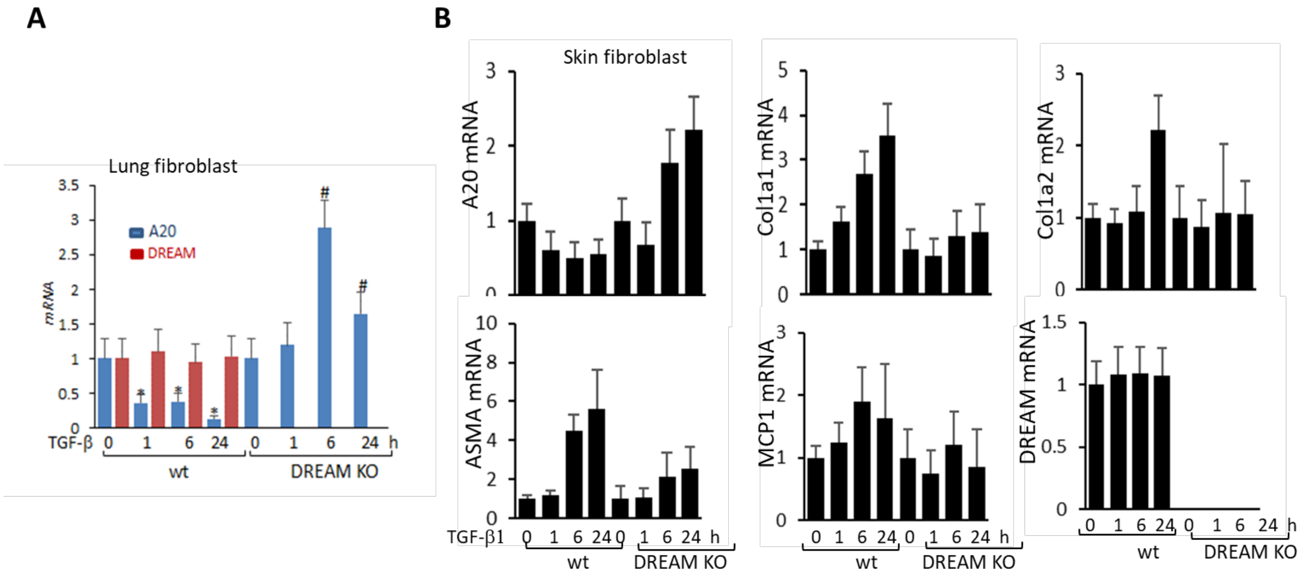

SUPPLEMENTARY FIG. 11. DREAM$^{-/-}$ FIBROBLASTS SHOWED ATTENUATED FIBROTIC RESPONSES. Lung (n=2) **(A)** and skin **(B)** (n=2) fibroblasts explanted from DREAM$^{-/-}$ and wild-type mice in parallel were grown to confluence and following incubation with TGF-β1 for indicated periods, mRNA levels were determined by qPCR. Results, normalized with GAPDH, are means ± SD of triplicate determinations from two mice/group.

Supplementary Data 1: Circulating IgG autoantibody production from PBS-treated $A20^{fl/fl}$ vs $A20^{+/-}$ mice

Four mice from $A20^{fl/fl}$ and five mice from $A20^{+/-}$ groups were studied.
Data provided in a separate excel file.

Supplementary Data 2A

Genome wide transcript changes in the skin from bleomycin and PBS-treated $A20^{fl/fl}$ mice

Three mice from $A20^{fl/fl}$ and $A20^{+/-}$ groups were studied. Genes with >2-fold increase or decrease and $p < 0.01$; FDR, 0.05 are shown. Data provided in a separate excel file.

Supplementary Data 2B

Genome wide transcript changes in the skin from bleomycin and PBS-treated $A20^{+/-}$ mice

Three mice from $A20^{fl/fl}$ and $A20^{+/-}$ groups were studied. Genes with >2-fold increase or decrease and $p < 0.01$; FDR, 0.05 are shown.

Data provided in a separate excel file.

Supplementary Table 1. KEGG pathway analysis of differentially expressed genes in skin from PBS-treated vs bleomycin-treated $A20^{+/-}$ mice

Databases: KEGG PATHWAY		
Statistical test method: hypergeometric test / one tailed Fisher's exact test		
FDR correction method: Benjamini and Hochberg		
#Term	Database ID	FDR P-Value
Cytokine-cytokine receptor interaction	KEGG PATHWAY	7.62E-17
Proteoglycans in cancer	KEGG PATHWAY	1.36E-15
Cell cycle	KEGG PATHWAY	1.74E-14
HTLV-I infection	KEGG PATHWAY	5.24E-12
Wnt signaling pathway	KEGG PATHWAY	3.22E-07
Dilated cardiomyopathy	KEGG PATHWAY	3.66E-11
PI3K-Akt signaling pathway	KEGG PATHWAY	2.49E-10
Hypertrophic cardiomyopathy (HCM)	KEGG PATHWAY	5.80E-09
Rheumatoid arthritis	KEGG PATHWAY	1.58E-09
Hippo signaling pathway	KEGG PATHWAY	7.59E-08
Focal adhesion	KEGG PATHWAY	1.04E-07
Phagosome	KEGG PATHWAY	1.08E-07
MAPK signaling pathway	KEGG PATHWAY	1.15E-07
Cell adhesion molecules (CAMs)	KEGG PATHWAY	2.18E-07
NF-kappa B signaling pathway	KEGG PATHWAY	4.58E-06
Jak-STAT signaling pathway	KEGG PATHWAY	1.32E-05

Chemokine signaling pathway	KEGG PATHWAY	2.27E-08
TNF signaling pathway	KEGG PATHWAY	9.42E-07
Leukocyte transendothelial migration	KEGG PATHWAY	8.65E-06
Toll-like receptor signaling pathway	KEGG PATHWAY	2.14E-05
ECM-receptor interaction	KEGG PATHWAY	3.75E-05

Supplementary Table 2: qPCR primer sequences

Gene	Primer sequence
Human	
h*A20*	Forward: 5'- TCCTCAGGCTTTGTATTTGAGC3 -3'<br>Reverse: 5'- TGTGTATCGGTGCATGGTTTTA3-3'
h*DREAM*	Forward: 5'- CTCATTTACGCGCAGTTCTTCC-3'<br>Reverse: 5'- GTCCGCATCAAAGGCGTTG -3'
h*B-ACTIN*	Forward: 5’-AATGTCGCGGAGGACTTTGAT-3’<br>Reverse: 5’-AGGATGGCAAGGGACTTCCTG-3’
Mouse	
m*Dream*	Forward: 5'- AGTGAACTGGAGTTATCCACGG -3′<br>Reverse: 5′- GTGAACTTGGTCTGAGCTTGT -3′
m*A20*	Forward: 5'- GCGGCCACAGAAAACACTC -3'<br>Reverse: 5'- CTCCCAATGGTCAAGGCATC-3'
m*Col1A2*	Forward: 5'- CCGTGCTTCTCAGAACATCA -3'<br>Reverse: 5'- CTTGCCCCATTCATTTGTCT -3'
m*IL1b*	Forward: 5'- CGAGGCTAATAGGCTCATCT -3'<br>Reverse: 5'- GTTTGGAAGCAGCCCTTCAT -3'
m*Col1A1*	Forward: 5'- AGCCGCAAAGAGTCTACATG -3′<br>Reverse: 5′- CTTAGGCCATTGTGTATGCAG -3′
m*IL6*	Forward: 5’- GAGGATACCACTCCCAACAGACC -3’<br>Reverse: 5’- AAGTGCATCATCGTTCATACA -3’
m*Mcp1*	Forward: 5’- AAAACACGGGACGAGAAACCC -3’<br>Reverse: 5’- ACGGGAACCTTTATTAACCCCT -3’
m*Gapdh*	Forward: 5'- ATCTTCTTGTGCAGTGCCAGC -3'<br>Reverse: 5'- GTTGATGGCAACAATCTCCAC -3'
m*Asma*	Forward: 5’- ATGCAGAAGGAGATCACAGC-3’<br>Reverse: 5’- GTATTCCTGTTTGCTGATCCAC-3’

Supplementary Data 1

IgG ratio									
	A20$^{fl/fl}$(wt)	A20 $^{fl/fl}$(wt)	A20 $^{fl/fl}$ (wt)	A20 $^{fl/fl}$ (wt)	A20$^{+/-}$	A20$^{+/-}$	A20$^{+/-}$	A20$^{+/-}$	A20$^{+/-}$
**ID**	**M49_1**	**M49_2**	**M49_3**	**M49_4**	**M49_11**	**M49_12**	**M49_13**	**M49_14**	**M49_15**
Aggrecan	5.22	6.84	6.4	5.8	7.62	8.09	8.87	7.38	6.97
AGTR	12.35	11.69	12.1	10.46	14.04	14.26	14.59	11.76	12.39
alpha-actinine	0	0	0	4.71	0	0	0	4.76	0
Alpha Fodrin	11.67	14.54	12.74	10.99	14.58	12.43	12.99	13.32	13.03
Amyloid	1.68	2.13	1.5	0	1.68	0	0.22	1.13	0
AQP4	13.85	12.61	12.44	11.41	12.88	13.75	14.05	12.34	13.05
B2-microglobulin	6.5	7.39	6.71	7.48	7.39	8.46	7.46	7.55	7.03
B2 glycoprotein 1	15.14	15.49	16.06	15.93	17.06	17.07	17.93	16.06	17.22
BPI	17.67	17.89	17.95	17.17	16.89	18.36	18	16.43	18.55
Cardolipin	4.95	4.1	0.17	2.3	5.63	4.39	4.05	3.6	3.88
CENP-A	10.47	10	7.44	8.44	9.58	12.26	12.12	11.15	11.28
CENP-B	13.65	13.09	13.05	13.73	15.59	16.15	17.7	14.55	15.95
Chondroitin Sulfate C	5.28	4.84	2.24	3.73	5.58	5.66	4.72	3.95	3.5
Collagen I	9.08	8.76	6.84	8.28	10.38	9.59	9.85	9.12	8.51
Collagen II	8.72	7.42	7.16	6.07	7.39	7.34	7.04	7.6	6.73
Collagen III	10.14	8.85	8.09	7.87	9.94	8.84	9.07	8.69	8.71
Collagen IV	11.8	10.76	11.14	9.95	13.01	11.22	12.31	13.27	11.29
Collagen V	12.81	11.99	12.22	12.3	12.28	12.84	12.65	12.48	12.69
Collagen VI	16.42	16.18	15.95	15.91	18.27	17.76	17.68	15.71	17.83
complement C1q	16.97	16.21	16.96	16.52	17.94	19.18	17.86	16.86	17.77
complement C3	17.22	16.48	17.74	17.05	19.66	16.58	17.89	16.71	16.41
complement C3a	12.1	11.85	12.46	11.5	13.58	12.73	13.43	12.12	13.43
complement C4	15.27	15.51	15.32	15.28	15.89	16.04	16.31	15.3	15.67
complement C5	16.02	15.92	16.63	15.83	16.07	16.62	15.84	15.13	16.85
complement C6	16.67	17.05	17.15	16.11	17.13	17.31	15.27	16	16.13
complement C7	15.67	15.28	15.44	15.63	16.09	16.72	15.84	14.63	16.21
complement C8	16.04	15.68	15.56	15.42	18.18	16.55	15.62	15.26	16.18
complement C9	16.23	15.55	16.35	15.55	15.28	16.46	16.56	15.41	15.4
Core Histone	12.85	12.11	13.28	11.33	12.21	13.14	13.23	12.72	12.71
CRP	19.88	19.69	21.13	18.47	19.12	20.92	19.6	18.92	19.84
Cytochrome C	9.34	9.36	7.63	8.87	9.47	10.25	10.16	9.39	10.72
Decorin-bovine	5.18	5.64	6.37	2.94	5.71	5.57	4.6	4.63	4.58
DGPS	6.04	5.63	3.16	0.97	6.4	5.06	4.98	0	4.8
DNA Polymerase beta (P[illegible]	14.22	13.98	14.71	13.46	16	15.02	15.18	14	14.79
dsDNA	6.04	7.67	8.36	7.77	13.91	11.08	10.96	9.35	10.92
EBNA1	9.77	8	4.84	4.5	8.53	6.38	4.93	9.97	9.03
Elastin	9.53	9.46	10.05	9.2	11.16	10.5	11.45	9.98	9.64

Entaktin EDTA	11.32	9.77	10.68	11.05	13.01	12.65	12.53	11.98	10.96
Factor B	16.15	15.62	14.84	15.7	16.96	15.64	16.22	14.63	14.46
Factor D	10.22	9.35	9.02	9.7	10.25	10.23	10.83	9.32	10.06
Factor H	15.43	15.43	15.52	15.35	16.81	16.01	16.36	14.91	15
Factor I	13.76	12.81	13.93	13.49	16.28	15.02	13.78	13.8	13.9
Factor P	6.93	6.14	5.8	6.51	7.78	6.75	7.29	5.34	7.77
Fibrinogen IV	13.73	13.62	13.55	12.97	12.71	14.58	15.27	14.07	13.59
Fibrinogen S	14.1	14.39	13.94	14.55	14.15	15.92	15.05	14.88	13.7
Fibronectin	13.83	13.49	13.36	13.52	14.93	14.24	14.72	14.47	14.24
GBM	12.21	11.33	10.87	11.44	12.86	13.43	13.46	12.16	13.71
Genomic DNA	2.99	4.47	0.92	0	7.95	3.44	5.71	0.42	4.74
Gliadin	12.92	12.71	12.58	16.59	16.78	15.9	14.06	13.22	13.85
Glycated Albumin	15.4	14.71	15.31	13.78	15.29	15.33	15.09	14.04	14.06
GP2	14.39	14.06	14.67	14.53	15.28	16.25	16.24	15.28	17.04
GP210	10.17	9.02	8.43	8.97	11.2	11.46	9.99	9.57	9.88
Hemocyanin	17.11	16.47	17.39	16.65	17.15	18.38	17.69	17.55	16.91
Heparan sulfate proteogly	10.95	9.82	10.86	10.62	12.15	12.72	12.27	10.96	11.93
Heparan Sulphate	6.53	7.68	6.23	6.56	7.66	7.73	7.58	6.71	7.27
Heparin	4.95	4.5	4.84	3.53	6.72	5.75	5.75	4.82	4.85
Histone H1	13.52	14.25	14.53	14.18	14.99	15.07	15.51	13.9	16.07
Histone H2A	14.5	13.08	14.68	13.3	15.8	15.11	14.54	14.05	14.56
Histone H2B	14.67	13.21	15.59	13.43	16.73	15.76	16.19	14.38	14.68
Histone H3	10.16	9.76	8.78	8.97	11.08	11.85	11.53	10.08	10.9
Histone H4	8.33	7.86	8.17	7.2	9.78	9.73	9.45	8.35	9.37
Insulin	11.07	9.85	10.78	10.49	12.27	12.53	13.08	11.36	12.72
Intrinsic Factor	14.37	13.57	15.07	13.92	14.75	15.2	14.75	13.24	13.14
Jo-1	18.9	17.57	18.64	17.63	23.13	18.79	18.93	18.47	18.18
KU (P70/P80)	15.56	15.52	15.42	15.22	15.94	15.79	15.64	15.27	15.38
La/SSB	16.79	16.89	17.55	17.08	16.08	17.15	17.14	15.42	16.53
Laminin	9.94	9.29	9.76	9.53	10.81	10.67	10.9	10.01	10.65
LC1	17.37	17.54	17.94	17.46	15.63	17.88	17.43	17.54	18.21
LKM1	8.88	8.82	8.42	7.76	10.32	9.43	10.17	7.77	9.81
LPS	4.23	3.51	5.44	2.78	6.2	4.7	6.16	5.48	4.72
M2	15.31	14.55	15.62	14.91	16.33	16.64	16.5	15.56	15.36
Matrigel	13.8	13.68	13.79	13.3	15.28	14.71	15.43	13.56	14.13
MDA5	12.51	12.09	12.83	12.41	14.24	13.44	13.01	11.68	12.68
Mi-2	8.62	7.58	7.4	7.44	10.48	9.23	9.72	7.7	10.17
Mitochondrial antigen	13.68	14.23	14.21	13.25	15.79	14.58	14.7	13.96	13.57
MPO	14.42	13.67	14.18	14.19	15.32	16.24	15.84	14.3	13.18
Muscarinic receptor	16.98	16.4	18.21	15.68	17.85	17.31	17.41	16.57	16.58
Myelin basic protein (MBI	14.11	15.9	18.06	16.03	18.18	17.48	18.34	16.5	17.25

Myosin	15.62	15.7	15.88	15.23	15.17	16.26	16.25	14.79	15.28
Nucleolin	11.88	11.25	10.32	11.11	11.21	12.74	10.93	11.75	13
Nucleosome antigen	16.49	14.26	17.08	14.89	17.11	16.53	17.54	16.83	18.26
Nup 62	7.93	8	7.91	6.98	8.8	8.74	8.78	9.35	8.68
PCNA	16.71	16.68	17.33	16.08	17.13	17.63	16.82	16.44	16.39
Peroxiredoxin 1	16.74	16.51	16.75	16.12	17.82	17.72	16.4	16.41	16.57
PL-7	18.67	18.73	19	17.71	19.36	18.44	18.45	17.11	18.26
PL-12	12.77	11.87	13.19	12.67	13.65	14.13	13.68	13.02	13.98
PM/Scl-75	13.35	13	14.18	13.48	13.75	14.61	14.98	13.53	18.57
PM/Scl 100	16.63	15.89	17.44	16.83	16.53	17.26	17.71	15.5	17.96
PR3	8.88	7.38	8.2	7.53	10.52	10.09	9.99	8.38	9.4
Proteoglycan	7.74	7.05	7.96	3.5	8.46	6.99	8.36	6.72	7.59
Prothrombin protein	16.09	15.85	16.02	17.53	16.93	17.31	17.52	15.75	16.66
Ribo Phosphoprotein P0	15.68	15.36	15.26	14.83	15.82	16.67	15.74	16.01	15.05
Ribo Phosphoprotein P1	9.52	9.4	7.76	8.67	9.04	10.26	8.11	9.58	9.69
Ribo Phosphoprotein P2	8.75	8.84	9.54	8.74	11.21	9.73	9.33	8.58	9.91
Ro/SSA (52 Kda)	18.09	17.34	17.98	17.17	18.43	18.67	17.8	18	17.9
Ro/SSA (60 Kda)	15	14.82	14.85	14.3	15.05	15.51	14.81	14.6	13.89
S100	16.81	15.27	16.05	15.26	16.54	14.99	16.15	14.86	16.78
Scl-70/Topoisomerase I	16.4	16.49	16.74	15.95	16.23	17.3	17.52	15.35	14.52
Sm	8.05	7.24	6.97	7.05	9.82	10.18	9.15	7.57	8.58
Sm/RNP	12.95	12.23	12.02	12.42	13.92	13.79	13.8	12.49	12.55
SmD	9.81	8.68	8.74	8.72	10.85	11.21	10.1	8.23	9.26
SmD1	13.47	11.73	11.93	11.53	15.11	13.91	14.66	12.52	13.23
SmD2	8.47	8.12	10.08	9.24	12.34	11.75	11.66	11.56	10.69
SmD3	9.84	9.14	9.67	9.25	11.9	11.67	10.68	9.36	8.44
SP100	7.36	7.01	6.18	5.89	9.14	8.63	10.39	7.17	8.43
Sphingomyelin	2.62	2.32	3.15	0	1.95	0.37	0.49	1.11	0.97
SRP54	9.19	8.8	7.59	8.64	9.81	9.68	9.54	8.73	8.69
ssDNA	15.47	16.1	14.34	16.13	20.88	17.7	19.44	16.28	17.11
ssRNA	2.94	0	1.3	5.15	4.13	3.7	1.08	2.17	1.57
T1F1 gama	13.09	11.42	12.8	12.17	13.53	14.12	13.89	12.49	12.73
Thyroglobulin	15.07	15.09	14.77	14.8	16.56	15.97	16.62	15.15	15.54
TNF-?	17.57	17.03	17.63	16.73	17.2	18.32	17.71	17.18	21.86
TPO	16.3	15.63	16.67	16.34	16.1	17.19	17	16.41	17.14
TTG	17.05	17.56	17.47	17.35	16.48	18.86	19.27	16.87	16.04
U1-snRNP 68/70	14.72	13.81	13.42	13.32	15.18	15.15	16.77	14.13	18.38
U1-snRNP A	14.19	12.37	12.87	13.29	14.75	14.97	16.19	13.67	14.39
U1-snRNP B/B'	14.24	12.81	12.73	12.68	14.77	15.38	13.9	12.83	14.69
U1-snRNP C	15.18	14.21	13.75	14.19	16.32	15.29	14.58	14.57	14.58
Vimentin	7.46	6.76	8.13	7.34	9.58	9.64	9.25	8.58	9.22

Vitronectin	13.14	12.64	13.1	12.64	14.78	14.28	13.56	12.96	13.96

Supplementary Data 2A

Blast swiss prot (up>2-FC 170 genes)	log2 FoldChange	pval	padj
sp|P50228|CXCL5_MOUSE C-X-C motif chemokine 5 OS=Mus musculus OX=10090 GN=Cxcl5 PE=1 SV=2//3.25721e-72	6.7611	0.0035381	0.027473
sp|Q91Z83|MYH7_MOUSE Myosin-7 OS=Mus musculus OX=10090 GN=Myh7 PE=2 SV=1//0	6.7149	0.000032954	0.00053019
-//-	5.8109	9.54E-23	3.94E-20
sp|P52430|PON1_MOUSE Serum paraoxonase/arylesterase 1 OS=Mus musculus OX=10090 GN=Pon1 PE=1 SV=2//7.31539e-13	5.4306	1.39E-07	4.02E-06
sp|P19123|TNNC1_MOUSE Troponin C, slow skeletal and cardiac muscles OS=Mus musculus OX=10090 GN=Tnnc1 PE=1 SV=1//2.84479e-100	4.7545	4.85E-13	3.89E-11
sp|Q80SU7|GVIN1_MOUSE Interferon-induced very large GTPase 1 OS=Mus musculus OX=10090 GN=Gvin1 PE=1 SV=1//0	4.5869	0.0070586	0.047643
sp|P13541|MYH3_MOUSE Myosin-3 OS=Mus musculus OX=10090 GN=Myh3 PE=2 SV=2//0	4.5212	1.00E-10	5.38E-09
sp|P56388|CART_MOUSE Cocaine- and amphetamine-regulated transcript protein OS=Mus musculus OX=10090 GN=Cartpt PE=1 SV=2//1.54566e-65	4.51	0.0021219	0.018162
sp|Q2Q5T5|MYMX_MOUSE Protein myomixer OS=Mus musculus OX=10090 GN=Mymx PE=1 SV=1//5.46971e-21	4.4986	0.000035762	0.00056747
sp|P24699|MYF5_MOUSE Myogenic factor 5 OS=Mus musculus OX=10090 GN=Myf5 PE=1 SV=1//6.31678e-161	4.3786	2.48E-08	8.27E-07
-//-	4.1719	0.0011189	0.01067
sp|P33435|MMP13_MOUSE Collagenase 3 OS=Mus musculus OX=10090 GN=Mmp13 PE=1 SV=1//0	4.1586	2.85E-25	1.54E-22
sp|P01631|KV2A7_MOUSE Ig kappa chain V-II region 26-10 OS=Mus musculus OX=10090 PE=1 SV=1//1.25525e-65	4.1583	0.0011601	0.011013
sp|Q6DFV6|FN3C1_MOUSE Fibronectin type III domain containing protein 3C1 OS=Mus musculus OX=10090 GN=Fndc3c1 PE=2 SV=1//0	4.0238	0.00015955	0.0020656
sp|Q61468|MSLN_MOUSE Mesothelin OS=Mus musculus OX=10090 GN=Msln PE=1 SV=1//0	4.0007	1.22E-06	0.000028816
sp|Q6P3A1|STMD1_MOUSE Stathmin domain-containing protein 1 OS=Mus musculus OX=10090 GN=Stmnd1 PE=2 SV=1//6.86249e-171	3.9668	0.00056963	0.0060722
sp|P35329|CD22_MOUSE B-cell receptor CD22 OS=Mus musculus OX=10090 GN=Cd22 PE=1 SV=1//0	3.9465	0.000024145	0.00040313
sp|O35488|S27A2_MOUSE Very long-chain acyl-CoA synthetase OS=Mus musculus OX=10090 GN=Slc27a2 PE=1 SV=2//0	3.9213	0.0040595	0.03065
sp|Q05421|CP2E1_MOUSE Cytochrome P450 2E1 OS=Mus musculus OX=10090 GN=Cyp2e1 PE=1 SV=1//0	3.9061	3.68E-08	1.18E-06
sp|Q9CR42|ANKR1_MOUSE Ankyrin repeat domain-containing protein 1 OS=Mus musculus OX=10090 GN=Ankrd1 PE=1 SV=1//0	3.8056	3.75E-43	9.13E-40
sp|P13542|MYH8_MOUSE Myosin-8 OS=Mus musculus OX=10090 GN=Myh8 PE=2 SV=2//0	3.7808	3.41E-20	8.65E-18
sp|P51590|CP2J3_RAT Cytochrome P450 2J3 OS=Rattus norvegicus OX=10116 GN=Cyp2j3 PE=2 SV=1//0	3.7546	0.0020009	0.017328
sp|P04939|MUP3_MOUSE Major urinary protein 3 OS=Mus musculus OX=10090 GN=Mup3 PE=1 SV=1//1.0818e-110	3.7478	9.68E-09	3.56E-07
sp|P01644|KV5AB_MOUSE Ig kappa chain V-V region HP R16.7 OS=Mus musculus OX=10090 PE=1 SV=1//3.88253e-63	3.6755	0.0022461	0.019005
sp|Q6P640|ACTC_XENTR Actin, alpha cardiac muscle 1 OS=Xenopus tropicalis OX=8364 GN=actc1 PE=2 SV=1//0	3.6238	3.49E-19	7.45E-17
sp|P12979|MYOG_MOUSE Myogenin OS=Mus musculus OX=10090 GN=Myog PE=1 SV=2//3.2593e-148	3.6065	1.03E-10	5.50E-09
sp|P47239|PAX7_MOUSE Paired box protein Pax-7 OS=Mus musculus OX=10090 GN=Pax7 PE=1 SV=2//0	3.6003	3.56E-11	2.12E-09
sp|Q60673|PTPRN_MOUSE Receptor-type tyrosine-protein phosphatase-like N OS=Mus musculus OX=10090 GN=Ptprn PE=1 SV=2//0	3.5961	0.00006126	0.00090426
sp|Q5TFQ8|SIRBL_HUMAN Signal-regulatory protein beta-1 isoform 3 OS=Homo sapiens OX=9606 GN=SIRPB1 PE=1 SV=1//2.04372e-144	3.5225	0.00067342	0.0069917
sp|P05534|1A24_HUMAN HLA class I histocompatibility antigen, A-24 alpha chain OS=Homo sapiens OX=9606 GN=HLA-A PE=1 SV=2//6.57368e-44	3.5131	0.0021214	0.018162
sp|Q38PU4|GRIK1_MACFA Glutamate receptor ionotropic, kainate 1 OS=Macaca fascicularis OX=9541 GN=GRIK1 PE=2 SV=1//0	3.4849	1.12E-07	3.31E-06
sp|Q9D1N4|MYMK_MOUSE Protein myomaker OS=Mus musculus OX=10090 GN=Mymk PE=1 SV=1//2.39868e-121	3.3725	7.91E-07	0.000019366
sp|A2BIM8|MUP18_MOUSE Major urinary protein 18 OS=Mus musculus OX=10090 GN=Mup18 PE=3 SV=1//2.21262e-116	3.3108	3.07E-25	1.62E-22
-//-	3.2548	0.00094056	0.0092184
sp|P02716|ACHD_MOUSE Acetylcholine receptor subunit delta OS=Mus musculus OX=10090 GN=Chrnd PE=2 SV=1//0	3.2166	1.10E-09	4.92E-08
sp|P51480|CDN2A_MOUSE Cyclin-dependent kinase inhibitor 2A OS=Mus musculus OX=10090 GN=Cdkn2a PE=1 SV=2//1.57272e-86	3.161	4.50E-07	0.000011757
sp|P50446|K2C6A_MOUSE Keratin, type II cytoskeletal 6A OS=Mus musculus OX=10090 GN=Krt6a PE=1 SV=3//0	3.1554	8.46E-08	2.56E-06
sp|P70375|FA7_MOUSE Coagulation factor VII OS=Mus musculus OX=10090 GN=F7 PE=1 SV=1//0	3.1553	5.38E-07	0.000013693
sp|Q8VCR2|DHB13_MOUSE 17-beta-hydroxysteroid dehydrogenase 13 OS=Mus musculus OX=10090 GN=Hsd17b13 PE=1 SV=2//0	3.1165	2.99E-06	0.000063489

sp|Q7TNB2|TNNT1_RAT Troponin T, slow skeletal muscle OS=Rattus norvegicus OX=10116 GN=Tnnt1 PE=1 SV=3//1.4801e-88	3.1024	5.50E-11	3.10E-09
sp|Q9Z2H6|CLC4D_MOUSE C-type lectin domain family 4 member D OS=Mus musculus OX=10090 GN=Clec4d PE=1 SV=1//3.94725e-160	3.0885	0.00078201	0.0078935
sp|O55188|DMP1_MOUSE Dentin matrix acidic phosphoprotein 1 OS=Mus musculus OX=10090 GN=Dmp1 PE=2 SV=2//1.68168e-166	3.0798	0.002769	0.022642
sp|Q03059|CLAT_MOUSE Choline O-acetyltransferase OS=Mus musculus OX=10090 GN=Chat PE=2 SV=2//0	3.0733	4.16E-08	1.33E-06
sp|Q9Z0J7|GDF15_MOUSE Growth/differentiation factor 15 OS=Mus musculus OX=10090 GN=Gdf15 PE=1 SV=2//2.21971e-156	3.0635	0.00022562	0.0027804
sp|Q80X76|SPA3F_MOUSE Serine protease inhibitor A3F OS=Mus musculus OX=10090 GN=Serpina3f PE=1 SV=3//8.79452e-09	2.9833	0.0015101	0.013693
sp|Q9JKB0|HCN1_RAT Potassium/sodium hyperpolarization-activated cyclic nucleotide-gated channel 1 OS=Rattus norvegicus OX=10116 GN=Hcn1 PE=2 SV=1//0	2.9441	0.0024024	0.020124
sp|B5X0G2|MUP17_MOUSE Major urinary protein 17 OS=Mus musculus OX=10090 GN=Mup17 PE=2 SV=2//2.4009e-116	2.9401	4.13E-16	5.49E-14
sp|Q6SJQ7|CLM1_MOUSE CMRF35-like molecule 1 OS=Mus musculus OX=10090 GN=Cd300lf PE=1 SV=1//7.41183e-82	2.9253	0.000020348	0.00034688
sp|P97797|SHPS1_MOUSE Tyrosine-protein phosphatase non-receptor type substrate 1 OS=Mus musculus OX=10090 GN=Sirpa PE=1 SV=1//3.17488e-149	2.9133	8.76E-06	0.00016478
sp|P09541|MYL4_MOUSE Myosin light chain 4 OS=Mus musculus OX=10090 GN=Myl4 PE=1 SV=3//1.53837e-24	2.9059	1.16E-10	6.13E-09
sp|P10923|OSTP_MOUSE Osteopontin OS=Mus musculus OX=10090 GN=Spp1 PE=1 SV=1//4.69326e-165	2.8872	1.92E-11	1.19E-09
sp|P09535|IGF2_MOUSE Insulin-like growth factor II OS=Mus musculus OX=10090 GN=Igf2 PE=1 SV=1//1.30256e-90	2.8691	2.04E-19	4.54E-17
sp|P04756|ACHA_MOUSE Acetylcholine receptor subunit alpha OS=Mus musculus OX=10090 GN=Chrna1 PE=1 SV=1//0	2.856	2.80E-07	7.69E-06
sp|P09240|CCKN_MOUSE Cholecystokinin OS=Mus musculus OX=10090 GN=Cck PE=1 SV=3//7.27695e-57	2.8201	5.40E-08	0.000001686
sp|Q9ERZ3|ACM3_MOUSE Muscarinic acetylcholine receptor M3 OS=Mus musculus OX=10090 GN=Chrm3 PE=1 SV=1//0	2.8049	0.0027612	0.022593
sp|Q07440|B2LA1_MOUSE Bcl-2-related protein A1 OS=Mus musculus OX=10090 GN=Bcl2a1 PE=1 SV=1//9.23322e-117	2.7957	0.0039812	0.030217
sp|P11588|MUP1_MOUSE Major urinary protein 1 OS=Mus musculus OX=10090 GN=Mup1 PE=1 SV=1//1.35863e-108	2.7694	1.09E-11	7.11E-10
sp|P34960|MMP12_MOUSE Macrophage metalloelastase OS=Mus musculus OX=10090 GN=Mmp12 PE=1 SV=3//9.58652e-15	2.7398	6.36E-13	5.04E-11
sp|Q6Q473|CLA4A_MOUSE Calcium-activated chloride channel regulator 4A OS=Mus musculus OX=10090 GN=Clca4a PE=1 SV=2//0	2.7144	0.0017027	0.015145
sp|Q6H1V1|BEST3_MOUSE Bestrophin-3 OS=Mus musculus OX=10090 GN=Best3 PE=2 SV=1//0	2.7005	0.00021613	0.0026852
sp|Q14BP6|LR74B_MOUSE Leucine-rich repeat-containing protein 74B OS=Mus musculus OX=10090 GN=Lrrc74b PE=2 SV=1//0	2.6377	0.0027824	0.022736
sp|Q00780|CO8A1_MOUSE Collagen alpha-1(VIII) chain OS=Mus musculus OX=10090 GN=Col8a1 PE=1 SV=3//2.98412e-35	2.6321	1.61E-07	4.63E-06
sp|O88940|MUSC_MOUSE Musculin OS=Mus musculus OX=10090 GN=Msc PE=1 SV=1//4.97526e-93	2.6306	0.007406	0.049699
sp|O70578|CCG1_MOUSE Voltage-dependent calcium channel gamma-1 subunit OS=Mus musculus OX=10090 GN=Cacng1 PE=1 SV=1//1.54124e-117	2.6122	8.54E-21	2.53E-18
sp|Q9D5Z5|MSS51_MOUSE Putative protein MSS51 homolog, mitochondrial OS=Mus musculus OX=10090 GN=Mss51 PE=2 SV=1//0	2.5705	0.0016814	0.014988
sp|Q6SLE7|SARCO_RAT Sarcolipin OS=Rattus norvegicus OX=10116 GN=Sln PE=3 SV=1//4.65951e-12	2.567	1.04E-30	9.77E-28
sp|Q8C8H8|KY_MOUSE Kyphoscoliosis peptidase OS=Mus musculus OX=10090 GN=Ky PE=1 SV=1//0	2.5548	1.96E-34	2.99E-31
sp|Q8BGM5|BEST2_MOUSE Bestrophin-2 OS=Mus musculus OX=10090 GN=Best2 PE=2 SV=1//0	2.5154	0.0054486	0.039064
-//-	2.5136	9.31E-20	2.24E-17
-//-	2.4961	5.20E-16	6.77E-14
sp|P70263|PD2R_MOUSE Prostaglandin D2 receptor OS=Mus musculus OX=10090 GN=Ptgdr PE=2 SV=1//0	2.4823	4.61E-07	0.000011975
sp|P12850|GROA_MOUSE Growth-regulated alpha protein OS=Mus musculus OX=10090 GN=Cxcl1 PE=1 SV=1//3.07076e-43	2.4817	0.0039151	0.029815
sp|Q06318|UTER_MOUSE Uteroglobin OS=Mus musculus OX=10090 GN=Scgb1a1 PE=1 SV=1//1.89394e-52	2.473	3.53E-06	0.000073794
sp|Q99N05|M4A4D_MOUSE Membrane-spanning 4-domains subfamily A member 4D OS=Mus musculus OX=10090 GN=Ms4a4d PE=2 SV=1//1.15524e-108	2.4539	1.07E-17	1.75E-15
sp|Q9QY96|CASR_MOUSE Extracellular calcium-sensing receptor OS=Mus musculus OX=10090 GN=Casr PE=1 SV=2//0	2.4426	7.16E-07	0.000017707
sp|Q62282|TAL2_MOUSE T-cell acute lymphocytic leukemia protein 2 homolog OS=Mus musculus OX=10090 GN=Tal2 PE=4 SV=1//2.94553e-59	2.4381	5.49E-06	0.00010951
sp|P11588|MUP1_MOUSE Major urinary protein 1 OS=Mus musculus OX=10090 GN=Mup1 PE=1 SV=1//3.97646e-117	2.4371	2.36E-08	7.91E-07
sp|Q5SS00|ZDBF2_MOUSE DBF4-type zinc finger-containing protein 2 homolog OS=Mus musculus OX=10090 GN=Zdbf2 PE=2 SV=1//3.7793e-19	2.4349	0.00013298	0.0017657
sp|P02762|MUP6_MOUSE Major urinary protein 6 OS=Mus musculus OX=10090 GN=Mup6 PE=1 SV=2//3.61094e-110	2.4307	0.00036311	0.004143
sp|Q9D300|RGF1C_MOUSE Ras-GEF domain-containing family member 1C OS=Mus musculus OX=10090 GN=Rasgef1c PE=2 SV=1//0	2.4095	0.00023113	0.002841

sp|Q96HQ0|ZN419_HUMAN Zinc finger protein 419 OS=Homo sapiens OX=9606 GN=ZNF419 PE=1 SV=2//2.86511e-17	2.3882	0.0022458	0.019005
sp|P01837|IGKC_MOUSE Immunoglobulin kappa constant OS=Mus musculus OX=10090 GN=Igkc PE=1 SV=2//2.11599e-64	2.3848	0.000006806	0.00013203
sp|Q64282|IFIT1_MOUSE Interferon-induced protein with tetratricopeptide repeats 1 OS=Mus musculus OX=10090 GN=Ifit1 PE=1 SV=2//0	2.3819	7.42E-07	0.000018222
-//-	2.3781	3.96E-07	0.000010505
sp|P70549|NAC3_RAT Sodium/calcium exchanger 3 OS=Rattus norvegicus OX=10116 GN=Slc8a3 PE=1 SV=1//0	2.3698	1.32E-20	3.79E-18
sp|Q8BV66|IFI44_MOUSE Interferon-induced protein 44 OS=Mus musculus OX=10090 GN=Ifi44 PE=2 SV=1//6.17701e-16	2.3366	0.00042252	0.0047104
sp|P04760|ACHG_MOUSE Acetylcholine receptor subunit gamma OS=Mus musculus OX=10090 GN=Chrng PE=2 SV=1//0	2.3209	0.00047731	0.0052302
sp|P70610|DOC2B_RAT Double C2-like domain-containing protein beta OS=Rattus norvegicus OX=10116 GN=Doc2b PE=1 SV=2//0	2.3175	0.00034792	0.003996
sp|Q8BGM7|AAKG3_MOUSE 5'-AMP-activated protein kinase subunit gamma-3 OS=Mus musculus OX=10090 GN=Prkag3 PE=1 SV=1//0	2.3171	8.13E-13	6.25E-11
sp|Q2LKU9|NLR1A_MOUSE NACHT, LRR and PYD domains-containing protein 1a OS=Mus musculus OX=10090 GN=Nlrp1a PE=1 SV=1//0	2.3155	0.00063448	0.0066556
sp|Q80YF6|UPK3B_MOUSE Uroplakin-3b OS=Mus musculus OX=10090 GN=Upk3b PE=1 SV=1//4.67795e-153	2.3105	0.00018781	0.0023832
sp|Q99ME6|AT1B4_MOUSE Protein ATP1B4 OS=Mus musculus OX=10090 GN=Atp1b4 PE=1 SV=1//0	2.3081	4.48E-08	1.41E-06
sp|P22777|PAI1_MOUSE Plasminogen activator inhibitor 1 OS=Mus musculus OX=10090 GN=Serpine1 PE=1 SV=1//0	2.3028	1.19E-17	1.92E-15
sp|Q61703|ITIH2_MOUSE Inter-alpha-trypsin inhibitor heavy chain H2 OS=Mus musculus OX=10090 GN=Itih2 PE=1 SV=1//0	2.2946	0.0059157	0.041664
sp|Q60935|NAR1_MOUSE GPI-linked NAD(P)(+)--arginine ADP-ribosyltransferase 1 OS=Mus musculus OX=10090 GN=Art1 PE=1 SV=1//0	2.2927	4.93E-16	6.47E-14
sp|Q8N9H9|CA127_HUMAN Uncharacterized protein C1orf127 OS=Homo sapiens OX=9606 GN=C1orf127 PE=2 SV=2//2.29202e-77	2.2915	0.00021542	0.0026804
sp|P01592|IGJ_MOUSE Immunoglobulin J chain OS=Mus musculus OX=10090 GN=Jchain PE=1 SV=4//4.60844e-110	2.2792	0.000056335	0.00084177
sp|Q8BXZ0|CTXN3_MOUSE Cortexin-3 OS=Mus musculus OX=10090 GN=Ctxn3 PE=3 SV=1//2.84535e-35	2.2755	4.17E-19	8.81E-17
sp|Q8BHK2|SCN3B_MOUSE Sodium channel subunit beta-3 OS=Mus musculus OX=10090 GN=Scn3b PE=1 SV=1//5.69427e-143	2.2685	0.000033525	0.00053653
sp|Q45HK4|SH21C_MOUSE SH2 domain-containing protein 1B2 OS=Mus musculus OX=10090 GN=Sh2d1b2 PE=1 SV=1//9.99883e-85	2.2609	0.000067383	0.00098508
sp|P70352|NAR5_MOUSE Ecto-ADP-ribosyltransferase 5 OS=Mus musculus OX=10090 GN=Art5 PE=2 SV=3//0	2.258	4.53E-07	0.000011809
sp|Q8BGK2|ARHL1_MOUSE [Protein ADP-ribosylarginine] hydrolase-like protein 1 OS=Mus musculus OX=10090 GN=Adprhl1 PE=1 SV=1//0	2.251	2.04E-17	3.22E-15
sp|O88947|FA10_MOUSE Coagulation factor X OS=Mus musculus OX=10090 GN=F10 PE=1 SV=1//0	2.2492	0.00019244	0.0024331
sp|Q68EF4|GRM4_MOUSE Metabotropic glutamate receptor 4 OS=Mus musculus OX=10090 GN=Grm4 PE=1 SV=2//0	2.2389	0.00059081	0.0062751
sp|P01878|IGHA_MOUSE Ig alpha chain C region OS=Mus musculus OX=10090 PE=1 SV=1//0	2.2382	0.00011181	0.0015225
-//-	2.2273	0.000089461	0.0012638
sp|P97484|LIRB3_MOUSE Leukocyte immunoglobulin-like receptor subfamily B member 3 OS=Mus musculus OX=10090 GN=Lilrb3 PE=1 SV=1//0	2.2237	0.000024343	0.00040588
sp|Q60925|DBP_MOUSE D site-binding protein OS=Mus musculus OX=10090 GN=Dbp PE=1 SV=2//3.37463e-145	2.2212	0.000066835	0.00097777
sp|P10085|MYOD1_MOUSE Myoblast determination protein 1 OS=Mus musculus OX=10090 GN=Myod1 PE=1 SV=2//1.7919e-180	2.2086	1.23E-09	5.43E-08
sp|Q9ES30|C1QT3_MOUSE Complement C1q tumor necrosis factor-related protein 3 OS=Mus musculus OX=10090 GN=C1qtnf3 PE=2 SV=1//3.79584e-130	2.2046	6.02E-15	6.43E-13
-//-	2.1942	0.000081364	0.0011615
sp|Q8BWD2|IP6K3_MOUSE Inositol hexakisphosphate kinase 3 OS=Mus musculus OX=10090 GN=Ip6k3 PE=2 SV=1//0	2.1785	3.18E-15	3.60E-13
sp|O75912|DGKI_HUMAN Diacylglycerol kinase iota OS=Homo sapiens OX=9606 GN=DGKI PE=1 SV=1//0	2.1776	0.00049409	0.0053898
-//-	2.171	0.0055237	0.039474
sp|O88667|RAD_MOUSE GTP-binding protein RAD OS=Mus musculus OX=10090 GN=Rrad PE=1 SV=1//1.33725e-176	2.1682	7.18E-13	5.61E-11
sp|Q3T9E4|TGTP2_MOUSE T-cell-specific guanine nucleotide triphosphate-binding protein 2 OS=Mus musculus OX=10090 GN=Tgtp2 PE=1 SV=2//8.21988e-112	2.1568	0.0041222	0.031026
sp|Q6W3F0|P4HA3_MOUSE Prolyl 4-hydroxylase subunit alpha-3 OS=Mus musculus OX=10090 GN=P4ha3 PE=2 SV=1//0	2.1563	0.00010528	0.0014477
sp|A0A0B4J1G0|FCGR4_MOUSE Low affinity immunoglobulin gamma Fc region receptor IV OS=Mus musculus OX=10090 GN=Fcgr4 PE=1 SV=1//0	2.1521	4.81E-10	2.23E-08
sp|A2BIM8|MUP18_MOUSE Major urinary protein 18 OS=Mus musculus OX=10090 GN=Mup18 PE=3 SV=1//3.03455e-115	2.1518	1.01E-07	3.01E-06
sp|Q80XF5|I22R2_MOUSE Interleukin-22 receptor subunit alpha-2 OS=Mus musculus OX=10090 GN=Il22ra2 PE=1 SV=2//1.88367e-157	2.1495	0.000010962	0.0002014
sp|P51637|CAV3_MOUSE Caveolin-3 OS=Mus musculus OX=10090 GN=Cav3 PE=1 SV=1//1.41212e-104	2.1495	1.79E-08	6.18E-07

sp|P56501|UCP3_MOUSE Mitochondrial uncoupling protein 3 OS=Mus musculus OX=10090 GN=Ucp3 PE=1 SV=1//0	2.1474	8.81E-15	9.18E-13
sp|A2AIP0|F166B_MOUSE Protein FAM166B OS=Mus musculus OX=10090 GN=Fam166b PE=2 SV=1//1.00358e-09	2.144	0.0028181	0.02295
sp|A6NCF5|KLH33_HUMAN Kelch-like protein 33 OS=Homo sapiens OX=9606 GN=KLHL33 PE=4 SV=2//0	2.1335	3.89E-14	3.68E-12
sp|Q9R216|FZD9_MOUSE Frizzled-9 OS=Mus musculus OX=10090 GN=Fzd9 PE=2 SV=1//0	2.1283	3.02E-08	9.85E-07
sp|Q924W6|TRI66_MOUSE Tripartite motif-containing protein 66 OS=Mus musculus OX=10090 GN=Trim66 PE=1 SV=3//0	2.1277	0.0025774	0.021362
sp|Q8BK84|DUPD1_MOUSE Dual specificity phosphatase DUPD1 OS=Mus musculus OX=10090 GN=Dupd1 PE=2 SV=1//1.61351e-158	2.1238	0.0017729	0.015671
sp|Q5SX39|MYH4_MOUSE Myosin-4 OS=Mus musculus OX=10090 GN=Myh4 PE=2 SV=1//0	2.1231	2.80E-10	1.37E-08
-//-	2.1211	0.0011306	0.010777
sp|P33146|CAD15_MOUSE Cadherin-15 OS=Mus musculus OX=10090 GN=Cdh15 PE=1 SV=3//0	2.1157	9.45E-07	0.000022817
-//-	2.11	3.42E-09	1.36E-07
sp|Q8R2Z3|S26A7_MOUSE Anion exchange transporter OS=Mus musculus OX=10090 GN=Slc26a7 PE=2 SV=3//0	2.1062	2.68E-17	4.18E-15
sp|P04187|GRAB_MOUSE Granzyme B(G,H) OS=Mus musculus OX=10090 GN=Gzmb PE=1 SV=1//6.71966e-173	2.1016	0.00030925	0.0036168
sp|P20782|ACHE_MOUSE Acetylcholine receptor subunit epsilon OS=Mus musculus OX=10090 GN=Chrne PE=2 SV=1//0	2.1001	0.00020356	0.0025472
sp|P61374|ISL1_RAT Insulin gene enhancer protein ISL-1 OS=Rattus norvegicus OX=10116 GN=Isl1 PE=1 SV=1//0	2.0973	8.77E-09	3.24E-07
sp|Q6R5N8|TLR13_MOUSE Toll-like receptor 13 OS=Mus musculus OX=10090 GN=Tlr13 PE=1 SV=1//0	2.097	3.01E-12	2.11E-10
sp|Q9QZ85|IIGP1_MOUSE Interferon-inducible GTPase 1 OS=Mus musculus OX=10090 GN=Iigp1 PE=1 SV=2//1.09477e-105	2.0951	2.07E-10	1.04E-08
sp|O95236|APOL3_HUMAN Apolipoprotein L3 OS=Homo sapiens OX=9606 GN=APOL3 PE=1 SV=3//2.57669e-41	2.0926	3.72E-07	9.92E-06
sp|Q80TR4|SLIT1_MOUSE Slit homolog 1 protein OS=Mus musculus OX=10090 GN=Slit1 PE=1 SV=2//3.11839e-36	2.0926	0.00011718	0.0015862
sp|Q9D309|FAM3B_MOUSE Protein FAM3B OS=Mus musculus OX=10090 GN=Fam3b PE=1 SV=1//1.56133e-66	2.0902	0.000021599	0.00036641
sp|Q64345|IFIT3_MOUSE Interferon-induced protein with tetratricopeptide repeats 3 OS=Mus musculus OX=10090 GN=Ifit3 PE=1 SV=1//0	2.0895	0.0032611	0.025781
sp|Q3UQS2|LSME1_MOUSE Leucine-rich single-pass membrane protein 1 OS=Mus musculus OX=10090 GN=Lsmem1 PE=1 SV=1//4.60073e-67	2.0843	5.76E-07	0.000014539
sp|Q9EQ21|HEPC_MOUSE Hepcidin OS=Mus musculus OX=10090 GN=Hamp PE=2 SV=1//1.46704e-41	2.083	0.000042903	0.00066433
sp|Q91WC3|ACSL6_MOUSE Long-chain-fatty-acid--CoA ligase 6 OS=Mus musculus OX=10090 GN=Acsl6 PE=1 SV=1//0	2.0793	2.28E-08	7.66E-07
sp|Q9DCB4|ARP21_MOUSE cAMP-regulated phosphoprotein 21 OS=Mus musculus OX=10090 GN=Arpp21 PE=1 SV=2//1.45099e-131	2.0793	6.42E-11	3.57E-09
-//-	2.07	0.006027	0.042264
sp|Q61321|SIX4_MOUSE Homeobox protein SIX4 OS=Mus musculus OX=10090 GN=Six4 PE=1 SV=1//4.40594e-144	2.0648	1.40E-09	5.99E-08
sp|Q8BLQ0|F124B_MOUSE Protein FAM124B OS=Mus musculus OX=10090 GN=Fam124b PE=1 SV=1//0	2.0627	0.002893	0.023373
sp|Q9EPM5|SYNCI_MOUSE Syncoilin OS=Mus musculus OX=10090 GN=Sync PE=1 SV=1//2.97869e-07	2.0626	6.70E-13	5.29E-11
-//-	2.0568	2.20E-09	9.02E-08
sp|Q3UPR0|SHSA3_MOUSE Protein shisa-3 homolog OS=Mus musculus OX=10090 GN=Shisa3 PE=1 SV=1//3.80449e-126	2.056	0.00042132	0.0046992
sp|O89101|FGF18_MOUSE Fibroblast growth factor 18 OS=Mus musculus OX=10090 GN=Fgf18 PE=2 SV=1//6.81987e-120	2.0528	5.74E-14	5.25E-12
sp|P61016|PPLA_RAT Cardiac phospholamban OS=Rattus norvegicus OX=10116 GN=Pln PE=1 SV=1//6.39359e-07	2.05	0.000054312	0.00081706
sp|P48594|SPB4_HUMAN Serpin B4 OS=Homo sapiens OX=9606 GN=SERPINB4 PE=1 SV=2//4.46435e-138	2.05	3.54E-09	1.40E-07
sp|Q01628|IFM3_HUMAN Interferon-induced transmembrane protein 3 OS=Homo sapiens OX=9606 GN=IFITM3 PE=1 SV=2//2.37946e-33	2.0496	2.79E-08	9.17E-07
sp|Q99JB6|FOXP3_MOUSE Forkhead box protein P3 OS=Mus musculus OX=10090 GN=Foxp3 PE=1 SV=1//0	2.0486	0.0059383	0.041762
sp|P63142|KCNA2_RAT Potassium voltage-gated channel subfamily A member 2 OS=Rattus norvegicus OX=10116 GN=Kcna2 PE=1 SV=1//0	2.048	0.0000329	0.00052966
sp|Q8BG84|LAIR1_MOUSE Leukocyte-associated immunoglobulin-like receptor 1 OS=Mus musculus OX=10090 GN=Lair1 PE=1 SV=1//7.91325e-143	2.0446	1.82E-09	7.64E-08
sp|O70548|TELT_MOUSE Telethonin OS=Mus musculus OX=10090 GN=Tcap PE=1 SV=1//5.08965e-115	2.0429	1.49E-27	1.07E-24
sp|Q8K2F3|DEPP1_MOUSE Protein DEPP1 OS=Mus musculus OX=10090 GN=Depp1 PE=2 SV=1//4.53367e-134	2.0403	1.04E-10	5.55E-09
-//-	2.0374	0.000033476	0.00053611
sp|Q9ESN4|C1QL3_MOUSE Complement C1q-like protein 3 OS=Mus musculus OX=10090 GN=C1ql3 PE=1 SV=1//5.08966e-121	2.0286	0.0032249	0.02557

sp|P97484|LIRB3_MOUSE Leukocyte immunoglobulin-like receptor subfamily B member 3 OS=Mus musculus OX=10090 GN=Lilrb3 PE=1 SV=1//0	2.0146	7.36E-06	0.00014145
sp|Q99JG2|ETBR2_MOUSE Prosaposin receptor GPR37L1 OS=Mus musculus OX=10090 GN=Gpr37l1 PE=1 SV=2//0	2.0126	8.37E-06	0.00015827
sp|Q9Z0V1|KCND3_MOUSE Potassium voltage-gated channel subfamily D member 3 OS=Mus musculus OX=10090 GN=Kcnd3 PE=1 SV=1//0	2.0124	0.00014073	0.0018532
sp|P14231|AT1B2_MOUSE Sodium/potassium-transporting ATPase subunit beta-2 OS=Mus musculus OX=10090 GN=Atp1b2 PE=1 SV=2//4.03922e-50	2.0105	1.29E-09	5.63E-08
sp|O89109|KCNN4_MOUSE Intermediate conductance calcium-activated potassium channel protein 4 OS=Mus musculus OX=10090 GN=Kcnn4 PE=2 SV=1//0	2.0082	0.000737	0.0075488
sp|Q9D783|KLH40_MOUSE Kelch-like protein 40 OS=Mus musculus OX=10090 GN=Klhl40 PE=1 SV=1//0	2.008	1.43E-19	3.25E-17
sp|Q8BXQ3|LRTM1_MOUSE Leucine-rich repeat and transmembrane domain-containing protein 1 OS=Mus musculus OX=10090 GN=Lrtm1 PE=2 SV=1//0	2.0059	1.47E-08	5.14E-07
sp|Q9R0W1|PITX2_RAT Pituitary homeobox 2 OS=Rattus norvegicus OX=10116 GN=Pitx2 PE=2 SV=1//4.39484e-34	2.0021	1.12E-10	5.95E-09
sp|P27573|MYP0_MOUSE Myelin protein P0 OS=Mus musculus OX=10090 GN=Mpz PE=1 SV=1//4.87732e-119	2.0005	2.16E-24	1.01E-21
**Blast swiss prot (**; down>2-FC 320 genes)	**log2 FoldChange**	**pval**	**padj**
sp|Q8VCW2|K1C25_MOUSE Keratin, type I cytoskeletal 25 OS=Mus musculus OX=10090 GN=Krt25 PE=1 SV=1//0	-14.357	1.40E-13	1.24E-11
sp|Q9QZU5|KR151_MOUSE Keratin-associated protein 15-1 OS=Mus musculus OX=10090 GN=Krtap15-1 PE=2 SV=1//1.22776e-70	-13.753	0.0032498	0.025717
sp|Q6IME9|K2C72_MOUSE Keratin, type II cytoskeletal 72 OS=Mus musculus OX=10090 GN=Krt72 PE=3 SV=1//0	-13.656	1.50E-07	4.32E-06
sp|Q9D3P1|TCHL1_MOUSE Trichohyalin-like protein 1 OS=Mus musculus OX=10090 GN=Tchhl1 PE=2 SV=2//0	-13.612	0.00002507	0.00041636
sp|Q9Z320|K1C27_MOUSE Keratin, type I cytoskeletal 27 OS=Mus musculus OX=10090 GN=Krt27 PE=1 SV=1//0	-13.409	2.47E-09	1.00E-07
sp|Q9R0H5|K2C71_MOUSE Keratin, type II cytoskeletal 71 OS=Mus musculus OX=10090 GN=Krt71 PE=1 SV=1//0	-13.289	5.43E-11	3.08E-09
sp|Q8CE93|CAHM4_MOUSE Calcium homeostasis modulator protein 4 OS=Mus musculus OX=10090 GN=Calhm4 PE=2 SV=2//0	-12.387	0.000078367	0.001124
sp|Q6NXH9|K2C73_MOUSE Keratin, type II cytoskeletal 73 OS=Mus musculus OX=10090 GN=Krt73 PE=1 SV=1//0	-12.326	4.60E-26	2.67E-23
-//-	-11.724	0.00077844	0.0078705
sp|O08640|KRA14_MOUSE Keratin-associated protein 14 OS=Mus musculus OX=10090 GN=Krtap14 PE=2 SV=1//4.50373e-93	-11.615	0.0060099	0.042156
sp|Q8IUC1|KR111_HUMAN Keratin-associated protein 11-1 OS=Homo sapiens OX=9606 GN=KRTAP11-1 PE=2 SV=1//3.23853e-81	-11.592	0.00072658	0.0074577
sp|Q3V1I0|LYG2_MOUSE Lysozyme g-like protein 2 OS=Mus musculus OX=10090 GN=Lyg2 PE=2 SV=1//1.40869e-150	-11.48	0.000001274	0.000029894
sp|Q9BYQ7|KRA41_HUMAN Keratin-associated protein 4-1 OS=Homo sapiens OX=9606 GN=KRTAP4-1 PE=2 SV=3//1.04713e-07	-11.155	0.000060102	0.00089149
sp|Q3TRJ4|K1C26_MOUSE Keratin, type I cytoskeletal 26 OS=Mus musculus OX=10090 GN=Krt26 PE=2 SV=1//5.05234e-88	-11.151	2.82E-07	7.73E-06
sp|Q6IMF0|KRT87_MOUSE Keratin, type II cuticular 87 OS=Mus musculus OX=10090 GN=Krt87 PE=2 SV=2//0	-10.713	0.00016813	0.0021651
sp|Q8K0Y2|KT33A_MOUSE Keratin, type I cuticular Ha3-I OS=Mus musculus OX=10090 GN=Krt33a PE=1 SV=1//0	-10.667	0.0015199	0.013765
sp|Q6P3A4|VSIG8_MOUSE V-set and immunoglobulin domain-containing protein 8 OS=Mus musculus OX=10090 GN=Vsig8 PE=2 SV=2//0	-10.591	0.00013968	0.0018446
sp|Q9BYT5|KRA22_HUMAN Keratin-associated protein 2-2 OS=Homo sapiens OX=9606 GN=KRTAP2-2 PE=2 SV=3//8.64259e-17	-10.577	0.0040272	0.030472
sp|Q497I4|KRT35_MOUSE Keratin, type I cuticular Ha5 OS=Mus musculus OX=10090 GN=Krt35 PE=1 SV=1//2.69816e-73	-10.47	9.72E-15	9.97E-13
sp|Q91VA3|CAN8_MOUSE Calpain-8 OS=Mus musculus OX=10090 GN=Capn8 PE=1 SV=1//0	-10.335	1.38E-09	5.95E-08
sp|O95932|TGM3L_HUMAN Protein-glutamine gamma-glutamyltransferase 6 OS=Homo sapiens OX=9606 GN=TGM6 PE=1 SV=3//0	-10.292	9.47E-06	0.00017687
sp|Q9ERE2|KRT81_MOUSE Keratin, type II cuticular Hb1 OS=Mus musculus OX=10090 GN=Krt81 PE=2 SV=2//0	-10.24	0.0032557	0.025747
-//-	-9.9895	0.0027083	0.02225
sp|Q9UBG3|CRNN_HUMAN Cornulin OS=Homo sapiens OX=9606 GN=CRNN PE=1 SV=1//5.50769e-138	-9.9056	0.000019257	0.00033317
sp|Q9Z185|PADI1_MOUSE Protein-arginine deiminase type-1 OS=Mus musculus OX=10090 GN=Padi1 PE=1 SV=1//0	-9.833	0.00015645	0.0020298
sp|Q61765|K1H1_MOUSE Keratin, type I cuticular Ha1 OS=Mus musculus OX=10090 GN=Krt31 PE=1 SV=2//0	-9.7315	0.0015579	0.014073
sp|Q9Z184|PADI3_MOUSE Protein-arginine deiminase type-3 OS=Mus musculus OX=10090 GN=Padi3 PE=1 SV=2//0	-9.5813	1.67E-06	0.000037812
sp|A2A5X5|KR161_MOUSE Keratin-associated protein 16-1 OS=Mus musculus OX=10090 GN=Krtap16-1 PE=3 SV=1//0	-9.5122	0.00086557	0.0085871
sp|Q9D638|KRA32_MOUSE Keratin-associated protein 3-2 OS=Mus musculus OX=10090 GN=Krtap3-2 PE=3 SV=2//3.78182e-40	-9.2222	0.0031615	0.025182
sp|P22793|TRHY_SHEEP Trichohyalin OS=Ovis aries OX=9940 GN=TCHH PE=2 SV=2//7.5095e-08	-9.1875	0.000025817	0.00042723

-//-	-9.0451	0.0019169	0.016696
sp|P23299|KCNE1_MOUSE Potassium voltage-gated channel subfamily E member 1 OS=Mus musculus OX=10090 GN=Kcne1 PE=1 SV=1//3.50984e-82	-8.8584	0.0040558	0.03065
sp|Q8BV84|PRR9_MOUSE Proline-rich protein 9 OS=Mus musculus OX=10090 GN=Prr9 PE=4 SV=1//2.47278e-42	-8.833	6.14E-06	0.000121
sp|Q6IFX3|K1C40_MOUSE Keratin, type I cytoskeletal 40 OS=Mus musculus OX=10090 GN=Krt40 PE=2 SV=1//0	-8.6055	0.00025912	0.0031267
sp|P62819|S10A3_RAT Protein S100-A3 OS=Rattus norvegicus OX=10116 GN=S100a3 PE=1 SV=1//2.22897e-52	-8.5534	0.000037155	0.00058614
sp|Q7TMD7|DSG4_MOUSE Desmoglein-4 OS=Mus musculus OX=10090 GN=Dsg4 PE=1 SV=1//0	-8.1844	0.00019562	0.0024681
sp|Q8C196|CPSM_MOUSE Carbamoyl-phosphate synthase [ammonia], mitochondrial OS=Mus musculus OX=10090 GN=Cps1 PE=1 SV=2//0	-8.1286	3.38E-32	3.92E-29
sp|Q9QXD6|F16P1_MOUSE Fructose-1,6-bisphosphatase 1 OS=Mus musculus OX=10090 GN=Fbp1 PE=1 SV=3//0	-7.6898	2.52E-10	1.25E-08
sp|Q99M74|KRT82_MOUSE Keratin, type II cuticular Hb2 OS=Mus musculus OX=10090 GN=Krt82 PE=1 SV=2//0	-7.6801	0.0016892	0.015046
sp|Q62226|SHH_MOUSE Sonic hedgehog protein OS=Mus musculus OX=10090 GN=Shh PE=1 SV=2//0	-7.6768	3.29E-13	2.76E-11
sp|Q60928|GGT1_MOUSE Glutathione hydrolase 1 proenzyme OS=Mus musculus OX=10090 GN=Ggt1 PE=1 SV=1//0	-7.5946	2.62E-16	3.59E-14
sp|Q62052|P_MOUSE P protein OS=Mus musculus OX=10090 GN=Oca2 PE=1 SV=1//1.01015e-58	-7.5631	2.24E-07	6.22E-06
sp|B1AQ75|KRT36_MOUSE Keratin, type I cuticular Ha6 OS=Mus musculus OX=10090 GN=Krt36 PE=1 SV=1//0	-7.4611	0.000032565	0.00052532
sp|Q8R2I0|FOXE1_MOUSE Forkhead box protein E1 OS=Mus musculus OX=10090 GN=Foxe1 PE=1 SV=2//5.24568e-96	-7.0332	7.65E-88	1.86E-83
sp|Q925E8|SPTSB_MOUSE Serine palmitoyltransferase small subunit B OS=Mus musculus OX=10090 GN=Sptssb PE=2 SV=1//6.69417e-45	-6.9555	0.0045598	0.033848
sp|Q6IFZ9|K2C74_MOUSE Keratin, type II cytoskeletal 74 OS=Mus musculus OX=10090 GN=Krt74 PE=3 SV=1//0	-6.9235	1.89E-06	0.000042137
sp|Q9Z1Q3|LY66D_MOUSE Lymphocyte antigen 6 complex locus protein G6d OS=Mus musculus OX=10090 GN=Ly6g6d PE=1 SV=1//2.02633e-70	-6.9027	0.0029516	0.023745
sp|A6BLY7|K1C28_MOUSE Keratin, type I cytoskeletal 28 OS=Mus musculus OX=10090 GN=Krt28 PE=1 SV=1//0	-6.9023	5.26E-14	4.88E-12
sp|Q9JJV0|CRBA4_MOUSE Beta-crystallin A4 OS=Mus musculus OX=10090 GN=Cryba4 PE=2 SV=3//6.04292e-144	-6.8597	0.00010277	0.0014212
sp|Q00977|CXB2_MOUSE Gap junction beta-2 protein OS=Mus musculus OX=10090 GN=Gjb2 PE=1 SV=1//1.8232e-161	-6.8149	1.74E-08	6.03E-07
-//-	-6.7618	0.0013408	0.012407
sp|P29391|FRIL1_MOUSE Ferritin light chain 1 OS=Mus musculus OX=10090 GN=Ftl1 PE=1 SV=2//3.38699e-117	-6.6076	0.0057642	0.040809
sp|Q8WY07|CTR3_HUMAN Cationic amino acid transporter 3 OS=Homo sapiens OX=9606 GN=SLC7A3 PE=1 SV=1//0	-6.5125	0.00013326	0.0017685
sp|P26804|ENV_MLVFF Envelope glycoprotein OS=Friend murine leukemia virus (isolate FB29) OX=11797 GN=env PE=1 SV=1//2.72293e-109	-6.3716	3.17E-54	1.10E-50
sp|Q08535|SECR_MOUSE Secretin OS=Mus musculus OX=10090 GN=Sct PE=2 SV=1//1.21543e-60	-6.349	0.0014538	0.013285
sp|Q6S5I3|S115A_MOUSE Protein S100-A15A OS=Mus musculus OX=10090 GN=S100a15a PE=3 SV=1//2.16594e-63	-6.243	0.00017802	0.0022795
sp|Q01727|MSHR_MOUSE Melanocyte-stimulating hormone receptor OS=Mus musculus OX=10090 GN=Mc1r PE=2 SV=2//8.10263e-180	-6.086	1.53E-20	4.23E-18
sp|P58355|S45A2_MOUSE Membrane-associated transporter protein OS=Mus musculus OX=10090 GN=Slc45a2 PE=1 SV=1//0	-6.0713	1.32E-06	0.000030797
sp|P35347|CRFR1_MOUSE Corticotropin-releasing factor receptor 1 OS=Mus musculus OX=10090 GN=Crhr1 PE=1 SV=1//0	-6.0673	1.08E-19	2.53E-17
sp|Q64448|CXA3_MOUSE Gap junction alpha-3 protein OS=Mus musculus OX=10090 GN=Gja3 PE=2 SV=4//0	-6.0434	0.0072051	0.048498
sp|P04919|B3AT_MOUSE Band 3 anion transport protein OS=Mus musculus OX=10090 GN=Slc4a1 PE=1 SV=1//0	-6.0215	0.0040698	0.030689
sp|Q9CQD7|PINLY_MOUSE phospholipase A2 inhibitor and Ly6/PLAUR domain-containing protein OS=Mus musculus OX=10090 GN=Pinlyp PE=2 SV=1//1.18113e-136	-6.0003	0.00093577	0.0091752
sp|P70269|CATE_MOUSE Cathepsin E OS=Mus musculus OX=10090 GN=Ctse PE=1 SV=2//0	-5.9969	0.000029891	0.00048767
sp|Q61282|PGCA_MOUSE Aggrecan core protein OS=Mus musculus OX=10090 GN=Acan PE=1 SV=2//0	-5.9252	0.00037359	0.0042408
sp|Q64317|DLX1_MOUSE Homeobox protein DLX-1 OS=Mus musculus OX=10090 GN=Dlx1 PE=1 SV=1//5.05544e-132	-5.9084	3.36E-34	4.54E-31
sp|P70436|DLX4_MOUSE Homeobox protein DLX-4 OS=Mus musculus OX=10090 GN=Dlx4 PE=2 SV=2//1.00402e-131	-5.9069	2.76E-20	7.06E-18
sp|Q9QXF8|GNMT_MOUSE Glycine N-methyltransferase OS=Mus musculus OX=10090 GN=Gnmt PE=1 SV=3//0	-5.905	1.99E-66	2.42E-62
sp|Q4VC17|ATS18_MOUSE A disintegrin and metalloproteinase with thrombospondin motifs 18 OS=Mus musculus OX=10090 GN=Adamts18 PE=2 SV=2//0	-5.8677	1.61E-54	6.53E-51
sp|P40749|SYT4_MOUSE Synaptotagmin-4 OS=Mus musculus OX=10090 GN=Syt4 PE=1 SV=2//0	-5.8303	4.02E-10	1.89E-08
sp|Q9R1Q8|TAGL3_MOUSE Transgelin-3 OS=Mus musculus OX=10090 GN=Tagln3 PE=1 SV=1//1.04828e-144	-5.8033	0.00083888	0.0083668
-//-	-5.701	0.00075257	0.0076535

sp|P11344|TYRO_MOUSE Tyrosinase OS=Mus musculus OX=10090 GN=Tyr PE=1 SV=3//0	-5.6083	8.45E-10	3.82E-08
sp|Q16655|MAR1_HUMAN Melanoma antigen recognized by T-cells 1 OS=Homo sapiens OX=9606 GN=MLANA PE=1 SV=1//8.6264e-32	-5.5279	0.000016298	0.00028709
sp|Q2TV84|TRPM1_MOUSE Transient receptor potential cation channel subfamily M member 1 OS=Mus musculus OX=10090 GN=Trpm1 PE=2 SV=2//0	-5.5188	0.000022725	0.00038189
sp|Q61474|MSI1H_MOUSE RNA-binding protein Musashi homolog 1 OS=Mus musculus OX=10090 GN=Msi1 PE=1 SV=1//0	-5.4428	4.28E-07	0.000011253
-//-	-5.3801	0.00010319	0.0014255
sp|P70259|GP143_MOUSE G-protein coupled receptor 143 OS=Mus musculus OX=10090 GN=Gpr143 PE=2 SV=1//0	-5.3488	0.000011091	0.00020332
sp|Q8CGQ8|NCKX4_MOUSE Sodium/potassium/calcium exchanger 4 OS=Mus musculus OX=10090 GN=Slc24a4 PE=1 SV=2//4.60323e-23	-5.2599	6.31E-26	3.57E-23
sp|P40764|DLX2_MOUSE Homeobox protein DLX-2 OS=Mus musculus OX=10090 GN=Dlx2 PE=1 SV=1//6.67834e-160	-5.2334	6.24E-53	1.90E-49
sp|Q8K3F7|TDH_MOUSE L-threonine 3-dehydrogenase, mitochondrial OS=Mus musculus OX=10090 GN=Tdh PE=1 SV=1//0	-5.1495	1.49E-09	6.35E-08
sp|P25688|URIC_MOUSE Uricase OS=Mus musculus OX=10090 GN=Uox PE=1 SV=2//0	-5.0614	0.0037178	0.028557
sp|O54792|HES2_MOUSE Transcription factor HES-2 OS=Mus musculus OX=10090 GN=Hes2 PE=2 SV=2//5.59769e-72	-5.0397	7.34E-22	2.55E-19
-//-	-4.9851	0.00069359	0.0071736
sp|O54983|CRYM_MOUSE Ketimine reductase mu-crystallin OS=Mus musculus OX=10090 GN=Crym PE=1 SV=1//1.3428e-15	-4.9437	0.0027367	0.022432
sp|P31387|5HT5B_MOUSE 5-hydroxytryptamine receptor 5B OS=Mus musculus OX=10090 GN=Htr5b PE=2 SV=1//0	-4.87	0.0020985	0.018013
sp|P34821|BMP8A_MOUSE Bone morphogenetic protein 8A OS=Mus musculus OX=10090 GN=Bmp8a PE=2 SV=1//0	-4.8647	0.00011421	0.0015496
sp|Q7TPD7|LR75B_MOUSE Leucine-rich repeat-containing protein 75B OS=Mus musculus OX=10090 GN=Lrrc75b PE=2 SV=1//4.51971e-140	-4.8344	1.52E-06	0.000034734
sp|Q8R197|SAST_MOUSE S-acyl fatty acid synthase thioesterase, medium chain OS=Mus musculus OX=10090 GN=Olah PE=2 SV=1//0	-4.791	0.00214	0.018279
sp|Q03358|MSX2_MOUSE Homeobox protein MSX-2 OS=Mus musculus OX=10090 GN=Msx2 PE=1 SV=2//7.97144e-166	-4.7419	8.08E-07	0.000019767
sp|Q8C261|NCKX5_MOUSE Sodium/potassium/calcium exchanger 5 OS=Mus musculus OX=10090 GN=Slc24a5 PE=2 SV=1//0	-4.7009	5.38E-07	0.000013702
-//-	-4.6728	0.0046134	0.034132
sp|Q8VCF5|TSN10_MOUSE Tetraspanin-10 OS=Mus musculus OX=10090 GN=Tspan10 PE=1 SV=1//0	-4.6448	4.78E-19	9.92E-17
sp|Q80YC5|FA12_MOUSE Coagulation factor XII OS=Mus musculus OX=10090 GN=F12 PE=1 SV=2//0	-4.6361	0.0054449	0.03906
sp|Q8BXA6|CLD17_MOUSE Claudin-17 OS=Mus musculus OX=10090 GN=Cldn17 PE=1 SV=1//4.39299e-119	-4.6116	0.0061444	0.04284
sp|Q80VJ8|KASH5_MOUSE Protein KASH5 OS=Mus musculus OX=10090 GN=Ccdc155 PE=1 SV=3//0	-4.5878	2.31E-15	2.71E-13
sp|P13297|MSX1_MOUSE Homeobox protein MSX-1 OS=Mus musculus OX=10090 GN=Msx1 PE=1 SV=4//1.01829e-138	-4.5773	1.31E-62	1.06E-58
sp|Q9DBB9|CPN2_MOUSE Carboxypeptidase N subunit 2 OS=Mus musculus OX=10090 GN=Cpn2 PE=1 SV=2//0	-4.4022	0.00024523	0.0029872
sp|Q9D428|GOG7B_MOUSE Golgin subfamily A member 7B OS=Mus musculus OX=10090 GN=GOLGA7B PE=1 SV=1//3.98989e-93	-4.3949	6.03E-37	1.22E-33
sp|P07147|TYRP1_MOUSE 5,6-dihydroxyindole-2-carboxylic acid oxidase OS=Mus musculus OX=10090 GN=Tyrp1 PE=1 SV=1//0	-4.3886	0.000013539	0.00024307
sp|P70689|CXB6_MOUSE Gap junction beta-6 protein OS=Mus musculus OX=10090 GN=Gjb6 PE=1 SV=1//0	-4.3575	0.00027519	0.0032847
sp|Q8CCC3|CL056_MOUSE Uncharacterized protein C12orf56 homolog OS=Mus musculus OX=10090 PE=2 SV=1//0	-4.3242	0.0051353	0.037212
sp|Q60696|PMEL_MOUSE Melanocyte protein PMEL OS=Mus musculus OX=10090 GN=Pmel PE=1 SV=1//0	-4.2918	1.19E-11	7.71E-10
sp|P03966|MYCN_MOUSE N-myc proto-oncogene protein OS=Mus musculus OX=10090 GN=Mycn PE=2 SV=2//0	-4.2764	3.06E-59	1.86E-55
sp|Q9UKR3|KLK13_HUMAN Kallikrein-13 OS=Homo sapiens OX=9606 GN=KLK13 PE=2 SV=1//1.80094e-157	-4.2429	4.84E-22	1.73E-19
sp|A2AFS3|K1324_MOUSE UPF0577 protein KIAA1324 OS=Mus musculus OX=10090 GN=Kiaa1324 PE=1 SV=1//0	-4.1787	1.00E-06	0.000024099
sp|P70436|DLX4_MOUSE Homeobox protein DLX-4 OS=Mus musculus OX=10090 GN=Dlx4 PE=2 SV=2//2.33109e-07	-4.1559	1.65E-15	2.01E-13
sp|P50207|HXC13_MOUSE Homeobox protein Hox-C13 OS=Mus musculus OX=10090 GN=Hoxc13 PE=2 SV=2//1.78203e-167	-4.1461	0.000055029	0.0008258
sp|Q8C432|RN182_MOUSE E3 ubiquitin-protein ligase RNF182 OS=Mus musculus OX=10090 GN=Rnf182 PE=2 SV=1//9.3261e-127	-4.1459	0.0054759	0.039195
sp|Q8CBF3|EPHB1_MOUSE Ephrin type-B receptor 1 OS=Mus musculus OX=10090 GN=Ephb1 PE=1 SV=1//0	-4.1118	0.000024777	0.00041226
sp|Q64205|DLX3_MOUSE Homeobox protein DLX-3 OS=Mus musculus OX=10090 GN=Dlx3 PE=2 SV=1//2.60838e-166	-4.055	2.00E-09	8.27E-08
sp|Q15041|AR6P1_HUMAN ADP-ribosylation factor-like protein 6-interacting protein 1 OS=Homo sapiens OX=9606 GN=ARL6IP1 PE=1 SV=2//1.78981e-08	-4.0477	8.63E-06	0.00016295
sp|P30875|SSR2_MOUSE Somatostatin receptor type 2 OS=Mus musculus OX=10090 GN=Sstr2 PE=2 SV=1//0	-4.0331	5.30E-07	0.000013536

sp|Q9Z2C6|UPK1B_MOUSE Uroplakin-1b OS=Mus musculus OX=10090 GN=Upk1b PE=2 SV=3//0	-4.0044	6.09E-09	2.32E-07
sp|Q9Z319|CORIN_MOUSE Atrial natriuretic peptide-converting enzyme OS=Mus musculus OX=10090 GN=Corin PE=2 SV=2//0	-3.9379	4.42E-36	8.27E-33
sp|P22389|EDN2_MOUSE Endothelin-2 OS=Mus musculus OX=10090 GN=Edn2 PE=2 SV=3//5.92089e-93	-3.9323	1.61E-23	7.26E-21
sp|Q6ZUA9|MROH5_HUMAN Maestro heat-like repeat family member 5 OS=Homo sapiens OX=9606 GN=MROH5 PE=2 SV=2//2.27737e-117	-3.9292	0.00094701	0.0092779
sp|Q8QZW7|GBRP_MOUSE Gamma-aminobutyric acid receptor subunit pi OS=Mus musculus OX=10090 GN=Gabrp PE=2 SV=1//0	-3.9094	0.0015989	0.014395
sp|P27782|LEF1_MOUSE Lymphoid enhancer-binding factor 1 OS=Mus musculus OX=10090 GN=Lef1 PE=1 SV=1//1.05632e-63	-3.854	1.64E-23	7.26E-21
sp|O88507|CNTFR_MOUSE Ciliary neurotrophic factor receptor subunit alpha OS=Mus musculus OX=10090 GN=Cntfr PE=1 SV=2//0	-3.8414	6.85E-57	3.33E-53
sp|O35084|CP27B_MOUSE 25-hydroxyvitamin D-1 alpha hydroxylase, mitochondrial OS=Mus musculus OX=10090 GN=Cyp27b1 PE=1 SV=2//5.04512e-31	-3.8327	5.42E-15	5.81E-13
sp|Q61245|COBA1_MOUSE Collagen alpha-1(XI) chain OS=Mus musculus OX=10090 GN=Col11a1 PE=1 SV=2//3.1415e-06	-3.8261	1.26E-15	1.54E-13
sp|Q9JI67|B3GT5_MOUSE Beta-1,3-galactosyltransferase 5 OS=Mus musculus OX=10090 GN=B3galt5 PE=2 SV=1//0	-3.8062	4.25E-09	1.65E-07
sp|Q9D7D2|SPA9_MOUSE Serpin A9 OS=Mus musculus OX=10090 GN=Serpina9 PE=2 SV=1//0	-3.7875	4.92E-28	3.74E-25
sp|P97326|CADH6_MOUSE Cadherin-6 OS=Mus musculus OX=10090 GN=Cdh6 PE=1 SV=2//0	-3.7223	0.0024807	0.020671
sp|A2ARV4|LRP2_MOUSE Low-density lipoprotein receptor-related protein 2 OS=Mus musculus OX=10090 GN=Lrp2 PE=1 SV=1//0	-3.7074	0.000021945	0.00037045
sp|Q8BWQ5|DCLK3_MOUSE Serine/threonine-protein kinase DCLK3 OS=Mus musculus OX=10090 GN=Dclk3 PE=1 SV=2//0	-3.6943	5.93E-06	0.00011742
sp|Q80YY7|ZN618_MOUSE Zinc finger protein 618 OS=Mus musculus OX=10090 GN=Znf618 PE=2 SV=3//0	-3.691	0.00032682	0.0037949
sp|Q6DFY8|BRNP2_MOUSE BMP/retinoic acid-inducible neural-specific protein 2 OS=Mus musculus OX=10090 GN=Brinp2 PE=1 SV=1//0	-3.6465	6.99E-06	0.00013496
sp|Q99PN0|SC5A5_MOUSE Sodium/iodide cotransporter OS=Mus musculus OX=10090 GN=Slc5a5 PE=2 SV=1//0	-3.6332	0.0021454	0.018318
sp|O88866|HUNK_MOUSE Hormonally up-regulated neu tumor-associated kinase OS=Mus musculus OX=10090 GN=Hunk PE=2 SV=1//0	-3.6017	0.00017888	0.0022868
sp|P29812|TYRP2_MOUSE L-dopachrome tautomerase OS=Mus musculus OX=10090 GN=Dct PE=1 SV=2//0	-3.5716	5.18E-11	2.95E-09
sp|Q9JJV4|CCG4_MOUSE Voltage-dependent calcium channel gamma-4 subunit OS=Mus musculus OX=10090 GN=Cacng4 PE=1 SV=1//0	-3.5708	0.0027444	0.022478
sp|Q14B46|RTKN2_MOUSE Rhotekin-2 OS=Mus musculus OX=10090 GN=Rtkn2 PE=1 SV=2//0	-3.5629	5.37E-07	0.000013682
sp|Q8BFU0|RSPO2_MOUSE R-spondin-2 OS=Mus musculus OX=10090 GN=Rspo2 PE=1 SV=1//7.74758e-153	-3.5494	3.76E-11	2.23E-09
sp|Q8VI67|SP7_MOUSE Transcription factor Sp7 OS=Mus musculus OX=10090 GN=Sp7 PE=1 SV=1//0	-3.5273	0.00014477	0.0018963
sp|Q8BVG5|GLT14_MOUSE Polypeptide N-acetylgalactosaminyltransferase 14 OS=Mus musculus OX=10090 GN=Galnt14 PE=2 SV=2//0	-3.5197	5.24E-11	2.98E-09
sp|O95678|K2C75_HUMAN Keratin, type II cytoskeletal 75 OS=Homo sapiens OX=9606 GN=KRT75 PE=1 SV=2//0	-3.4925	4.71E-11	2.72E-09
sp|Q9D0L6|BAMBI_MOUSE BMP and activin membrane-bound inhibitor homolog OS=Mus musculus OX=10090 GN=Bambi PE=2 SV=1//4.47048e-163	-3.4741	0.000010484	0.00019378
sp|P15656|FGF5_MOUSE Fibroblast growth factor 5 OS=Mus musculus OX=10090 GN=Fgf5 PE=2 SV=1//1.13065e-136	-3.4627	0.000018308	0.00031902
sp|Q8BLR2|CPNE4_MOUSE Copine-4 OS=Mus musculus OX=10090 GN=Cpne4 PE=1 SV=1//0	-3.4575	1.72E-07	4.91E-06
sp|Q60HC2|TBB3_MACFA Tubulin beta-3 chain OS=Macaca fascicularis OX=9541 GN=TUBB3 PE=2 SV=1//0	-3.4565	0.00001961	0.00033833
sp|Q69ZT9|TBC30_MOUSE TBC1 domain family member 30 OS=Mus musculus OX=10090 GN=Tbc1d30 PE=1 SV=2//0	-3.4503	5.66E-08	1.77E-06
sp|P59509|ATS19_MOUSE A disintegrin and metalloproteinase with thrombospondin motifs 19 OS=Mus musculus OX=10090 GN=Adamts19 PE=2 SV=1//0	-3.4038	2.99E-09	1.20E-07
sp|P52624|UPP1_MOUSE Uridine phosphorylase 1 OS=Mus musculus OX=10090 GN=Upp1 PE=1 SV=2//0	-3.3932	0.0031056	0.02481
sp|Q80X72|LRC15_MOUSE Leucine-rich repeat-containing protein 15 OS=Mus musculus OX=10090 GN=Lrrc15 PE=2 SV=1//0	-3.3631	0.00076466	0.0077634
sp|Q8BHB9|CLIC6_MOUSE Chloride intracellular channel protein 6 OS=Mus musculus OX=10090 GN=Clic6 PE=1 SV=1//0	-3.3574	6.76E-06	0.00013133
sp|Q9YGP6|SLBP2_XENLA Oocyte-specific histone RNA stem-loop-binding protein 2 OS=Xenopus laevis OX=8355 GN=slbp2 PE=2 SV=1//6.75884e-10	-3.3412	0.0022093	0.018755
-//-	-3.2725	0.003391	0.026584
sp|Q6P5F6|S39AA_MOUSE Zinc transporter ZIP10 OS=Mus musculus OX=10090 GN=Slc39a10 PE=1 SV=1//0	-3.2099	2.37E-15	2.74E-13
sp|Q9JHW9|AL1A3_MOUSE Aldehyde dehydrogenase family 1 member A3 OS=Mus musculus OX=10090 GN=Aldh1a3 PE=1 SV=1//0	-3.1561	3.29E-06	0.000069267
sp|Q9QXW9|LAT2_MOUSE Large neutral amino acids transporter small subunit 2 OS=Mus musculus OX=10090 GN=Slc7a8 PE=1 SV=1//0	-3.1423	0.00051603	0.0055891
sp|Q9CWR7|STEA1_MOUSE Metalloreductase STEAP1 OS=Mus musculus OX=10090 GN=Steap1 PE=1 SV=2//0	-3.1065	6.91E-07	0.000017167
sp|O35595|PTC2_MOUSE Protein patched homolog 2 OS=Mus musculus OX=10090 GN=Ptch2 PE=2 SV=2//0	-3.0818	2.42E-09	9.83E-08

sp|Q7TSC3|NEK5_MOUSE Serine/threonine-protein kinase Nek5 OS=Mus musculus OX=10090 GN=Nek5 PE=2 SV=1//6.21185e-21	-3.0768	0.00030562	0.0035846
sp|Q76K27|SIAT2_MOUSE Beta-galactoside alpha-2,6-sialyltransferase 2 OS=Mus musculus OX=10090 GN=St6gal2 PE=2 SV=2//0	-3.0764	0.0010211	0.0098603
sp|Q8K097|LFG2_MOUSE Protein lifeguard 2 OS=Mus musculus OX=10090 GN=Faim2 PE=2 SV=1//0	-3.0305	0.0004158	0.0046462
-//-	-3.0168	0.00012349	0.0016588
sp|Q9CYS6|CB072_MOUSE Uncharacterized protein C2orf72 homolog OS=Mus musculus OX=10090 PE=1 SV=2//4.9327e-150	-3.0076	0.0013102	0.012172
sp|P40753|ANFB_MOUSE Natriuretic peptides B OS=Mus musculus OX=10090 GN=Nppb PE=2 SV=2//4.77485e-62	-2.9989	0.0024665	0.020583
-//-	-2.9978	0.0014549	0.01329
sp|Q6T4R5|NHS_HUMAN Nance-Horan syndrome protein OS=Homo sapiens OX=9606 GN=NHS PE=1 SV=2//0	-2.98	2.41E-31	2.55E-28
sp|O08969|PHLA2_MOUSE Pleckstrin homology-like domain family A member 2 OS=Mus musculus OX=10090 GN=Phlda2 PE=1 SV=1//1.38545e-102	-2.9694	0.00083512	0.0083419
sp|Q66PY1|SCUB3_MOUSE Signal peptide, CUB and EGF-like domain-containing protein 3 OS=Mus musculus OX=10090 GN=Scube3 PE=1 SV=1//0	-2.9693	2.33E-20	6.02E-18
sp|Q9EQG5|BEAN1_MOUSE Protein BEAN1 OS=Mus musculus OX=10090 GN=Bean1 PE=1 SV=2//6.73873e-116	-2.9682	0.0047966	0.035262
sp|Q8BS03|PI15_MOUSE Peptidase inhibitor 15 OS=Mus musculus OX=10090 GN=Pi15 PE=2 SV=2//6.17885e-167	-2.9551	7.09E-48	1.92E-44
-//-	-2.9546	0.0048018	0.03529
sp|Q63170|DYH7_RAT Dynein heavy chain 7, axonemal OS=Rattus norvegicus OX=10116 GN=Dnah7 PE=2 SV=2//0	-2.9402	5.34E-06	0.00010683
sp|Q6XD76|ASCL4_HUMAN Achaete-scute homolog 4 OS=Homo sapiens OX=9606 GN=ASCL4 PE=1 SV=1//3.78484e-31	-2.938	0.000024412	0.00040674
sp|P06876|MYB_MOUSE Transcriptional activator Myb OS=Mus musculus OX=10090 GN=Myb PE=1 SV=2//1.94915e-152	-2.9363	7.39E-21	2.30E-18
sp|A1L020|MEX3A_HUMAN RNA-binding protein MEX3A OS=Homo sapiens OX=9606 GN=MEX3A PE=1 SV=1//0	-2.9344	4.12E-33	5.01E-30
sp|Q3TY65|ICA1L_MOUSE Islet cell autoantigen 1-like protein OS=Mus musculus OX=10090 GN=Ica1l PE=1 SV=1//0	-2.9145	0.00092156	0.0090577
sp|P23242|CXA1_MOUSE Gap junction alpha-1 protein OS=Mus musculus OX=10090 GN=Gja1 PE=1 SV=2//0	-2.8636	0.00026518	0.0031887
sp|Q9DA75|NLS1_MOUSE Sodium-dependent lysophosphatidylcholine symporter 1 OS=Mus musculus OX=10090 GN=Mfsd2a PE=1 SV=1//0	-2.8564	4.19E-26	2.49E-23
sp|P48302|EDNRB_MOUSE Endothelin receptor type B OS=Mus musculus OX=10090 GN=Ednrb PE=2 SV=1//0	-2.8391	5.31E-32	5.87E-29
sp|E9Q6Z5|AUNIP_MOUSE Aurora kinase A and ninein-interacting protein OS=Mus musculus OX=10090 GN=Aunip PE=2 SV=1//0	-2.8133	3.76E-08	1.20E-06
sp|Q8VCD3|LMA1L_MOUSE Protein ERGIC-53-like OS=Mus musculus OX=10090 GN=Lman1l PE=2 SV=2//0	-2.8107	0.0043541	0.03254
sp|Q8HY04|CD209_PAPHA CD209 antigen OS=Papio hamadryas OX=9557 GN=CD209 PE=3 SV=1//1.07071e-43	-2.7709	0.00011088	0.0015119
sp|O89029|MATN4_MOUSE Matrilin-4 OS=Mus musculus OX=10090 GN=Matn4 PE=1 SV=1//2.43813e-35	-2.7679	3.18E-19	6.84E-17
sp|Q925G2|CYBR1_MOUSE Cytochrome b reductase 1 OS=Mus musculus OX=10090 GN=Cybrd1 PE=1 SV=2//2.30676e-167	-2.7615	0.0063576	0.043859
sp|Q9CQX4|PAF15_MOUSE PCNA-associated factor OS=Mus musculus OX=10090 GN=Pclaf PE=1 SV=1//1.09644e-06	-2.7432	1.55E-38	3.44E-35
sp|P48967|MPIP3_MOUSE M-phase inducer phosphatase 3 OS=Mus musculus OX=10090 GN=Cdc25c PE=2 SV=2//0	-2.7333	6.20E-17	9.08E-15
sp|Q8C767|PPR3B_MOUSE Protein phosphatase 1 regulatory subunit 3B OS=Mus musculus OX=10090 GN=Ppp1r3b PE=1 SV=1//0	-2.6975	1.38E-08	4.87E-07
sp|O15072|ATS3_HUMAN A disintegrin and metalloproteinase with thrombospondin motifs 3 OS=Homo sapiens OX=9606 GN=ADAMTS3 PE=2 SV=4//0	-2.6675	8.42E-12	5.61E-10
sp|Q4VA61|DSCL1_MOUSE Down syndrome cell adhesion molecule-like protein 1 homolog OS=Mus musculus OX=10090 GN=Dscaml1 PE=1 SV=2//0	-2.6622	0.0011057	0.010569
sp|Q8CIG0|DEP1A_MOUSE DEP domain-containing protein 1A OS=Mus musculus OX=10090 GN=Depdc1a PE=1 SV=1//0	-2.6462	1.03E-18	2.08E-16
sp|Q9Z0S3|CLD14_MOUSE Claudin-14 OS=Mus musculus OX=10090 GN=Cldn14 PE=1 SV=2//3.49786e-116	-2.6365	0.0018844	0.016466
sp|Q7TSH9|ZN184_MOUSE Zinc finger protein 184 OS=Mus musculus OX=10090 GN=Zfp184 PE=2 SV=1//0	-2.6035	0.000095866	0.0013433
sp|Q07832|PLK1_MOUSE Serine/threonine-protein kinase PLK1 OS=Mus musculus OX=10090 GN=Plk1 PE=1 SV=2//0	-2.6018	3.80E-27	2.57E-24
sp|P28322|ETV4_MOUSE ETS translocation variant 4 OS=Mus musculus OX=10090 GN=Etv4 PE=2 SV=2//0	-2.5736	3.88E-28	3.04E-25
sp|Q3UR85|MYRF_MOUSE Myelin regulatory factor OS=Mus musculus OX=10090 GN=Myrf PE=1 SV=2//0	-2.5641	2.18E-10	1.09E-08
sp|Q91W10|S39A8_MOUSE Zinc transporter ZIP8 OS=Mus musculus OX=10090 GN=Slc39a8 PE=2 SV=1//2.19069e-21	-2.5627	2.19E-07	6.12E-06
sp|A8VU90|ANKL1_MOUSE Ankyrin repeat and LEM domain-containing protein 1 OS=Mus musculus OX=10090 GN=Ankle1 PE=2 SV=1//2.47519e-50	-2.5608	4.48E-10	2.09E-08
sp|Q9JJ78|TOPK_MOUSE Lymphokine-activated killer T-cell-originated protein kinase OS=Mus musculus OX=10090 GN=Pbk PE=1 SV=1//0	-2.5582	1.21E-26	7.96E-24
sp|Q7TN16|HHIP_MOUSE Hedgehog-interacting protein OS=Mus musculus OX=10090 GN=Hhip PE=1 SV=2//0	-2.5342	7.90E-15	8.28E-13

-//-	-2.5165	0.00018798	0.0023841
sp|P48299|EDN3_MOUSE Endothelin-3 OS=Mus musculus OX=10090 GN=Edn3 PE=2 SV=1//6.30577e-134	-2.5137	1.30E-16	1.85E-14
sp|Q9BPX3|CND3_HUMAN Condensin complex subunit 3 OS=Homo sapiens OX=9606 GN=NCAPG PE=1 SV=1//1.3769e-26	-2.495	2.77E-23	1.20E-20
sp|P31649|S6A13_MOUSE Sodium- and chloride-dependent GABA transporter 2 OS=Mus musculus OX=10090 GN=Slc6a13 PE=1 SV=1//0	-2.4887	0.0003247	0.0037792
sp|O08901|BUB1_MOUSE Mitotic checkpoint serine/threonine-protein kinase BUB1 OS=Mus musculus OX=10090 GN=Bub1 PE=1 SV=1//0	-2.487	7.91E-21	2.40E-18
sp|Q8MIS5|209L2_MACMU CD209 antigen-like protein 2 OS=Macaca mulatta OX=9544 GN=CD209L2 PE=1 SV=1//2.07193e-44	-2.4867	0.0065828	0.045057
sp|P48972|MYBB_MOUSE Myb-related protein B OS=Mus musculus OX=10090 GN=Mybl2 PE=1 SV=1//0	-2.4861	3.28E-22	1.23E-19
sp|O35137|ALX4_MOUSE Homeobox protein aristaless-like 4 OS=Mus musculus OX=10090 GN=Alx4 PE=1 SV=1//2.37786e-47	-2.4843	0.000012885	0.00023253
sp|P24860|CCNB1_MOUSE G2/mitotic-specific cyclin-B1 OS=Mus musculus OX=10090 GN=Ccnb1 PE=1 SV=3//0	-2.4759	3.61E-28	2.93E-25
sp|Q9WTK8|SPO11_MOUSE Meiotic recombination protein SPO11 OS=Mus musculus OX=10090 GN=Spo11 PE=1 SV=2//3.01657e-16	-2.4755	0.0017641	0.015611
sp|Q7TME2|SPAG5_MOUSE Sperm-associated antigen 5 OS=Mus musculus OX=10090 GN=Spag5 PE=1 SV=1//0	-2.4721	1.38E-22	5.34E-20
sp|Q6NZL8|SCUB1_MOUSE Signal peptide, CUB and EGF-like domain-containing protein 1 OS=Mus musculus OX=10090 GN=Scube1 PE=2 SV=2//0	-2.4715	7.93E-18	1.36E-15
sp|Q9QZ11|EXO1_MOUSE Exonuclease 1 OS=Mus musculus OX=10090 GN=Exo1 PE=2 SV=2//0	-2.4685	1.64E-14	1.64E-12
sp|Q99956|DUS9_HUMAN Dual specificity protein phosphatase 9 OS=Homo sapiens OX=9606 GN=DUSP9 PE=1 SV=1//1.09772e-15	-2.4624	0.0013826	0.012731
sp|Q9D1C1|UBE2C_MOUSE Ubiquitin-conjugating enzyme E2 C OS=Mus musculus OX=10090 GN=Ube2c PE=1 SV=1//6.46364e-104	-2.4562	3.22E-27	2.24E-24
sp|P48614|WN10B_MOUSE Protein Wnt-10b OS=Mus musculus OX=10090 GN=Wnt10b PE=2 SV=1//0	-2.4545	5.99E-21	1.92E-18
sp|Q922S8|KIF2C_MOUSE Kinesin-like protein KIF2C OS=Mus musculus OX=10090 GN=Kif2c PE=1 SV=1//0	-2.4472	6.76E-22	2.38E-19
sp|Q5BJU5|CNIH2_RAT Protein cornichon homolog 2 OS=Rattus norvegicus OX=10116 GN=Cnih2 PE=1 SV=1//1.81549e-95	-2.4362	0.00072411	0.0074355
sp|Q09143|CTR1_MOUSE High affinity cationic amino acid transporter 1 OS=Mus musculus OX=10090 GN=Slc7a1 PE=1 SV=1//0	-2.4321	9.26E-34	1.19E-30
sp|P70698|PYRG1_MOUSE CTP synthase 1 OS=Mus musculus OX=10090 GN=Ctps1 PE=1 SV=2//0	-2.4293	9.40E-06	0.00017571
sp|Q6P9P6|KIF11_MOUSE Kinesin-like protein KIF11 OS=Mus musculus OX=10090 GN=Kif11 PE=1 SV=1//0	-2.4224	3.39E-31	3.44E-28
sp|Q6GQX2|NCK5L_MOUSE Nck-associated protein 5-like OS=Mus musculus OX=10090 GN=Nckap5l PE=1 SV=1//0	-2.4211	9.97E-19	2.02E-16
sp|O08650|HYAS3_MOUSE Hyaluronan synthase 3 OS=Mus musculus OX=10090 GN=Has3 PE=1 SV=2//0	-2.4205	1.71E-09	7.23E-08
sp|Q9Z183|PADI4_MOUSE Protein-arginine deiminase type-4 OS=Mus musculus OX=10090 GN=Padi4 PE=2 SV=3//1.84386e-16	-2.4147	0.0028107	0.022929
sp|Q14B71|CDCA2_MOUSE Cell division cycle-associated protein 2 OS=Mus musculus OX=10090 GN=Cdca2 PE=1 SV=2//0	-2.414	4.81E-19	9.92E-17
sp|P35710|SOX5_MOUSE Transcription factor SOX-5 OS=Mus musculus OX=10090 GN=Sox5 PE=1 SV=2//0	-2.4075	2.70E-18	4.96E-16
sp|O89094|CASPE_MOUSE Caspase-14 OS=Mus musculus OX=10090 GN=Casp14 PE=1 SV=1//1.07646e-143	-2.4017	3.76E-35	6.10E-32
sp|Q7TSP5|VTCN1_MOUSE V-set domain containing T-cell activation inhibitor 1 OS=Mus musculus OX=10090 GN=Vtcn1 PE=1 SV=1//1.21896e-175	-2.4002	0.0072921	0.049016
sp|G5E8A8|TEKT5_MOUSE Tektin-5 OS=Mus musculus OX=10090 GN=Tekt5 PE=1 SV=1//0	-2.3913	0.00022278	0.0027496
sp|P60853|LZTS1_MOUSE Leucine zipper putative tumor suppressor 1 OS=Mus musculus OX=10090 GN=Lzts1 PE=2 SV=3//0	-2.3909	1.75E-07	5.00E-06
sp|Q3U827|RN180_MOUSE E3 ubiquitin-protein ligase RNF180 OS=Mus musculus OX=10090 GN=Rnf180 PE=1 SV=2//0	-2.3885	0.000068513	0.00099859
sp|Q66JQ7|KNL1_MOUSE Kinetochore scaffold 1 OS=Mus musculus OX=10090 GN=Knl1 PE=1 SV=3//0	-2.3876	3.56E-17	5.47E-15
sp|Q8C263|SKA3_MOUSE Spindle and kinetochore-associated protein 3 OS=Mus musculus OX=10090 GN=Ska3 PE=2 SV=1//0	-2.3844	2.62E-10	1.29E-08
sp|Q9QYU3|KCNG2_RAT Potassium voltage-gated channel subfamily G member 2 OS=Rattus norvegicus OX=10116 GN=Kcng2 PE=2 SV=1//0	-2.3796	1.05E-08	3.81E-07
sp|Q8BQ33|TICRR_MOUSE Treslin OS=Mus musculus OX=10090 GN=Ticrr PE=1 SV=2//0	-2.3791	1.57E-17	2.50E-15
sp|Q8BNJ3|NMNA2_MOUSE Nicotinamide/nicotinic acid mononucleotide adenylyltransferase 2 OS=Mus musculus OX=10090 GN=Nmnat2 PE=1 SV=1//5.61586e-12	-2.3706	5.04E-11	2.89E-09
sp|Q99NE5|RIMS1_MOUSE Regulating synaptic membrane exocytosis protein 1 OS=Mus musculus OX=10090 GN=Rims1 PE=1 SV=2//0	-2.3698	0.00095125	0.0093119
sp|Q01320|TOP2A_MOUSE DNA topoisomerase 2-alpha OS=Mus musculus OX=10090 GN=Top2a PE=1 SV=2//0	-2.3602	3.66E-35	6.10E-32
sp|O35942|NEK2_MOUSE Serine/threonine-protein kinase Nek2 OS=Mus musculus OX=10090 GN=Nek2 PE=1 SV=2//0	-2.3585	6.88E-23	2.89E-20
sp|Q6PFD6|KI18B_MOUSE Kinesin-like protein KIF18B OS=Mus musculus OX=10090 GN=Kif18b PE=2 SV=2//0	-2.3541	8.81E-18	1.48E-15
sp|Q8VDF2|UHRF1_MOUSE E3 ubiquitin-protein ligase UHRF1 OS=Mus musculus OX=10090 GN=Uhrf1 PE=1 SV=2//0	-2.3522	7.74E-31	7.53E-28

sp|Q6PFE3|RA54B_MOUSE DNA repair and recombination protein RAD54B OS=Mus musculus OX=10090 GN=Rad54b PE=2 SV=1//0	-2.3488	2.64E-10	1.31E-08
sp|Q8K596|NAC2_MOUSE Sodium/calcium exchanger 2 OS=Mus musculus OX=10090 GN=Slc8a2 PE=1 SV=1//0	-2.3471	0.000031979	0.00051757
sp|Q6RT24|CENPE_MOUSE Centromere-associated protein E OS=Mus musculus OX=10090 GN=Cenpe PE=1 SV=1//0	-2.3466	3.87E-26	2.35E-23
sp|Q8CFZ4|GPC3_MOUSE Glypican-3 OS=Mus musculus OX=10090 GN=Gpc3 PE=2 SV=1//0	-2.3416	0.00056203	0.0060122
sp|P31955|AREG_MOUSE Amphiregulin OS=Mus musculus OX=10090 GN=Areg PE=2 SV=1//3.47852e-111	-2.3402	1.59E-11	1.01E-09
sp|Q3UHU5|MTCL1_MOUSE Microtubule cross-linking factor 1 OS=Mus musculus OX=10090 GN=Mtcl1 PE=1 SV=1//6.75882e-09	-2.3398	0.00083536	0.0083419
sp|Q96L50|LLR1_HUMAN Leucine-rich repeat protein 1 OS=Homo sapiens OX=9606 GN=LRR1 PE=1 SV=2//0	-2.3291	2.03E-06	0.000044898
sp|Q9EQC9|CXXC4_RAT CXXC-type zinc finger protein 4 OS=Rattus norvegicus OX=10116 GN=Cxxc4 PE=1 SV=1//2.88336e-105	-2.324	4.86E-06	0.000098313
sp|P22725|WNT5A_MOUSE Protein Wnt-5a OS=Mus musculus OX=10090 GN=Wnt5a PE=1 SV=2//0	-2.3212	1.52E-30	1.37E-27
sp|E9PVX6|KI67_MOUSE Proliferation marker protein Ki-67 OS=Mus musculus OX=10090 GN=Mki67 PE=1 SV=1//0	-2.3158	2.55E-34	3.64E-31
sp|O70451|MOT2_MOUSE Monocarboxylate transporter 2 OS=Mus musculus OX=10090 GN=Slc16a7 PE=1 SV=1//0	-2.3051	6.19E-06	0.00012178
sp|Q8BJW7|EME1_MOUSE Crossover junction endonuclease EME1 OS=Mus musculus OX=10090 GN=Eme1 PE=1 SV=1//0	-2.3003	7.76E-08	0.00000236
sp|Q80VW5|WHRN_MOUSE Whirlin OS=Mus musculus OX=10090 GN=Whrn PE=1 SV=3//0	-2.3001	8.35E-14	7.49E-12
sp|Q6P9L6|KIF15_MOUSE Kinesin-like protein KIF15 OS=Mus musculus OX=10090 GN=Kif15 PE=1 SV=1//0	-2.2971	1.38E-17	2.21E-15
sp|Q9JJ66|CDC20_MOUSE Cell division cycle protein 20 homolog OS=Mus musculus OX=10090 GN=Cdc20 PE=1 SV=2//0	-2.2966	3.70E-25	1.92E-22
sp|Q8CIB9|ESCO2_MOUSE N-acetyltransferase ESCO2 OS=Mus musculus OX=10090 GN=Esco2 PE=2 SV=3//0	-2.2962	4.32E-18	7.73E-16
sp|Q9CXH7|SGO1_MOUSE Shugoshin 1 OS=Mus musculus OX=10090 GN=Sgo1 PE=2 SV=1//0	-2.2875	2.33E-15	2.71E-13
sp|P49454|CENPF_HUMAN Centromere protein F OS=Homo sapiens OX=9606 GN=CENPF PE=1 SV=2//3.87822e-120	-2.2778	5.17E-28	3.81E-25
sp|P47806|GLI1_MOUSE Zinc finger protein GLI1 OS=Mus musculus OX=10090 GN=Gli1 PE=1 SV=4//0	-2.2761	0.00036579	0.0041659
sp|P59511|ATS20_MOUSE A disintegrin and metalloproteinase with thrombospondin motifs 20 OS=Mus musculus OX=10090 GN=Adamts20 PE=2 SV=2//5.97891e-29	-2.265	4.27E-18	7.69E-16
sp|Q8BZB3|TM266_MOUSE Transmembrane protein 266 OS=Mus musculus OX=10090 GN=Tmem266 PE=1 SV=3//7.67666e-93	-2.2589	0.0022317	0.018912
sp|P14733|LMNB1_MOUSE Lamin-B1 OS=Mus musculus OX=10090 GN=Lmnb1 PE=1 SV=3//0	-2.2439	2.00E-30	1.73E-27
sp|Q0VBD2|MCM10_MOUSE Protein MCM10 homolog OS=Mus musculus OX=10090 GN=Mcm10 PE=1 SV=1//0	-2.241	1.72E-14	1.71E-12
sp|P51943|CCNA2_MOUSE Cyclin-A2 OS=Mus musculus OX=10090 GN=Ccna2 PE=1 SV=2//0	-2.238	1.25E-26	7.98E-24
sp|P48754|BRCA1_MOUSE Breast cancer type 1 susceptibility protein homolog OS=Mus musculus OX=10090 GN=Brca1 PE=1 SV=3//0	-2.2346	3.04E-19	6.60E-17
sp|Q9CPV1|SKA1_MOUSE Spindle and kinetochore-associated protein 1 OS=Mus musculus OX=10090 GN=Ska1 PE=2 SV=2//2.69483e-178	-2.2338	1.23E-08	4.39E-07
sp|Q9DBX7|HEYL_MOUSE Hairy/enhancer-of-split related with YRPW motif-like protein OS=Mus musculus OX=10090 GN=Heyl PE=1 SV=2//0	-2.2224	2.60E-14	2.53E-12
-//-	-2.2203	0.00097448	0.0094897
sp|Q9CPY7|AMPL_MOUSE Cytosol aminopeptidase OS=Mus musculus OX=10090 GN=Lap3 PE=1 SV=3//0	-2.198	0.0013215	0.012251
sp|B7ZNG4|TROAP_MOUSE Tastin OS=Mus musculus OX=10090 GN=Troap PE=2 SV=1//0	-2.1918	2.44E-10	1.22E-08
sp|Q9H611|PIF1_HUMAN ATP-dependent DNA helicase PIF1 OS=Homo sapiens OX=9606 GN=PIF1 PE=1 SV=2//0	-2.1909	6.01E-13	4.79E-11
sp|Q52KG5|KI26A_MOUSE Kinesin-like protein KIF26A OS=Mus musculus OX=10090 GN=Kif26a PE=1 SV=2//0	-2.1888	1.31E-06	0.000030462
sp|O08696|FOXM1_MOUSE Forkhead box protein M1 OS=Mus musculus OX=10090 GN=Foxm1 PE=1 SV=2//0	-2.1882	1.66E-22	6.31E-20
sp|P30276|CCNB2_MOUSE G2/mitotic-specific cyclin-B2 OS=Mus musculus OX=10090 GN=Ccnb2 PE=1 SV=2//0	-2.186	8.83E-15	9.18E-13
sp|Q922Y2|TRI59_MOUSE Tripartite motif-containing protein 59 OS=Mus musculus OX=10090 GN=Trim59 PE=1 SV=2//0	-2.1858	1.56E-26	9.71E-24
sp|Q61884|MNS1_MOUSE Meiosis-specific nuclear structural protein 1 OS=Mus musculus OX=10090 GN=Mns1 PE=1 SV=1//0	-2.1842	0.0012732	0.011899
sp|P11440|CDK1_MOUSE Cyclin-dependent kinase 1 OS=Mus musculus OX=10090 GN=Cdk1 PE=1 SV=3//0	-2.1835	4.01E-24	1.84E-21
sp|A2APB8|TPX2_MOUSE Targeting protein for Xklp2 OS=Mus musculus OX=10090 GN=Tpx2 PE=1 SV=1//0	-2.18	8.51E-25	4.23E-22
sp|Q5XIP6|FEN1_RAT Flap endonuclease 1 OS=Rattus norvegicus OX=10116 GN=Fen1 PE=2 SV=1//0	-2.1789	0.00010613	0.0014578
sp|Q99M54|CDCA3_MOUSE Cell division cycle-associated protein 3 OS=Mus musculus OX=10090 GN=Cdca3 PE=1 SV=2//0	-2.1787	1.27E-21	4.28E-19
sp|O70126|AURKB_MOUSE Aurora kinase B OS=Mus musculus OX=10090 GN=Aurkb PE=1 SV=2//0	-2.1786	8.37E-21	2.51E-18

sp|Q6S7F2|E2F7_MOUSE Transcription factor E2F7 OS=Mus musculus OX=10090 GN=E2f7 PE=1 SV=1//0	-2.1719	5.48E-13	4.38E-11
sp|Q9Z0R9|FADS2_MOUSE Fatty acid desaturase 2 OS=Mus musculus OX=10090 GN=Fads2 PE=1 SV=1//0	-2.165	9.37E-20	2.24E-17
sp|O35914|BNC1_MOUSE Zinc finger protein basonuclin-1 OS=Mus musculus OX=10090 GN=Bnc1 PE=1 SV=1//0	-2.1602	1.22E-15	1.51E-13
sp|O70201|BIRC5_MOUSE Baculoviral IAP repeat-containing protein 5 OS=Mus musculus OX=10090 GN=Birc5 PE=1 SV=1//1.23549e-06	-2.1554	5.03E-11	2.89E-09
sp|Q99P69|NUF2_MOUSE Kinetochore protein Nuf2 OS=Mus musculus OX=10090 GN=Nuf2 PE=1 SV=2//0	-2.153	1.19E-17	1.92E-15
sp|Q9Z1N2|ORC1_MOUSE Origin recognition complex subunit 1 OS=Mus musculus OX=10090 GN=Orc1 PE=1 SV=2//0	-2.1486	2.53E-07	6.98E-06
sp|Q8BT07|CEP55_MOUSE Centrosomal protein of 55 kDa OS=Mus musculus OX=10090 GN=Cep55 PE=1 SV=2//0	-2.1415	6.50E-16	8.33E-14
sp|Q80WQ8|M18BP_MOUSE Mis18-binding protein 1 OS=Mus musculus OX=10090 GN=Mis18bp1 PE=1 SV=1//0	-2.1397	1.70E-15	2.05E-13
sp|Q9EQD0|FZD5_MOUSE Frizzled-5 OS=Mus musculus OX=10090 GN=Fzd5 PE=1 SV=3//0	-2.1369	2.82E-08	9.25E-07
sp|Q61846|MELK_MOUSE Maternal embryonic leucine zipper kinase OS=Mus musculus OX=10090 GN=Melk PE=1 SV=2//0	-2.1363	8.40E-18	1.42E-15
sp|Q3TLR7|DTL_MOUSE Denticleless protein homolog OS=Mus musculus OX=10090 GN=Dtl PE=1 SV=2//0	-2.13	6.10E-17	8.99E-15
sp|Q9JIN6|KCMB4_MOUSE Calcium-activated potassium channel subunit beta-4 OS=Mus musculus OX=10090 GN=Kcnmb4 PE=1 SV=1//9.91174e-47	-2.13	0.00331	0.026067
sp|Q9Z261|CLD7_MOUSE Claudin-7 OS=Mus musculus OX=10090 GN=Cldn7 PE=1 SV=1//3.70848e-134	-2.1277	0.0026888	0.022142
sp|P54227|STMN1_MOUSE Stathmin OS=Mus musculus OX=10090 GN=Stmn1 PE=1 SV=2//6.73143e-95	-2.1232	9.75E-30	8.18E-27
sp|Q8BHX3|BOREA_MOUSE Borealin OS=Mus musculus OX=10090 GN=Cdca8 PE=1 SV=2//1.43837e-65	-2.1229	2.05E-20	5.43E-18
sp|Q8TE56|ATS17_HUMAN A disintegrin and metalloproteinase with thrombospondin motifs 17 OS=Homo sapiens OX=9606 GN=ADAMTS17 PE=2 SV=2//0	-2.1139	8.00E-09	2.97E-07
sp|Q32P12|CQ053_MOUSE Uncharacterized protein C17orf53 homolog OS=Mus musculus OX=10090 PE=2 SV=2//0	-2.111	0.000023172	0.00038821
sp|P48615|WNT11_MOUSE Protein Wnt-11 OS=Mus musculus OX=10090 GN=Wnt11 PE=2 SV=1//0	-2.1109	1.32E-09	5.72E-08
sp|P70181|PI51B_MOUSE Phosphatidylinositol 4-phosphate 5-kinase type-1 beta OS=Mus musculus OX=10090 GN=Pip5k1b PE=1 SV=1//0	-2.1098	0.0029186	0.023541
sp|P97329|KI20A_MOUSE Kinesin-like protein KIF20A OS=Mus musculus OX=10090 GN=Kif20a PE=1 SV=1//0	-2.0996	2.28E-11	1.40E-09
sp|Q80YR7|CLSPN_MOUSE Claspin OS=Mus musculus OX=10090 GN=Clspn PE=1 SV=2//0	-2.0976	1.37E-13	1.21E-11
sp|Q8BVC4|CCD68_MOUSE Coiled-coil domain-containing protein 68 OS=Mus musculus OX=10090 GN=Ccdc68 PE=2 SV=1//0	-2.0963	0.000071025	0.0010284
sp|Q3V300|KIF22_MOUSE Kinesin-like protein KIF22 OS=Mus musculus OX=10090 GN=Kif22 PE=2 SV=2//0	-2.0892	5.12E-12	3.50E-10
sp|Q8BH53|CFA69_MOUSE Cilia- and flagella-associated protein 69 OS=Mus musculus OX=10090 GN=Cfap69 PE=2 SV=1//0	-2.0846	0.0074522	0.049931
sp|Q9Z179|SHCBP_MOUSE SHC SH2 domain-binding protein 1 OS=Mus musculus OX=10090 GN=Shcbp1 PE=1 SV=1//0	-2.084	9.87E-15	1.01E-12
sp|P28667|MRP_MOUSE MARCKS-related protein OS=Mus musculus OX=10090 GN=Marcksl1 PE=1 SV=2//2.34804e-62	-2.0678	3.03E-17	4.69E-15
sp|O70445|BARD1_MOUSE BRCA1-associated RING domain protein 1 OS=Mus musculus OX=10090 GN=Bard1 PE=2 SV=1//0	-2.0676	1.40E-12	1.03E-10
sp|Q8K368|FANCI_MOUSE Fanconi anemia group I protein homolog OS=Mus musculus OX=10090 GN=Fanci PE=1 SV=2//0	-2.064	1.21E-08	4.31E-07
sp|Q07139|ECT2_MOUSE Protein ECT2 OS=Mus musculus OX=10090 GN=Ect2 PE=1 SV=2//0	-2.0594	4.81E-19	9.92E-17
sp|Q8BHE0|PRR11_MOUSE Proline-rich protein 11 OS=Mus musculus OX=10090 GN=Prr11 PE=2 SV=1//4.25847e-172	-2.0531	4.95E-16	6.47E-14
sp|Q99N50|SYTL2_MOUSE Synaptotagmin-like protein 2 OS=Mus musculus OX=10090 GN=Sytl2 PE=1 SV=2//0	-2.0528	0.00047897	0.0052436
sp|O75355|ENTP3_HUMAN Ectonucleoside triphosphate diphosphohydrolase 3 OS=Homo sapiens OX=9606 GN=ENTPD3 PE=1 SV=2//0	-2.0378	1.04E-08	3.77E-07
sp|P30935|SSR3_MOUSE Somatostatin receptor type 3 OS=Mus musculus OX=10090 GN=Sstr3 PE=2 SV=2//0	-2.0377	0.00533	0.038337
sp|Q8R5H6|WASF1_MOUSE Wiskott-Aldrich syndrome protein family member 1 OS=Mus musculus OX=10090 GN=Wasf1 PE=1 SV=2//2.15781e-54	-2.0357	2.79E-10	1.36E-08
sp|Q9CPY3|CDCA5_MOUSE Sororin OS=Mus musculus OX=10090 GN=Cdca5 PE=1 SV=1//4.39688e-144	-2.0342	2.71E-12	1.93E-10
sp|Q60988|STIL_MOUSE SCL-interrupting locus protein homolog OS=Mus musculus OX=10090 GN=Stil PE=1 SV=1//0	-2.031	1.39E-10	7.24E-09
sp|Q5XJV6|LMTK3_MOUSE Serine/threonine-protein kinase LMTK3 OS=Mus musculus OX=10090 GN=Lmtk3 PE=1 SV=1//0	-2.0306	0.00015969	0.0020663
sp|Q06831|SOX4_MOUSE Transcription factor SOX-4 OS=Mus musculus OX=10090 GN=Sox4 PE=1 SV=2//1.76168e-155	-2.0164	1.35E-22	5.30E-20
sp|Q8C4M7|CENPU_MOUSE Centromere protein U OS=Mus musculus OX=10090 GN=Cenpu PE=1 SV=2//3.61585e-09	-2.0126	0.000051139	0.00077508
sp|Q9D083|SPC24_MOUSE Kinetochore protein Spc24 OS=Mus musculus OX=10090 GN=Spc24 PE=1 SV=1//2.52085e-127	-2.0112	7.25E-12	4.87E-10
sp|O35216|CENPA_MOUSE Histone H3-like centromeric protein A OS=Mus musculus OX=10090 GN=Cenpa PE=2 SV=1//4.79131e-56	-2.0071	1.86E-10	9.43E-09

sp|Q92522|H1X_HUMAN Histone H1x OS=Homo sapiens OX=9606 GN=H1FX PE=1 SV=1//1.7481e-13	-2.006	0.0067821	0.046136
sp|Q96FE5|LIGO1_HUMAN Leucine-rich repeat and immunoglobulin-like domain-containing nogo receptor-interacting protein 1 OS=Homo sapiens OX=9606 GN=LINGO1 PE=1 SV=2//0	-2.0049	3.90E-11	2.31E-09
sp|P35546|RET_MOUSE Proto-oncogene tyrosine-protein kinase receptor Ret OS=Mus musculus OX=10090 GN=Ret PE=1 SV=2//4.01034e-23	-2.0034	1.86E-18	3.62E-16

Supplementary Data 2B

Blast swiss prot (UPREGULATED, >2FC)	log2FoldChange	pval	padj
sp|P50228|CXCL5_MOUSE C-X-C motif chemokine 5 OS=Mus musculus OX=10090 GN=Cxcl5 PE=1 SV=2//3.25721e-72	9.8187	7.57E-09	5.27E-07
sp|P54987|IRG1_MOUSE Cis-aconitate decarboxylase OS=Mus musculus OX=10090 GN=Acod1 PE=1 SV=2//0	8.4023	1.75E-38	1.50E-35
sp|P04918|SAA3_MOUSE Serum amyloid A-3 protein OS=Mus musculus OX=10090 GN=Saa3 PE=1 SV=1//4.23577e-65	7.7023	2.76E-97	1.32E-93
sp|Q9JKE2|TREM1_MOUSE Triggering receptor expressed on myeloid cells 1 OS=Mus musculus OX=10090 GN=Trem1 PE=1 SV=1//1.11941e-135	7.4878	5.88E-20	1.72E-17
sp|Q6W5C0|CXCL3_MOUSE C-X-C motif chemokine 3 OS=Mus musculus OX=10090 GN=Cxcl3 PE=2 SV=1//1.74753e-42	7.3754	3.98E-16	8.23E-14
sp|P13541|MYH3_MOUSE Myosin-3 OS=Mus musculus OX=10090 GN=Myh3 PE=2 SV=2//0	6.9183	3.71E-111	2.22E-107
sp|P33435|MMP13_MOUSE Collagenase 3 OS=Mus musculus OX=10090 GN=Mmp13 PE=1 SV=1//0	6.7994	6.56E-08	3.77E-06
sp|P79457|UTY_MOUSE Histone demethylase UTY OS=Mus musculus OX=10090 GN=Uty PE=1 SV=2//0	6.7437	3.27E-62	7.12E-59
sp|P10889|CXCL2_MOUSE C-X-C motif chemokine 2 OS=Mus musculus OX=10090 GN=Cxcl2 PE=1 SV=2//1.62011e-51	6.4361	4.44E-22	1.52E-19
sp|Q9Z2H6|CLC4D_MOUSE C-type lectin domain family 4 member D OS=Mus musculus OX=10090 GN=Clec4d PE=1 SV=1//3.94725e-160	6.1534	2.26E-20	6.94E-18
sp|Q3TTY5|K22E_MOUSE Keratin, type II cytoskeletal 2 epidermal OS=Mus musculus OX=10090 GN=Krt2 PE=1 SV=1//0	6.0341	3.98E-60	7.94E-57
sp|Q2Q5T5|MYMX_MOUSE Protein myomixer OS=Mus musculus OX=10090 GN=Mymx PE=1 SV=1//5.46971e-21	5.8656	1.66E-07	8.67E-06
sp|P19123|TNNC1_MOUSE Troponin C, slow skeletal and cardiac muscles OS=Mus musculus OX=10090 GN=Tnnc1 PE=1 SV=1//2.84479e-100	5.6624	5.63E-59	1.04E-55
sp|Q6P640|ACTC_XENTR Actin, alpha cardiac muscle 1 OS=Xenopus tropicalis OX=8364 GN=actc1 PE=2 SV=1//0	5.4484	1.46E-82	5.00E-79
sp|Q3LRV9|TRML4_MOUSE Trem-like transcript 4 protein OS=Mus musculus OX=10090 GN=Treml4 PE=1 SV=1//9.67788e-163	5.3375	6.02E-05	0.0016164
sp|Q9R0Q8|CLC4E_MOUSE C-type lectin domain family 4 member E OS=Mus musculus OX=10090 GN=Clec4e PE=1 SV=1//1.58514e-19	5.3051	3.16E-43	3.29E-40
sp|Q9D1N4|MYMK_MOUSE Protein myomaker OS=Mus musculus OX=10090 GN=Mymk PE=1 SV=1//2.39868e-121	5.2989	6.47E-36	4.43E-33
sp|P31725|S10A9_MOUSE Protein S100-A9 OS=Mus musculus OX=10090 GN=S100a9 PE=1 SV=3//6.17774e-69	5.2604	0.00017031	0.0039842
-//-	5.1732	1.03E-79	3.08E-76
sp|O88809|DCX_MOUSE Neuronal migration protein doublecortin OS=Mus musculus OX=10090 GN=Dcx PE=1 SV=1//0	5.1125	4.20E-08	2.47E-06
sp|P01865|GCAM_MOUSE Ig gamma-2A chain C region, membrane-bound form OS=Mus musculus OX=10090 GN=Igh-1a PE=1 SV=3//0	5.1079	7.58E-05	0.0019866
sp|Q2WG77|RIPP1_MOUSE Protein ripply1 OS=Mus musculus OX=10090 GN=Ripply1 PE=2 SV=1//7.3048e-74	5.0211	0.00057014	0.011249
-//-	4.979	1.13E-05	0.00036663
sp|P01655|KV3A2_MOUSE Ig kappa chain V-III region PC 7132 OS=Mus musculus OX=10090 PE=1 SV=1//4.02935e-68	4.907	0.00035892	0.0076488
sp|O70561|SPR2J_MOUSE Putative small proline-rich protein 2J OS=Mus musculus OX=10090 GN=Sprr2j PE=2 SV=2//3.10502e-14	4.9018	0.0023542	0.036428
sp|P13542|MYH8_MOUSE Myosin-8 OS=Mus musculus OX=10090 GN=Myh8 PE=2 SV=2//0	4.8781	2.46E-20	7.45E-18
sp|Q9CR42|ANKR1_MOUSE Ankyrin repeat domain-containing protein 1 OS=Mus musculus OX=10090 GN=Ankrd1 PE=1 SV=1//0	4.8634	2.46E-54	3.92E-51
sp|P01635|KV5A3_MOUSE Ig kappa chain V-V region K2 (Fragment) OS=Mus musculus OX=10090 PE=1 SV=1//9.3122e-62	4.7353	0.0025233	0.038158
-//-	4.6878	5.17E-09	3.77E-07
sp|P35343|CXCR2_MOUSE C-X-C chemokine receptor type 2 OS=Mus musculus OX=10090 GN=Cxcr2 PE=2 SV=1//0	4.6788	9.35E-08	5.19E-06
sp|Q7TNB2|TNNT1_RAT Troponin T, slow skeletal muscle OS=Rattus norvegicus OX=10116 GN=Tnnt1 PE=1 SV=3//1.4801e-88	4.6548	5.41E-73	1.44E-69
sp|Q9JKE1|TREM3_MOUSE Triggering receptor expressed on myeloid cells 3 OS=Mus musculus OX=10090 GN=Trem3 PE=1 SV=1//8.48669e-117	4.5229	0.0025162	0.038158
sp|P10855|CCL3_MOUSE C-C motif chemokine 3 OS=Mus musculus OX=10090 GN=Ccl3 PE=1 SV=2//9.24745e-61	4.4672	4.83E-11	4.95E-09
sp|Q9JI58|RAE1D_MOUSE Retinoic acid early-inducible protein 1-delta OS=Mus musculus OX=10090 GN=Raet1d PE=1 SV=1//1.12769e-154	4.4579	0.0033832	0.048613
sp|Q60673|PTPRN_MOUSE Receptor-type tyrosine-protein phosphatase-like N OS=Mus musculus OX=10090 GN=Ptprn PE=1 SV=2//0	4.4081	2.10E-07	1.07E-05
sp|P12979|MYOG_MOUSE Myogenin OS=Mus musculus OX=10090 GN=Myog PE=1 SV=2//3.2593e-148	4.4066	4.84E-53	6.82E-50
sp|P10923|OSTP_MOUSE Osteopontin OS=Mus musculus OX=10090 GN=Spp1 PE=1 SV=1//4.69326e-165	4.386	3.13E-25	1.27E-22
sp|Q00780|CO8A1_MOUSE Collagen alpha-1(VIII) chain OS=Mus musculus OX=10090 GN=Col8a1 PE=1 SV=3//2.98412e-35	4.3757	2.27E-10	2.13E-08

sp|P06327|HVM52_MOUSE Ig heavy chain V region VH558 A1/A4 OS=Mus musculus OX=10090 GN=Gm5629 PE=2 SV=1//2.16527e-53	4.3752	8.46E-05	0.0021703
sp|P01633|KV5A1_MOUSE Ig kappa chain V19-17 OS=Mus musculus OX=10090 GN=Igk-V19-17 PE=1 SV=1//8.40005e-63	4.362	0.00040802	0.0085656
sp|Q8HYP9|CCL17_MACMU C-C motif chemokine 17 OS=Macaca mulatta OX=9544 GN=CCL17 PE=3 SV=1//1.81071e-33	4.3579	5.89E-18	1.41E-15
sp|O70561|SPR2J_MOUSE Putative small proline-rich protein 2J OS=Mus musculus OX=10090 GN=Sprr2j PE=2 SV=2//2.20503e-13	4.2819	8.58E-06	0.00028731
sp|P70310|NTR2_MOUSE Neurotensin receptor type 2 OS=Mus musculus OX=10090 GN=Ntsr2 PE=2 SV=2//0	4.2214	0.00047453	0.0096653
sp|P09541|MYL4_MOUSE Myosin light chain 4 OS=Mus musculus OX=10090 GN=Myl4 PE=1 SV=3//1.53837e-24	4.1377	1.36E-63	3.25E-60
sp|P51667|MLRV_MOUSE Myosin regulatory light chain 2, ventricular/cardiac muscle isoform OS=Mus musculus OX=10090 GN=Myl2 PE=1 SV=3//2.39465e-116	4.1292	0.00057426	0.011312
sp|P35174|CYT2_MOUSE Stefin-2 OS=Mus musculus OX=10090 GN=Stfa2 PE=3 SV=2//2.84393e-67	4.0641	0.00062468	0.012077
sp|O70561|SPR2J_MOUSE Putative small proline-rich protein 2J OS=Mus musculus OX=10090 GN=Sprr2j PE=2 SV=2//1.02871e-10	4.0143	0.0026976	0.040309
sp|P04760|ACHG_MOUSE Acetylcholine receptor subunit gamma OS=Mus musculus OX=10090 GN=Chrng PE=2 SV=1//0	3.9995	9.73E-12	1.19E-09
sp|Q9D1D6|CTHR1_MOUSE Collagen triple helix repeat-containing protein 1 OS=Mus musculus OX=10090 GN=Cthrc1 PE=2 SV=2//3.39283e-164	3.9662	1.09E-07	5.96E-06
sp|P18529|HVM58_MOUSE Ig heavy chain V region 5-76 OS=Mus musculus OX=10090 PE=1 SV=1//1.77482e-72	3.9532	0.00075179	0.014188
sp|P43030|CXCL7_PIG Platelet basic protein OS=Sus scrofa OX=9823 GN=PPBP PE=1 SV=1//5.86702e-21	3.9512	0.0018396	0.029732
-//-	3.9308	1.46E-53	2.19E-50
sp|P06315|KV502_HUMAN Immunoglobulin kappa variable 5-2 OS=Homo sapiens OX=9606 GN=IGKV5-2 PE=1 SV=1//1.36525e-38	3.9135	0.0029895	0.043877
sp|P51480|CDN2A_MOUSE Cyclin-dependent kinase inhibitor 2A OS=Mus musculus OX=10090 GN=Cdkn2a PE=1 SV=2//1.57272e-86	3.9047	1.41E-07	7.49E-06
sp|P97797|SHPS1_MOUSE Tyrosine-protein phosphatase non-receptor type substrate 1 OS=Mus musculus OX=10090 GN=Sirpa PE=1 SV=1//3.17488e-149	3.8636	1.48E-12	1.99E-10
sp|O55188|DMP1_MOUSE Dentin matrix acidic phosphoprotein 1 OS=Mus musculus OX=10090 GN=Dmp1 PE=2 SV=2//1.68168e-166	3.8559	1.48E-06	6.16E-05
sp|O70138|MMP8_MOUSE Neutrophil collagenase OS=Mus musculus OX=10090 GN=Mmp8 PE=2 SV=2//0	3.8135	5.21E-08	3.02E-06
sp|Q07440|B2LA1_MOUSE Bcl-2-related protein A1 OS=Mus musculus OX=10090 GN=Bcl2a1 PE=1 SV=1//9.23322e-117	3.788	2.28E-08	1.42E-06
sp|Q80YX1|TENA_MOUSE Tenascin OS=Mus musculus OX=10090 GN=Tnc PE=1 SV=1//0	3.7813	2.07E-07	1.06E-05
sp|P12032|TIMP1_MOUSE Metalloproteinase inhibitor 1 OS=Mus musculus OX=10090 GN=Timp1 PE=1 SV=2//1.38285e-130	3.7334	4.20E-33	2.58E-30
sp|P01878|IGHA_MOUSE Ig alpha chain C region OS=Mus musculus OX=10090 PE=1 SV=1//0	3.7158	3.20E-13	4.68E-11
sp|Q63111|CEAM3_RAT Carcinoembryonic antigen-related cell adhesion molecule 3 OS=Rattus norvegicus OX=10116 GN=Ceacam3 PE=2 SV=1//0	3.6958	0.0029752	0.04372
sp|P12850|GROA_MOUSE Growth-regulated alpha protein OS=Mus musculus OX=10090 GN=Cxcl1 PE=1 SV=1//3.07076e-43	3.6876	1.36E-06	5.73E-05
sp|P27005|S10A8_MOUSE Protein S100-A8 OS=Mus musculus OX=10090 GN=S100a8 PE=1 SV=3//1.23147e-59	3.5839	0.0014516	0.024521
sp|P24699|MYF5_MOUSE Myogenic factor 5 OS=Mus musculus OX=10090 GN=Myf5 PE=1 SV=1//6.31678e-161	3.5833	2.43E-07	1.22E-05
sp|Q6SJQ7|CLM1_MOUSE CMRF35-like molecule 1 OS=Mus musculus OX=10090 GN=Cd300lf PE=1 SV=1//7.41183e-82	3.5599	5.81E-22	1.93E-19
sp|Q9ES30|C1QT3_MOUSE Complement C1q tumor necrosis factor-related protein 3 OS=Mus musculus OX=10090 GN=C1qtnf3 PE=2 SV=1//3.79584e-130	3.5552	5.58E-09	4.03E-07
sp|P18088|DCE1_RAT Glutamate decarboxylase 1 OS=Rattus norvegicus OX=10116 GN=Gad1 PE=2 SV=1//0	3.5212	5.71E-06	0.00020006
sp|Q80WT4|GP176_MOUSE G-protein coupled receptor 176 OS=Mus musculus OX=10090 GN=Gpr176 PE=1 SV=1//0	3.4811	2.69E-07	1.33E-05
sp|Q8CIP5|DISP2_MOUSE Protein dispatched homolog 2 OS=Mus musculus OX=10090 GN=Disp2 PE=1 SV=1//0	3.4807	4.32E-06	0.00015833
sp|Q61468|MSLN_MOUSE Mesothelin OS=Mus musculus OX=10090 GN=Msln PE=1 SV=1//0	3.4332	1.37E-09	1.10E-07
sp|Q9Z0D9|CX3C1_MOUSE CX3C chemokine receptor 1 OS=Mus musculus OX=10090 GN=Cx3cr1 PE=1 SV=1//0	3.4266	3.79E-05	0.0010766
sp|P53347|ONCM_MOUSE Oncostatin-M OS=Mus musculus OX=10090 GN=Osm PE=1 SV=1//2.48409e-119	3.4079	4.20E-07	2.00E-05
sp|O88552|CLD2_MOUSE Claudin-2 OS=Mus musculus OX=10090 GN=Cldn2 PE=1 SV=1//1.10601e-128	3.3996	0.00027924	0.0061645
sp|P09535|IGF2_MOUSE Insulin-like growth factor II OS=Mus musculus OX=10090 GN=Igf2 PE=1 SV=1//1.30256e-90	3.388	1.22E-49	1.54E-46
sp|P47239|PAX7_MOUSE Paired box protein Pax-7 OS=Mus musculus OX=10090 GN=Pax7 PE=1 SV=2//0	3.3719	1.62E-11	1.85E-09
sp|O70561|SPR2J_MOUSE Putative small proline-rich protein 2J OS=Mus musculus OX=10090 GN=Sprr2j PE=2 SV=2//1.77605e-12	3.3517	0.0030037	0.044032
sp|P01592|IGJ_MOUSE Immunoglobulin J chain OS=Mus musculus OX=10090 GN=Jchain PE=1 SV=4//4.60844e-110	3.3457	1.40E-11	1.64E-09
sp|P04756|ACHA_MOUSE Acetylcholine receptor subunit alpha OS=Mus musculus OX=10090 GN=Chrna1 PE=1 SV=1//0	3.3331	1.55E-38	1.38E-35

sp|Q5TFQ8|SIRBL_HUMAN Signal-regulatory protein beta-1 isoform 3 OS=Homo sapiens OX=9606 GN=SIRPB1 PE=1 SV=1//2.04372e-144	3.2971	3.18E-07	1.56E-05
sp|O35744|CHIL3_MOUSE Chitinase-like protein 3 OS=Mus musculus OX=10090 GN=Chil3 PE=1 SV=2//0	3.2913	0.0024273	0.037238
sp|P70375|FA7_MOUSE Coagulation factor VII OS=Mus musculus OX=10090 GN=F7 PE=1 SV=1//0	3.2864	5.74E-22	1.93E-19
sp|P97430|SLPI_MOUSE Antileukoproteinase OS=Mus musculus OX=10090 GN=Slpi PE=1 SV=1//1.51715e-85	3.2767	4.47E-05	0.0012365
sp|P14097|CCL4_MOUSE C-C motif chemokine 4 OS=Mus musculus OX=10090 GN=Ccl4 PE=3 SV=3//7.79839e-47	3.2766	4.49E-06	0.00016285
sp|A6NI73|LIRA5_HUMAN Leukocyte immunoglobulin-like receptor subfamily A member 5 OS=Homo sapiens OX=9606 GN=LILRA5 PE=1 SV=1//3.6968e-91	3.2728	0.0014843	0.025003
sp|Q5SS00|ZDBF2_MOUSE DBF4-type zinc finger-containing protein 2 homolog OS=Mus musculus OX=10090 GN=Zdbf2 PE=2 SV=1//3.7793e-19	3.2513	1.67E-15	3.21E-13
sp|P97797|SHPS1_MOUSE Tyrosine-protein phosphatase non-receptor type substrate 1 OS=Mus musculus OX=10090 GN=Sirpa PE=1 SV=1//8.11212e-51	3.2503	1.37E-06	5.78E-05
sp|Q00981|UCHL1_RAT Ubiquitin carboxyl-terminal hydrolase isozyme L1 OS=Rattus norvegicus OX=10116 GN=Uchl1 PE=1 SV=2//1.24472e-161	3.2239	1.20E-17	2.86E-15
sp|O55071|CP2BJ_MOUSE Cytochrome P450 2B19 OS=Mus musculus OX=10090 GN=Cyp2b19 PE=2 SV=1//0	3.1887	6.52E-14	1.04E-11
sp|Q6P3Y9|PONL1_MOUSE Podocan-like protein 1 OS=Mus musculus OX=10090 GN=Podnl1 PE=1 SV=3//3.09845e-06	3.155	1.34E-06	5.67E-05
sp|Q9CWB5|PGPIL_MOUSE Pyroglutamyl-peptidase 1-like protein OS=Mus musculus OX=10090 GN=Pgpep1l PE=2 SV=1//5.17089e-88	3.1482	1.85E-08	1.18E-06
sp|P02716|ACHD_MOUSE Acetylcholine receptor subunit delta OS=Mus musculus OX=10090 GN=Chrnd PE=2 SV=1//0	3.1136	6.45E-15	1.15E-12
sp|P01837|IGKC_MOUSE Immunoglobulin kappa constant OS=Mus musculus OX=10090 GN=Igkc PE=1 SV=2//2.11599e-64	3.0727	5.23E-20	1.55E-17
sp|Q6DFV6|FN3C1_MOUSE Fibronectin type III domain containing protein 3C1 OS=Mus musculus OX=10090 GN=Fndc3c1 PE=2 SV=1//0	3.0421	0.00013624	0.0032796
sp|Q9UGQ3|GTR6_HUMAN Solute carrier family 2, facilitated glucose transporter member 6 OS=Homo sapiens OX=9606 GN=SLC2A6 PE=1 SV=2//7.79545e-16	3.0172	1.35E-08	8.87E-07
sp|P52430|PON1_MOUSE Serum paraoxonase/arylesterase 1 OS=Mus musculus OX=10090 GN=Pon1 PE=1 SV=2//7.31539e-13	3.0104	2.84E-09	2.19E-07
sp|P10749|IL1B_MOUSE Interleukin-1 beta OS=Mus musculus OX=10090 GN=Il1b PE=1 SV=1//0	2.9764	8.00E-28	3.76E-25
sp|Q9D8U6|MCEM1_MOUSE Mast cell-expressed membrane protein 1 OS=Mus musculus OX=10090 GN=Mcemp1 PE=2 SV=1//8.11886e-130	2.9527	8.39E-05	0.0021581
sp|P34960|MMP12_MOUSE Macrophage metalloelastase OS=Mus musculus OX=10090 GN=Mmp12 PE=1 SV=3//9.58652e-15	2.9479	7.62E-37	5.71E-34
sp|Q8BGK2|ARHL1_MOUSE [Protein ADP-ribosylarginine] hydrolase-like protein 1 OS=Mus musculus OX=10090 GN=Adprhl1 PE=1 SV=1//0	2.938	3.00E-30	1.56E-27
sp|P01723|LV1A_MOUSE Ig lambda-1 chain V region OS=Mus musculus OX=10090 PE=1 SV=2//2.91917e-66	2.9312	0.00032763	0.0070829
sp|P47774|CCR7_MOUSE C-C chemokine receptor type 7 OS=Mus musculus OX=10090 GN=Ccr7 PE=1 SV=2//0	2.9253	2.64E-10	2.44E-08
sp|Q9Z0J7|GDF15_MOUSE Growth/differentiation factor 15 OS=Mus musculus OX=10090 GN=Gdf15 PE=1 SV=2//2.21971e-156	2.9188	3.45E-06	0.00013101
sp|P22777|PAI1_MOUSE Plasminogen activator inhibitor 1 OS=Mus musculus OX=10090 GN=Serpine1 PE=1 SV=1//0	2.9119	7.77E-19	2.03E-16
sp|Q9D5Z5|MSS51_MOUSE Putative protein MSS51 homolog, mitochondrial OS=Mus musculus OX=10090 GN=Mss51 PE=2 SV=1//0	2.8972	1.31E-12	1.79E-10
sp|Q3KNY0|IGFN1_MOUSE Immunoglobulin-like and fibronectin type III domain-containing protein 1 OS=Mus musculus OX=10090 GN=Igfn1 PE=1 SV=3//0	2.8903	5.14E-10	4.52E-08
sp|P18468|HB2I_MOUSE H-2 class II histocompatibility antigen, I-A beta chain OS=Mus musculus OX=10090 GN=H2-Eb1 PE=1 SV=1//8.06729e-115	2.8762	0.00011461	0.0028331
sp|P24288|BCAT1_MOUSE Branched-chain-amino-acid aminotransferase, cytosolic OS=Mus musculus OX=10090 GN=Bcat1 PE=1 SV=2//0	2.87	0.00011516	0.0028437
sp|Q03734|SPA3M_MOUSE Serine protease inhibitor A3M OS=Mus musculus OX=10090 GN=Serpina3m PE=1 SV=2//0	2.8682	1.27E-07	6.79E-06
sp|P25688|URIC_MOUSE Uricase OS=Mus musculus OX=10090 GN=Uox PE=1 SV=2//0	2.8553	0.00010125	0.0025368
sp|Q499E0|BRNP3_MOUSE BMP/retinoic acid-inducible neural-specific protein 3 OS=Mus musculus OX=10090 GN=Brinp3 PE=2 SV=2//0	2.8441	0.00055031	0.010957
sp|Q8K249|CLM2_MOUSE CMRF35-like molecule 2 OS=Mus musculus OX=10090 GN=Cd300e PE=2 SV=1//1.65909e-94	2.8397	0.00028082	0.0061938
-//-	2.8387	5.66E-10	4.88E-08
sp|Q62282|TAL2_MOUSE T-cell acute lymphocytic leukemia protein 2 homolog OS=Mus musculus OX=10090 GN=Tal2 PE=4 SV=1//2.94553e-59	2.8357	6.51E-09	4.64E-07
sp|Q60854|SPB6_MOUSE Serpin B6 OS=Mus musculus OX=10090 GN=Serpinb6 PE=1 SV=1//0	2.8326	0.00010039	0.0025179
sp|Q9JHX3|IL21R_MOUSE Interleukin-21 receptor OS=Mus musculus OX=10090 GN=Il21r PE=2 SV=1//0	2.8273	3.53E-11	3.71E-09
sp|Q810Q5|NMES1_MOUSE Normal mucosa of esophagus-specific gene 1 protein OS=Mus musculus OX=10090 GN=Nmes1 PE=1 SV=1//4.58626e-48	2.8066	2.19E-11	2.39E-09
sp|Q9R224|BEX1_MOUSE Protein BEX1 OS=Mus musculus OX=10090 GN=Bex1 PE=1 SV=2//6.1344e-72	2.7941	1.55E-11	1.79E-09
sp|P52927|HMGA2_MOUSE High mobility group protein HMGI-C OS=Mus musculus OX=10090 GN=Hmga2 PE=1 SV=1//6.22743e-28	2.7787	4.29E-07	2.04E-05
sp|O35474|EDIL3_MOUSE EGF-like repeat and discoidin I-like domain-containing protein 3 OS=Mus musculus OX=10090 GN=Edil3 PE=1 SV=2//0	2.7734	0.00016266	0.0038348

-//-	2.7607	0.0016036	0.026642
sp|Q9JKF4|CLC6A_MOUSE C-type lectin domain family 6 member A OS=Mus musculus OX=10090 GN=Clec6a PE=1 SV=1//4.25713e-15	2.7288	7.66E-20	2.18E-17
sp|P33766|FPR1_MOUSE fMet-Leu-Phe receptor OS=Mus musculus OX=10090 GN=Fpr1 PE=2 SV=1//0	2.6985	9.53E-06	0.00031527
sp|O08999|LTBP2_MOUSE Latent-transforming growth factor beta-binding protein 2 OS=Mus musculus OX=10090 GN=Ltbp2 PE=1 SV=2//0	2.6958	1.09E-13	1.64E-11
sp|Q9R0R4|APEL_MOUSE Apelin OS=Mus musculus OX=10090 GN=Apln PE=1 SV=1//5.06494e-23	2.6666	8.93E-21	2.81E-18
sp|Q9DCB4|ARP21_MOUSE cAMP-regulated phosphoprotein 21 OS=Mus musculus OX=10090 GN=Arpp21 PE=1 SV=2//1.45099e-131	2.6617	1.08E-18	2.71E-16
sp|O89109|KCNN4_MOUSE Intermediate conductance calcium-activated potassium channel protein 4 OS=Mus musculus OX=10090 GN=Kcnn4 PE=2 SV=1//0	2.6475	3.40E-09	2.58E-07
sp|P70325|TBX4_MOUSE T-box transcription factor TBX4 OS=Mus musculus OX=10090 GN=Tbx4 PE=1 SV=3//0	2.6375	1.64E-06	6.77E-05
sp|Q9Z2B1|I18RA_MOUSE Interleukin-18 receptor accessory protein OS=Mus musculus OX=10090 GN=Il18rap PE=2 SV=1//0	2.6348	2.88E-13	4.26E-11
sp|P20334|TNR9_MOUSE Tumor necrosis factor receptor superfamily member 9 OS=Mus musculus OX=10090 GN=Tnfrsf9 PE=1 SV=1//2.69073e-143	2.6309	2.57E-08	1.58E-06
sp|B5X0G2|MUP17_MOUSE Major urinary protein 17 OS=Mus musculus OX=10090 GN=Mup17 PE=2 SV=2//2.4009e-116	2.6282	4.54E-06	0.00016413
sp|Q3TES0|IQEC3_MOUSE IQ motif and SEC7 domain-containing protein 3 OS=Mus musculus OX=10090 GN=Iqsec3 PE=1 SV=1//0	2.6276	9.75E-10	8.11E-08
sp|O09161|CASQ2_MOUSE Calsequestrin-2 OS=Mus musculus OX=10090 GN=Casq2 PE=1 SV=3//0	2.6212	2.04E-30	1.09E-27
sp|P11588|MUP1_MOUSE Major urinary protein 1 OS=Mus musculus OX=10090 GN=Mup1 PE=1 SV=1//1.35863e-108	2.5856	2.57E-06	0.00010075
sp|Q99JB6|FOXP3_MOUSE Forkhead box protein P3 OS=Mus musculus OX=10090 GN=Foxp3 PE=1 SV=1//0	2.5765	0.00018804	0.0043561
sp|P10085|MYOD1_MOUSE Myoblast determination protein 1 OS=Mus musculus OX=10090 GN=Myod1 PE=1 SV=2//1.7919e-180	2.5674	9.24E-16	1.81E-13
sp|P33146|CAD15_MOUSE Cadherin-15 OS=Mus musculus OX=10090 GN=Cdh15 PE=1 SV=3//0	2.5619	1.58E-18	3.93E-16
sp|O88947|FA10_MOUSE Coagulation factor X OS=Mus musculus OX=10090 GN=F10 PE=1 SV=1//0	2.5592	2.08E-15	3.93E-13
sp|E9Q5R7|NAL12_MOUSE NACHT, LRR and PYD domains-containing protein 12 OS=Mus musculus OX=10090 GN=Nlrp12 PE=2 SV=1//0	2.5542	0.0032231	0.046593
sp|Q9ULN7|PNM8B_HUMAN Paraneoplastic antigen-like protein 8B OS=Homo sapiens OX=9606 GN=PNMA8B PE=2 SV=4//4.5667e-40	2.548	1.93E-10	1.83E-08
-//-	2.5473	1.88E-08	1.20E-06
-//-	2.5356	0.00012372	0.0030209
sp|Q99ME6|AT1B4_MOUSE Protein ATP1B4 OS=Mus musculus OX=10090 GN=Atp1b4 PE=1 SV=1//0	2.5348	1.13E-11	1.36E-09
sp|Q5T292|TM273_HUMAN Transmembrane protein 273 OS=Homo sapiens OX=9606 GN=TMEM273 PE=3 SV=1//3.30811e-27	2.5225	0.00075608	0.014238
sp|O35646|CAN6_MOUSE Calpain-6 OS=Mus musculus OX=10090 GN=Capn6 PE=1 SV=2//6.086e-26	2.504	2.17E-18	5.31E-16
sp|P35329|CD22_MOUSE B-cell receptor CD22 OS=Mus musculus OX=10090 GN=Cd22 PE=1 SV=1//0	2.4842	1.19E-05	0.00038445
sp|O70578|CCG1_MOUSE Voltage-dependent calcium channel gamma-1 subunit OS=Mus musculus OX=10090 GN=Cacng1 PE=1 SV=1//1.54124e-117	2.4813	8.15E-29	3.91E-26
sp|Q9JKB0|HCN1_RAT Potassium/sodium hyperpolarization-activated cyclic nucleotide-gated channel 1 OS=Rattus norvegicus OX=10116 GN=Hcn1 PE=2 SV=1//0	2.465	8.40E-05	0.0021581
sp|O35536|TFPI2_MOUSE Tissue factor pathway inhibitor 2 OS=Mus musculus OX=10090 GN=Tfpi2 PE=2 SV=1//1.07251e-147	2.4599	3.48E-09	2.63E-07
sp|P28862|MMP3_MOUSE Stromelysin-1 OS=Mus musculus OX=10090 GN=Mmp3 PE=2 SV=2//0	2.4524	1.52E-16	3.28E-14
sp|P48594|SPB4_HUMAN Serpin B4 OS=Homo sapiens OX=9606 GN=SERPINB4 PE=1 SV=2//4.46435e-138	2.4524	0.00046738	0.0095521
sp|P17257|F167B_MOUSE Protein FAM167B OS=Mus musculus OX=10090 GN=Fam167b PE=2 SV=2//1.97258e-67	2.436	1.10E-07	6.00E-06
sp|Q07440|B2LA1_MOUSE Bcl-2-related protein A1 OS=Mus musculus OX=10090 GN=Bcl2a1 PE=1 SV=1//2.47026e-123	2.435	0.00092749	0.016946
sp|P14234|FGR_MOUSE Tyrosine-protein kinase Fgr OS=Mus musculus OX=10090 GN=Fgr PE=1 SV=2//0	2.4274	1.70E-25	7.16E-23
sp|Q62240|KDM5D_MOUSE Lysine-specific demethylase 5D OS=Mus musculus OX=10090 GN=Kdm5d PE=2 SV=2//0	2.4247	2.43E-21	7.87E-19
sp|Q2UY11|COSA1_MOUSE Collagen alpha-1(XXVIII) chain OS=Mus musculus OX=10090 GN=Col28a1 PE=2 SV=1//0	2.4202	1.21E-05	0.00038916
sp|P04187|GRAB_MOUSE Granzyme B(G,H) OS=Mus musculus OX=10090 GN=Gzmb PE=1 SV=1//6.71966e-173	2.4076	8.79E-06	0.00029319
sp|Q01102|LYAM3_MOUSE P-selectin OS=Mus musculus OX=10090 GN=Selp PE=1 SV=1//0	2.4017	2.70E-05	0.00079154
sp|P51637|CAV3_MOUSE Caveolin-3 OS=Mus musculus OX=10090 GN=Cav3 PE=1 SV=1//1.41212e-104	2.3822	8.18E-24	3.11E-21
sp|Q7TSN2|CLM_MOUSE CMRF35-like molecule OS=Mus musculus OX=10090 GN=Clm PE=1 SV=1//3.73386e-134	2.3821	8.97E-19	2.29E-16
sp|O35348|COLQ_MOUSE Acetylcholinesterase collagenic tail peptide OS=Mus musculus OX=10090 GN=Colq PE=2 SV=2//8.71648e-151	2.3794	5.14E-09	3.76E-07

sp|Q61125|BKRB1_MOUSE B1 bradykinin receptor OS=Mus musculus OX=10090 GN=Bdkrb1 PE=2 SV=1//0	2.3588	0.0023998	0.036991
sp|P14719|ILRL1_MOUSE Interleukin-1 receptor-like 1 OS=Mus musculus OX=10090 GN=Il1rl1 PE=1 SV=2//0	2.3562	0.001607	0.026675
sp|Q9QYY7|ESM1_MOUSE Endothelial cell-specific molecule 1 OS=Mus musculus OX=10090 GN=Esm1 PE=2 SV=1//4.69611e-114	2.3422	9.99E-06	0.0003293
sp|Q9QZ85|IIGP1_MOUSE Interferon-inducible GTPase 1 OS=Mus musculus OX=10090 GN=Iigp1 PE=1 SV=2//1.09477e-105	2.3273	0.0015293	0.02567
sp|P97428|RGS16_MOUSE Regulator of G-protein signaling 16 OS=Mus musculus OX=10090 GN=Rgs16 PE=1 SV=1//5.60202e-122	2.3179	1.01E-13	1.53E-11
sp|P09690|ACHB_MOUSE Acetylcholine receptor subunit beta OS=Mus musculus OX=10090 GN=Chrnb1 PE=2 SV=1//0	2.3042	1.39E-20	4.33E-18
sp|Q9CZ16|T178A_MOUSE Transmembrane protein 178A OS=Mus musculus OX=10090 GN=Tmem178a PE=1 SV=3//8.67044e-179	2.293	0.00037316	0.007924
sp|P40223|CSF3R_MOUSE Granulocyte colony-stimulating factor receptor OS=Mus musculus OX=10090 GN=Csf3r PE=1 SV=2//0	2.2924	5.12E-19	1.36E-16
sp|Q6P5H2|NEST_MOUSE Nestin OS=Mus musculus OX=10090 GN=Nes PE=1 SV=1//0	2.2764	5.18E-24	2.03E-21
sp|Q9JHI0|MMP19_MOUSE Matrix metalloproteinase-19 OS=Mus musculus OX=10090 GN=Mmp19 PE=2 SV=1//0	2.2727	6.86E-08	3.92E-06
sp|Q9D7C9|NRK2_MOUSE Nicotinamide riboside kinase 2 OS=Mus musculus OX=10090 GN=Nmrk2 PE=1 SV=1//1.23222e-138	2.2703	2.02E-14	3.46E-12
sp|Q9QZ26|KCNE5_MOUSE Potassium voltage-gated channel subfamily E regulatory beta subunit 5 OS=Mus musculus OX=10090 GN=Kcne5 PE=2 SV=1//3.54064e-69	2.2552	0.0013331	0.022792
sp|Q9ESN4|C1QL3_MOUSE Complement C1q-like protein 3 OS=Mus musculus OX=10090 GN=C1ql3 PE=1 SV=1//5.08966e-121	2.2539	0.0012311	0.021353
sp|P97797|SHPS1_MOUSE Tyrosine-protein phosphatase non-receptor type substrate 1 OS=Mus musculus OX=10090 GN=Sirpa PE=1 SV=1//2.12218e-137	2.2482	0.00025133	0.0056316
sp|O88667|RAD_MOUSE GTP-binding protein RAD OS=Mus musculus OX=10090 GN=Rrad PE=1 SV=1//1.33725e-176	2.2387	2.48E-11	2.70E-09
sp|Q8R1C0|KCNC4_MOUSE Potassium voltage-gated channel subfamily C member 4 OS=Mus musculus OX=10090 GN=Kcnc4 PE=2 SV=1//0	2.2297	7.52E-14	1.18E-11
sp|Q80TR4|SLIT1_MOUSE Slit homolog 1 protein OS=Mus musculus OX=10090 GN=Slit1 PE=1 SV=2//3.11839e-36	2.2237	3.86E-06	0.00014482
sp|P50231|CCL5_RAT C-C motif chemokine 5 OS=Rattus norvegicus OX=10116 GN=Ccl5 PE=3 SV=1//5.04881e-19	2.2156	1.77E-07	9.21E-06
sp|P70402|MYBPH_MOUSE Myosin-binding protein H OS=Mus musculus OX=10090 GN=Mybph PE=2 SV=2//0	2.2086	1.61E-18	3.97E-16
sp|O88536|FPR2_MOUSE Formyl peptide receptor 2 OS=Mus musculus OX=10090 GN=Fpr2 PE=1 SV=1//0	2.203	0.0030869	0.045003
sp|Q9ET39|SLAF6_MOUSE SLAM family member 6 OS=Mus musculus OX=10090 GN=Slamf6 PE=1 SV=1//0	2.2019	0.0010754	0.019166
sp|Q9QXH4|ITAX_MOUSE Integrin alpha-X OS=Mus musculus OX=10090 GN=Itgax PE=1 SV=1//0	2.1961	3.54E-19	9.53E-17
sp|P10148|CCL2_MOUSE C-C motif chemokine 2 OS=Mus musculus OX=10090 GN=Ccl2 PE=1 SV=1//3.64167e-34	2.1946	8.08E-16	1.61E-13
sp|P97797|SHPS1_MOUSE Tyrosine-protein phosphatase non-receptor type substrate 1 OS=Mus musculus OX=10090 GN=Sirpa PE=1 SV=1//2.85799e-53	2.1893	0.00010444	0.0026136
sp|Q8BG84|LAIR1_MOUSE Leukocyte-associated immunoglobulin-like receptor 1 OS=Mus musculus OX=10090 GN=Lair1 PE=1 SV=1//7.91325e-143	2.1596	3.79E-07	1.83E-05
sp|P51682|CCR5_MOUSE C-C chemokine receptor type 5 OS=Mus musculus OX=10090 GN=Ccr5 PE=2 SV=3//0	2.1382	1.36E-09	1.10E-07
sp|Q7TNJ0|DCSTP_MOUSE Dendritic cell-specific transmembrane protein OS=Mus musculus OX=10090 GN=Dcstamp PE=1 SV=1//0	2.1369	5.03E-06	0.00017901
sp|P42228|STAT4_MOUSE Signal transducer and activator of transcription 4 OS=Mus musculus OX=10090 GN=Stat4 PE=1 SV=1//0	2.1368	0.0032069	0.046386
sp|Q3TZW7|BEX6_MOUSE Protein BEX6 OS=Mus musculus OX=10090 GN=Bex6 PE=3 SV=1//4.17464e-68	2.1366	0.0029743	0.04372
sp|Q9QUN7|TLR2_MOUSE Toll-like receptor 2 OS=Mus musculus OX=10090 GN=Tlr2 PE=1 SV=1//0	2.1363	5.48E-11	5.56E-09
sp|Q9WV08|APJ_MOUSE Apelin receptor OS=Mus musculus OX=10090 GN=Aplnr PE=1 SV=1//0	2.1236	3.97E-06	0.00014797
sp|P32304|5HT7R_MOUSE 5-hydroxytryptamine receptor 7 OS=Mus musculus OX=10090 GN=Htr7 PE=2 SV=2//0	2.1227	4.86E-05	0.0013315
sp|Q9TUQ3|CO7_PIG Complement component C7 OS=Sus scrofa OX=9823 GN=C7 PE=1 SV=1//0	2.1186	0.00072726	0.013814
sp|Q8K0J2|B3GN7_MOUSE UDP-GlcNAc:betaGal beta-1,3-N-acetylglucosaminyltransferase 7 OS=Mus musculus OX=10090 GN=B3gnt7 PE=2 SV=2//0	2.1137	0.0019776	0.031601
sp|Q5FWI2|SCTR_MOUSE Secretin receptor OS=Mus musculus OX=10090 GN=Sctr PE=2 SV=1//0	2.1084	0.00062753	0.012122
sp|Q3U435|MMP25_MOUSE Matrix metalloproteinase-25 OS=Mus musculus OX=10090 GN=Mmp25 PE=2 SV=1//0	2.1052	1.67E-06	6.83E-05
sp|Q61006|MUSK_MOUSE Muscle, skeletal receptor tyrosine-protein kinase OS=Mus musculus OX=10090 GN=Musk PE=1 SV=1//0	2.1029	1.89E-06	7.59E-05
sp|P33680|GUC2A_MOUSE Guanylin OS=Mus musculus OX=10090 GN=Guca2a PE=2 SV=1//9.60418e-60	2.0956	0.00047812	0.0097219
sp|Q8CI43|MYL6B_MOUSE Myosin light chain 6B OS=Mus musculus OX=10090 GN=Myl6b PE=1 SV=1//2.51761e-116	2.0689	2.95E-06	0.00011435
sp|Q6DIB5|MEG10_MOUSE Multiple epidermal growth factor-like domains protein 10 OS=Mus musculus OX=10090 GN=Megf10 PE=1 SV=1//0	2.0677	4.28E-10	3.81E-08
sp|O54775|WISP1_MOUSE WNT1-inducible-signaling pathway protein 1 OS=Mus musculus OX=10090 GN=Wisp1 PE=2 SV=1//0	2.0634	1.44E-05	0.00045471

sp|Q8CD54|PIEZ2_MOUSE Piezo-type mechanosensitive ion channel component 2 OS=Mus musculus OX=10090 GN=Piezo2 PE=1 SV=2//0	2.0566	0.00011671	0.002873
sp|P83626|TNR26_MOUSE Tumor necrosis factor receptor superfamily member 26 OS=Mus musculus OX=10090 GN=Tnfrsf26 PE=2 SV=1//1.85225e-101	2.0433	1.75E-05	0.00054483
sp|O35608|ANGP2_MOUSE Angiopoietin-2 OS=Mus musculus OX=10090 GN=Angpt2 PE=2 SV=2//0	2.0408	1.20E-12	1.66E-10
sp|Q8MJV1|MYH2_HORSE Myosin-2 OS=Equus caballus OX=9796 GN=MYH2 PE=2 SV=1//3.26823e-19	2.0397	0.0033023	0.047651
sp|Q03366|CCL7_MOUSE C-C motif chemokine 7 OS=Mus musculus OX=10090 GN=Ccl7 PE=3 SV=1//1.82097e-53	2.0263	4.23E-10	3.79E-08
sp|Q01231|CXA5_MOUSE Gap junction alpha-5 protein OS=Mus musculus OX=10090 GN=Gja5 PE=2 SV=2//0	2.01	7.14E-09	5.02E-07
sp|Q0VBP7|SHSL1_MOUSE Protein shisa-like-1 OS=Mus musculus OX=10090 GN=Shisal1 PE=2 SV=1//2.27727e-101	2.0071	0.00049456	0.0099968
sp|Q01338|ADA2A_MOUSE Alpha-2A adrenergic receptor OS=Mus musculus OX=10090 GN=Adra2a PE=1 SV=1//0	2.0013	5.96E-12	7.44E-10
**Blast swiss prot (DOWNREGULATED, >2FC)**	**log2FoldChange**	**pval**	**padj**
sp|Q8VCW2|K1C25_MOUSE Keratin, type I cytoskeletal 25 OS=Mus musculus OX=10090 GN=Krt25 PE=1 SV=1//0	-12.4	5.30E-16	1.09E-13
sp|Q8VCW2|K1C25_MOUSE Keratin, type I cytoskeletal 25 OS=Mus musculus OX=10090 GN=Krt25 PE=1 SV=1//0	-12.4	5.30E-16	1.09E-13
sp|Q9R0H5|K2C71_MOUSE Keratin, type II cytoskeletal 71 OS=Mus musculus OX=10090 GN=Krt71 PE=1 SV=1//0	-11.366	8.70E-09	5.95E-07
sp|Q9R0H5|K2C71_MOUSE Keratin, type II cytoskeletal 71 OS=Mus musculus OX=10090 GN=Krt71 PE=1 SV=1//0	-11.366	8.70E-09	5.95E-07
sp|P40936|INMT_MOUSE Indolethylamine N-methyltransferase OS=Mus musculus OX=10090 GN=Inmt PE=1 SV=1//6.87575e-173	-10.554	2.37E-07	1.19E-05
sp|P40936|INMT_MOUSE Indolethylamine N-methyltransferase OS=Mus musculus OX=10090 GN=Inmt PE=1 SV=1//6.87575e-173	-10.554	2.37E-07	1.19E-05
sp|Q9Z320|K1C27_MOUSE Keratin, type I cytoskeletal 27 OS=Mus musculus OX=10090 GN=Krt27 PE=1 SV=1//0	-10.237	5.86E-05	0.0015758
sp|Q497I4|KRT35_MOUSE Keratin, type I cuticular Ha5 OS=Mus musculus OX=10090 GN=Krt35 PE=1 SV=1//2.69816e-73	-10.169	1.71E-120	1.36E-116
sp|Q6NXH9|K2C73_MOUSE Keratin, type II cytoskeletal 73 OS=Mus musculus OX=10090 GN=Krt73 PE=1 SV=1//0	-9.7242	1.35E-39	1.25E-36
sp|Q9UBG3|CRNN_HUMAN Cornulin OS=Homo sapiens OX=9606 GN=CRNN PE=1 SV=1//5.50769e-138	-8.7129	0.0030774	0.044893
sp|Q6IMF0|KRT87_MOUSE Keratin, type II cuticular 87 OS=Mus musculus OX=10090 GN=Krt87 PE=2 SV=2//0	-8.4862	0.0028568	0.042241
sp|Q8R2I0|FOXE1_MOUSE Forkhead box protein E1 OS=Mus musculus OX=10090 GN=Foxe1 PE=1 SV=2//5.24568e-96	-7.9173	6.86E-45	7.83E-42
sp|Q91VA3|CAN8_MOUSE Calpain-8 OS=Mus musculus OX=10090 GN=Capn8 PE=1 SV=1//0	-7.6391	0.0010093	0.018218
sp|O95932|TGM3L_HUMAN Protein-glutamine gamma-glutamyltransferase 6 OS=Homo sapiens OX=9606 GN=TGM6 PE=1 SV=3//0	-7.5021	0.0020439	0.032465
sp|Q9Z184|PADI3_MOUSE Protein-arginine deiminase type-3 OS=Mus musculus OX=10090 GN=Padi3 PE=1 SV=2//0	-7.5007	0.00077665	0.014556
sp|Q9QXD6|F16P1_MOUSE Fructose-1,6-bisphosphatase 1 OS=Mus musculus OX=10090 GN=Fbp1 PE=1 SV=3//0	-6.9914	2.63E-36	1.85E-33
sp|A6BLY7|K1C28_MOUSE Keratin, type I cytoskeletal 28 OS=Mus musculus OX=10090 GN=Krt28 PE=1 SV=1//0	-6.6375	2.80E-09	2.17E-07
sp|Q8C196|CPSM_MOUSE Carbamoyl-phosphate synthase [ammonia], mitochondrial OS=Mus musculus OX=10090 GN=Cps1 PE=1 SV=2//0	-6.4308	4.48E-14	7.21E-12
sp|Q7TMD7|DSG4_MOUSE Desmoglein-4 OS=Mus musculus OX=10090 GN=Dsg4 PE=1 SV=1//0	-5.9189	3.91E-05	0.0011103
sp|Q76K27|SIAT2_MOUSE Beta-galactoside alpha-2,6-sialyltransferase 2 OS=Mus musculus OX=10090 GN=St6gal2 PE=2 SV=2//0	-5.6431	5.08E-08	2.95E-06
sp|P35347|CRFR1_MOUSE Corticotropin-releasing factor receptor 1 OS=Mus musculus OX=10090 GN=Crhr1 PE=1 SV=1//0	-5.5398	1.28E-11	1.51E-09
sp|P51661|DHI2_MOUSE Corticosteroid 11-beta-dehydrogenase isozyme 2 OS=Mus musculus OX=10090 GN=Hsd11b2 PE=1 SV=2//0	-5.5206	1.89E-25	7.82E-23
-//-	-5.4947	3.25E-127	7.78E-123
sp|Q60928|GGT1_MOUSE Glutathione hydrolase 1 proenzyme OS=Mus musculus OX=10090 GN=Ggt1 PE=1 SV=1//0	-5.4461	1.46E-25	6.26E-23
sp|Q8BUM6|F163B_MOUSE Protein FAM163B OS=Mus musculus OX=10090 GN=Fam163b PE=1 SV=1//1.14193e-100	-5.4396	2.06E-12	2.72E-10
sp|Q9Z319|CORIN_MOUSE Atrial natriuretic peptide-converting enzyme OS=Mus musculus OX=10090 GN=Corin PE=2 SV=2//0	-5.3594	1.36E-12	1.84E-10
sp|Q62226|SHH_MOUSE Sonic hedgehog protein OS=Mus musculus OX=10090 GN=Shh PE=1 SV=2//0	-5.2446	2.12E-41	2.04E-38
sp|Q62052|P_MOUSE P protein OS=Mus musculus OX=10090 GN=Oca2 PE=1 SV=1//1.01015e-58	-5.0253	1.80E-11	2.04E-09
sp|O54908|DKK1_MOUSE Dickkopf-related protein 1 OS=Mus musculus OX=10090 GN=Dkk1 PE=1 SV=2//5.1567e-169	-5.0009	0.0010795	0.019224
sp|P70436|DLX4_MOUSE Homeobox protein DLX-4 OS=Mus musculus OX=10090 GN=Dlx4 PE=2 SV=2//1.00402e-131	-4.8657	7.30E-13	1.04E-10
sp|P58355|S45A2_MOUSE Membrane-associated transporter protein OS=Mus musculus OX=10090 GN=Slc45a2 PE=1 SV=1//0	-4.6854	8.23E-21	2.63E-18
sp|Q8VI67|SP7_MOUSE Transcription factor Sp7 OS=Mus musculus OX=10090 GN=Sp7 PE=1 SV=1//0	-4.6808	1.08E-08	7.23E-07

sp|A2BDP1|F155B_MOUSE Transmembrane protein FAM155B OS=Mus musculus OX=10090 GN=Fam155b PE=3 SV=1//0	-4.5584	1.20E-05	0.00038446
sp|P10610|CP2G1_RAT Cytochrome P450 2G1 OS=Rattus norvegicus OX=10116 GN=Cyp2g1 PE=2 SV=1//0	-4.4724	2.24E-27	1.03E-24
sp|P30875|SSR2_MOUSE Somatostatin receptor type 2 OS=Mus musculus OX=10090 GN=Sstr2 PE=2 SV=1//0	-4.397	4.50E-07	2.14E-05
sp|Q61282|PGCA_MOUSE Aggrecan core protein OS=Mus musculus OX=10090 GN=Acan PE=1 SV=2//0	-4.333	1.84E-30	1.02E-27
sp|P11344|TYRO_MOUSE Tyrosinase OS=Mus musculus OX=10090 GN=Tyr PE=1 SV=3//0	-4.3191	7.99E-33	4.79E-30
sp|O54792|HES2_MOUSE Transcription factor HES-2 OS=Mus musculus OX=10090 GN=Hes2 PE=2 SV=2//5.59769e-72	-4.2989	4.82E-13	6.91E-11
sp|Q80VJ8|KASH5_MOUSE Protein KASH5 OS=Mus musculus OX=10090 GN=Ccdc155 PE=1 SV=3//0	-4.1754	1.06E-05	0.00034808
sp|Q9EQ21|HEPC_MOUSE Hepcidin OS=Mus musculus OX=10090 GN=Hamp PE=2 SV=1//1.46704e-41	-4.1561	0.0022677	0.035364
sp|Q9D428|GOG7B_MOUSE Golgin subfamily A member 7B OS=Mus musculus OX=10090 GN=GOLGA7B PE=1 SV=1//3.98989e-93	-4.0825	9.69E-46	1.16E-42
sp|Q8CGQ8|NCKX4_MOUSE Sodium/potassium/calcium exchanger 4 OS=Mus musculus OX=10090 GN=Slc24a4 PE=1 SV=2//4.60323e-23	-4.0477	1.75E-08	1.13E-06
sp|P34821|BMP8A_MOUSE Bone morphogenetic protein 8A OS=Mus musculus OX=10090 GN=Bmp8a PE=2 SV=1//0	-3.9505	5.33E-05	0.0014413
sp|P03966|MYCN_MOUSE N-myc proto-oncogene protein OS=Mus musculus OX=10090 GN=Mycn PE=2 SV=2//0	-3.8484	4.17E-26	1.85E-23
sp|O70624|MYOC_MOUSE Myocilin OS=Mus musculus OX=10090 GN=Myoc PE=1 SV=1//0	-3.8353	5.73E-23	2.05E-20
sp|O35595|PTC2_MOUSE Protein patched homolog 2 OS=Mus musculus OX=10090 GN=Ptch2 PE=2 SV=2//0	-3.7865	1.28E-54	2.19E-51
sp|P15313|VATB1_HUMAN V-type proton ATPase subunit B, kidney isoform OS=Homo sapiens OX=9606 GN=ATP6V1B1 PE=1 SV=3//0	-3.7781	5.35E-23	1.94E-20
sp|Q01727|MSHR_MOUSE Melanocyte-stimulating hormone receptor OS=Mus musculus OX=10090 GN=Mc1r PE=2 SV=2//8.10263e-180	-3.7612	2.33E-12	3.03E-10
sp|P40764|DLX2_MOUSE Homeobox protein DLX-2 OS=Mus musculus OX=10090 GN=Dlx2 PE=1 SV=1//6.67834e-160	-3.7502	2.69E-23	9.93E-21
sp|Q91ZZ3|SYUB_MOUSE Beta-synuclein OS=Mus musculus OX=10090 GN=Sncb PE=1 SV=1//8.13437e-54	-3.7437	2.05E-06	8.18E-05
sp|Q8BVG5|GLT14_MOUSE Polypeptide N-acetylgalactosaminyltransferase 14 OS=Mus musculus OX=10090 GN=Galnt14 PE=2 SV=2//0	-3.7061	2.58E-10	2.39E-08
sp|Q8BHB9|CLIC6_MOUSE Chloride intracellular channel protein 6 OS=Mus musculus OX=10090 GN=Clic6 PE=1 SV=1//0	-3.5595	3.26E-06	0.00012422
sp|Q2TV84|TRPM1_MOUSE Transient receptor potential cation channel subfamily M member 1 OS=Mus musculus OX=10090 GN=Trpm1 PE=2 SV=2//0	-3.5401	9.80E-42	9.78E-39
sp|Q64317|DLX1_MOUSE Homeobox protein DLX-1 OS=Mus musculus OX=10090 GN=Dlx1 PE=1 SV=1//5.05544e-132	-3.5185	9.95E-14	1.52E-11
sp|P70259|GP143_MOUSE G-protein coupled receptor 143 OS=Mus musculus OX=10090 GN=Gpr143 PE=2 SV=1//0	-3.4708	0.00042797	0.0089297
sp|Q8C261|NCKX5_MOUSE Sodium/potassium/calcium exchanger 5 OS=Mus musculus OX=10090 GN=Slc24a5 PE=2 SV=1//0	-3.4162	1.13E-12	1.58E-10
sp|P40749|SYT4_MOUSE Synaptotagmin-4 OS=Mus musculus OX=10090 GN=Syt4 PE=1 SV=2//0	-3.2975	3.37E-20	1.01E-17
sp|P70436|DLX4_MOUSE Homeobox protein DLX-4 OS=Mus musculus OX=10090 GN=Dlx4 PE=2 SV=2//2.33109e-07	-3.2939	3.90E-06	0.0001455
sp|O88507|CNTFR_MOUSE Ciliary neurotrophic factor receptor subunit alpha OS=Mus musculus OX=10090 GN=Cntfr PE=1 SV=2//0	-3.2857	1.00E-36	7.28E-34
sp|Q99388|CSPRS_MOUSE Component of Sp100-rs OS=Mus musculus OX=10090 GN=Csprs PE=2 SV=1//7.96605e-13	-3.2758	0.0031774	0.046079
sp|P20236|GBRA3_RAT Gamma-aminobutyric acid receptor subunit alpha-3 OS=Rattus norvegicus OX=10116 GN=Gabra3 PE=1 SV=2//0	-3.2748	0.0025838	0.038888
sp|Q9ET66|PI16_MOUSE Peptidase inhibitor 16 OS=Mus musculus OX=10090 GN=Pi16 PE=1 SV=1//2.17787e-125	-3.2602	1.54E-35	1.02E-32
sp|Q8VCF5|TSN10_MOUSE Tetraspanin-10 OS=Mus musculus OX=10090 GN=Tspan10 PE=1 SV=1//0	-3.255	1.78E-08	1.14E-06
sp|P28651|CAH8_MOUSE Carbonic anhydrase-related protein OS=Mus musculus OX=10090 GN=Ca8 PE=1 SV=5//0	-3.2479	0.00019053	0.004401
sp|Q8CFZ4|GPC3_MOUSE Glypican-3 OS=Mus musculus OX=10090 GN=Gpc3 PE=2 SV=1//0	-3.2074	1.01E-49	1.35E-46
sp|O35137|ALX4_MOUSE Homeobox protein aristaless-like 4 OS=Mus musculus OX=10090 GN=Alx4 PE=1 SV=1//2.37786e-47	-3.1976	4.57E-27	2.07E-24
sp|P27782|LEF1_MOUSE Lymphoid enhancer-binding factor 1 OS=Mus musculus OX=10090 GN=Lef1 PE=1 SV=1//1.05632e-63	-3.1613	1.46E-33	9.22E-31
sp|Q9QXF8|GNMT_MOUSE Glycine N-methyltransferase OS=Mus musculus OX=10090 GN=Gnmt PE=1 SV=3//0	-3.1529	1.31E-08	8.67E-07
sp|G3X982|AOXC_MOUSE Aldehyde oxidase 3 OS=Mus musculus OX=10090 GN=Aox3 PE=1 SV=1//0	-3.1057	5.23E-06	0.00018555
sp|Q64205|DLX3_MOUSE Homeobox protein DLX-3 OS=Mus musculus OX=10090 GN=Dlx3 PE=2 SV=1//2.60838e-166	-3.103	7.91E-45	8.61E-42
-//-	-3.0857	0.0034427	0.049261
-//-	-3.0582	0.00084659	0.015671
sp|Q95JH6|AK1C1_MACFU Aldo-keto reductase family 1 member C1 homolog OS=Macaca fuscata fuscata OX=9543 GN=AKR1C1 PE=2 SV=1//1.12227e-175	-3.0533	0.00033794	0.0072729

sp|P06876|MYB_MOUSE Transcriptional activator Myb OS=Mus musculus OX=10090 GN=Myb PE=1 SV=2//1.94915e-152	-3.0259	5.73E-16	1.16E-13
sp|Q03358|MSX2_MOUSE Homeobox protein MSX-2 OS=Mus musculus OX=10090 GN=Msx2 PE=1 SV=2//7.97144e-166	-3.0186	1.54E-31	9.00E-29
sp|Q14B46|RTKN2_MOUSE Rhotekin-2 OS=Mus musculus OX=10090 GN=Rtkn2 PE=1 SV=2//0	-3.0128	1.10E-05	0.00036029
sp|P48299|EDN3_MOUSE Endothelin-3 OS=Mus musculus OX=10090 GN=Edn3 PE=2 SV=1//6.30577e-134	-3.008	0.00058163	0.011419
sp|P47806|GLI1_MOUSE Zinc finger protein GLI1 OS=Mus musculus OX=10090 GN=Gli1 PE=1 SV=4//0	-2.9906	2.01E-38	1.66E-35
sp|Q6XD76|ASCL4_HUMAN Achaete-scute homolog 4 OS=Homo sapiens OX=9606 GN=ASCL4 PE=1 SV=1//3.78484e-31	-2.9773	0.00010549	0.002632
sp|Q9EQC9|CXXC4_RAT CXXC-type zinc finger protein 4 OS=Rattus norvegicus OX=10116 GN=Cxxc4 PE=1 SV=1//2.88336e-105	-2.9529	5.12E-05	0.0013892
sp|O35084|CP27B_MOUSE 25-hydroxyvitamin D-1 alpha hydroxylase, mitochondrial OS=Mus musculus OX=10090 GN=Cyp27b1 PE=1 SV=2//5.04512e-31	-2.939	4.17E-12	5.35E-10
sp|P13297|MSX1_MOUSE Homeobox protein MSX-1 OS=Mus musculus OX=10090 GN=Msx1 PE=1 SV=4//1.01829e-138	-2.9383	0.00083335	0.015498
sp|Q66PY1|SCUB3_MOUSE Signal peptide, CUB and EGF-like domain-containing protein 3 OS=Mus musculus OX=10090 GN=Scube3 PE=1 SV=1//0	-2.9259	3.22E-30	1.64E-27
sp|P07147|TYRP1_MOUSE 5,6-dihydroxyindole-2-carboxylic acid oxidase OS=Mus musculus OX=10090 GN=Tyrp1 PE=1 SV=1//0	-2.9251	1.96E-30	1.07E-27
sp|Q8CGD2|CRLD1_MOUSE Cysteine-rich secretory protein LCCL domain-containing 1 OS=Mus musculus OX=10090 GN=Crispld1 PE=2 SV=1//0	-2.9164	2.61E-22	9.20E-20
sp|Q60696|PMEL_MOUSE Melanocyte protein PMEL OS=Mus musculus OX=10090 GN=Pmel PE=1 SV=1//0	-2.8841	1.31E-34	8.45E-32
sp|P50207|HXC13_MOUSE Homeobox protein Hox-C13 OS=Mus musculus OX=10090 GN=Hoxc13 PE=2 SV=2//1.78203e-167	-2.8559	4.78E-30	2.34E-27
sp|Q61979|NNAT_MOUSE Neuronatin OS=Mus musculus OX=10090 GN=Nnat PE=2 SV=2//9.58926e-22	-2.8172	1.22E-17	2.87E-15
sp|Q6Q4G3|AMPQ_HUMAN Aminopeptidase Q OS=Homo sapiens OX=9606 GN=LVRN PE=1 SV=4//0	-2.7979	4.56E-08	2.66E-06
sp|Q9QYM9|TEFF2_MOUSE Tomoregulin-2 OS=Mus musculus OX=10090 GN=Tmeff2 PE=2 SV=1//0	-2.7818	6.94E-14	1.10E-11
sp|Q9D2U9|H2B3A_MOUSE Histone H2B type 3-A OS=Mus musculus OX=10090 GN=Hist3h2ba PE=1 SV=3//1.15894e-74	-2.7751	8.13E-05	0.0021048
sp|Q9R187|EDAR_MOUSE Tumor necrosis factor receptor superfamily member EDAR OS=Mus musculus OX=10090 GN=Edar PE=1 SV=1//0	-2.7579	2.88E-09	2.21E-07
sp|P48614|WN10B_MOUSE Protein Wnt-10b OS=Mus musculus OX=10090 GN=Wnt10b PE=2 SV=1//0	-2.7448	3.72E-17	8.40E-15
sp|Q61115|PTC1_MOUSE Protein patched homolog 1 OS=Mus musculus OX=10090 GN=Ptch1 PE=1 SV=1//0	-2.74	7.61E-37	5.71E-34
sp|Q7TN16|HHIP_MOUSE Hedgehog-interacting protein OS=Mus musculus OX=10090 GN=Hhip PE=1 SV=2//0	-2.7086	3.60E-11	3.74E-09
sp|Q8K441|ABCA6_MOUSE ATP-binding cassette sub-family A member 6 OS=Mus musculus OX=10090 GN=Abca6 PE=1 SV=2//0	-2.6904	1.20E-09	9.81E-08
sp|Q5BJU5|CNIH2_RAT Protein cornichon homolog 2 OS=Rattus norvegicus OX=10116 GN=Cnih2 PE=1 SV=1//1.81549e-95	-2.6876	8.22E-10	6.96E-08
sp|Q9JKN6|NOVA1_MOUSE RNA-binding protein Nova-1 OS=Mus musculus OX=10090 GN=Nova1 PE=1 SV=2//9.36133e-08	-2.6841	0.00011062	0.0027544
sp|P29812|TYRP2_MOUSE L-dopachrome tautomerase OS=Mus musculus OX=10090 GN=Dct PE=1 SV=2//0	-2.6642	3.54E-37	2.82E-34
sp|Q61474|MSI1H_MOUSE RNA-binding protein Musashi homolog 1 OS=Mus musculus OX=10090 GN=Msi1 PE=1 SV=1//0	-2.6638	5.79E-10	4.97E-08
sp|Q9ESL8|FGF16_MOUSE Fibroblast growth factor 16 OS=Mus musculus OX=10090 GN=Fgf16 PE=2 SV=2//5.36015e-145	-2.6638	0.00020734	0.0047435
sp|P13346|FOSB_MOUSE Protein fosB OS=Mus musculus OX=10090 GN=Fosb PE=2 SV=1//4.5623e-144	-2.6405	3.80E-13	5.49E-11
sp|P97303|BACH2_MOUSE Transcription regulator protein BACH2 OS=Mus musculus OX=10090 GN=Bach2 PE=1 SV=3//0	-2.6271	3.83E-30	1.91E-27
sp|Q5IS61|OPCM_PANTR Opioid-binding protein/cell adhesion molecule OS=Pan troglodytes OX=9598 GN=OPCML PE=2 SV=1//0	-2.6156	4.36E-09	3.24E-07
sp|Q9JJF0|NP1L5_MOUSE Nucleosome assembly protein 1-like 5 OS=Mus musculus OX=10090 GN=Nap1l5 PE=1 SV=1//1.56389e-66	-2.6085	0.001462	0.024678
sp|Q8CJ91|C209B_MOUSE CD209 antigen-like protein B OS=Mus musculus OX=10090 GN=Cd209b PE=1 SV=2//0	-2.5314	3.64E-13	5.28E-11
sp|P31428|DPEP1_MOUSE Dipeptidase 1 OS=Mus musculus OX=10090 GN=Dpep1 PE=1 SV=2//0	-2.5304	3.04E-31	1.74E-28
sp|Q2VLH6|C163A_MOUSE Scavenger receptor cysteine-rich type 1 protein M130 OS=Mus musculus OX=10090 GN=Cd163 PE=1 SV=2//0	-2.5043	1.61E-23	6.03E-21
sp|Q4VC17|ATS18_MOUSE A disintegrin and metalloproteinase with thrombospondin motifs 18 OS=Mus musculus OX=10090 GN=Adamts18 PE=2 SV=2//0	-2.496	3.61E-11	3.74E-09
sp|Q7TSH9|ZN184_MOUSE Zinc finger protein 184 OS=Mus musculus OX=10090 GN=Zfp184 PE=2 SV=1//0	-2.4946	0.0013194	0.022574
sp|P22389|EDN2_MOUSE Endothelin-2 OS=Mus musculus OX=10090 GN=Edn2 PE=2 SV=3//5.92089e-93	-2.4872	1.03E-06	4.47E-05
sp|Q8BWQ5|DCLK3_MOUSE Serine/threonine-protein kinase DCLK3 OS=Mus musculus OX=10090 GN=Dclk3 PE=1 SV=2//0	-2.4753	1.46E-11	1.70E-09
sp|Q6PAL1|DRAXI_MOUSE Draxin OS=Mus musculus OX=10090 GN=Draxin PE=1 SV=2//0	-2.4322	3.65E-06	0.00013785
sp|Q9QUQ5|TRPC4_MOUSE Short transient receptor potential channel 4 OS=Mus musculus OX=10090 GN=Trpc4 PE=1 SV=1//0	-2.4294	0.0012067	0.021018

sp|Q8BS03|PI15_MOUSE Peptidase inhibitor 15 OS=Mus musculus OX=10090 GN=Pi15 PE=2 SV=2//6.17885e-167	-2.4218	4.89E-05	0.0013379
sp|Q8BLR2|CPNE4_MOUSE Copine-4 OS=Mus musculus OX=10090 GN=Cpne4 PE=1 SV=1//0	-2.4107	0.00029649	0.0064445
sp|Q3UWA6|GUC2C_MOUSE Heat-stable enterotoxin receptor OS=Mus musculus OX=10090 GN=Gucy2c PE=2 SV=1//0	-2.4103	0.001841	0.029735
sp|Q8C6S9|CFA54_MOUSE Cilia- and flagella-associated protein 54 OS=Mus musculus OX=10090 GN=Cfap54 PE=2 SV=4//0	-2.3994	0.0011408	0.020093
sp|Q80T19|HEPC2_MOUSE Hepcidin-2 OS=Mus musculus OX=10090 GN=Hamp2 PE=2 SV=1//3.27113e-34	-2.3909	0.001227	0.021297
sp|O08691|ARGI2_MOUSE Arginase-2, mitochondrial OS=Mus musculus OX=10090 GN=Arg2 PE=1 SV=1//0	-2.3722	3.16E-15	5.91E-13
sp|O88472|TNR17_MOUSE Tumor necrosis factor receptor superfamily member 17 OS=Mus musculus OX=10090 GN=Tnfrsf17 PE=2 SV=1//1.64578e-134	-2.3364	0.0033657	0.048419
sp|P16406|AMPE_MOUSE Glutamyl aminopeptidase OS=Mus musculus OX=10090 GN=Enpep PE=1 SV=1//0	-2.3167	0.0031357	0.045577
-//-	-2.3132	0.00014999	0.0035643
sp|P41588|VIPR2_MOUSE Vasoactive intestinal polypeptide receptor 2 OS=Mus musculus OX=10090 GN=Vipr2 PE=1 SV=1//0	-2.3095	0.0021869	0.034282
sp|Q9JJV4|CCG4_MOUSE Voltage-dependent calcium channel gamma-4 subunit OS=Mus musculus OX=10090 GN=Cacng4 PE=1 SV=1//0	-2.3079	0.0001265	0.0030817
sp|Q8BGK9|CC018_MOUSE Uncharacterized protein C3orf18 homolog OS=Mus musculus OX=10090 PE=2 SV=1//2.66788e-79	-2.2947	8.33E-09	5.74E-07
sp|P97326|CADH6_MOUSE Cadherin-6 OS=Mus musculus OX=10090 GN=Cdh6 PE=1 SV=2//0	-2.2765	6.58E-06	0.00022848
sp|Q3V1H9|SAMD5_MOUSE Sterile alpha motif domain-containing protein 5 OS=Mus musculus OX=10090 GN=Samd5 PE=2 SV=1//1.77977e-101	-2.2718	1.12E-11	1.35E-09
-//-	-2.2174	0.0017442	0.028499
sp|Q8K078|SO4A1_MOUSE Solute carrier organic anion transporter family member 4A1 OS=Mus musculus OX=10090 GN=Slco4a1 PE=1 SV=2//0	-2.2132	9.20E-15	1.61E-12
sp|O88866|HUNK_MOUSE Hormonally up-regulated neu tumor-associated kinase OS=Mus musculus OX=10090 GN=Hunk PE=2 SV=1//0	-2.2082	8.19E-11	8.04E-09
sp|Q3TY65|ICA1L_MOUSE Islet cell autoantigen 1-like protein OS=Mus musculus OX=10090 GN=Ica1l PE=1 SV=1//0	-2.1931	0.00043418	0.0090356
sp|Q6T4R5|NHS_HUMAN Nance-Horan syndrome protein OS=Homo sapiens OX=9606 GN=NHS PE=1 SV=2//0	-2.188	2.17E-14	3.69E-12
sp|P48356|LEPR_MOUSE Leptin receptor OS=Mus musculus OX=10090 GN=Lepr PE=1 SV=1//0	-2.1744	5.60E-24	2.16E-21
sp|P47867|SCG3_MOUSE Secretogranin-3 OS=Mus musculus OX=10090 GN=Scg3 PE=1 SV=1//0	-2.1728	1.16E-05	0.00037463
sp|Q16655|MAR1_HUMAN Melanoma antigen recognized by T-cells 1 OS=Homo sapiens OX=9606 GN=MLANA PE=1 SV=1//8.6264e-32	-2.1674	1.45E-11	1.69E-09
sp|P43025|TETN_MOUSE Tetranectin OS=Mus musculus OX=10090 GN=Clec3b PE=1 SV=2//4.44072e-136	-2.1531	1.55E-12	2.06E-10
sp|Q8K299|SCAR5_MOUSE Scavenger receptor class A member 5 OS=Mus musculus OX=10090 GN=Scara5 PE=1 SV=1//0	-2.1257	1.16E-25	5.07E-23
sp|Q8CIB9|ESCO2_MOUSE N-acetyltransferase ESCO2 OS=Mus musculus OX=10090 GN=Esco2 PE=2 SV=3//0	-2.1008	1.14E-14	1.98E-12
sp|P22005|PENK_MOUSE Proenkephalin-A OS=Mus musculus OX=10090 GN=Penk PE=1 SV=2//0	-2.0978	8.44E-17	1.85E-14
sp|Q9Z183|PADI4_MOUSE Protein-arginine deiminase type-4 OS=Mus musculus OX=10090 GN=Padi4 PE=2 SV=3//1.84386e-16	-2.094	2.50E-11	2.71E-09
sp|P41236|IPP2_HUMAN Protein phosphatase inhibitor 2 OS=Homo sapiens OX=9606 GN=PPP1R2 PE=1 SV=2//2.97068e-21	-2.0815	1.75E-05	0.00054492
sp|Q9CXH7|SGO1_MOUSE Shugoshin 1 OS=Mus musculus OX=10090 GN=Sgo1 PE=2 SV=1//0	-2.0783	8.99E-08	5.01E-06
sp|Q6ZPS2|CRNS1_MOUSE Carnosine synthase 1 OS=Mus musculus OX=10090 GN=Carns1 PE=1 SV=2//2.88682e-17	-2.0645	3.15E-05	0.00091245
sp|Q0VGT2|GLI2_MOUSE Zinc finger protein GLI2 OS=Mus musculus OX=10090 GN=Gli2 PE=1 SV=2//0	-2.0585	7.90E-19	2.04E-16
sp|Q6P5F6|S39AA_MOUSE Zinc transporter ZIP10 OS=Mus musculus OX=10090 GN=Slc39a10 PE=1 SV=1//0	-2.0578	2.14E-07	1.08E-05
sp|Q6PFX6|CAD24_MOUSE Cadherin-24 OS=Mus musculus OX=10090 GN=Cdh24 PE=2 SV=1//0	-2.0491	8.53E-06	0.00028626
sp|Q640L5|CCD18_MOUSE Coiled-coil domain-containing protein 18 OS=Mus musculus OX=10090 GN=Ccdc18 PE=1 SV=1//0	-2.041	7.73E-08	4.37E-06
sp|Q80SV1|LHPL1_MOUSE LHFPL tetraspan subfamily member 1 protein OS=Mus musculus OX=10090 GN=Lhfpl1 PE=2 SV=1//1.01507e-129	-2.0335	7.08E-09	4.99E-07
sp|Q6IFT4|RHG20_MOUSE Rho GTPase-activating protein 20 OS=Mus musculus OX=10090 GN=Arhgap20 PE=1 SV=1//0	-2.0179	0.0018139	0.029416
sp|Q9D7D2|SPA9_MOUSE Serpin A9 OS=Mus musculus OX=10090 GN=Serpina9 PE=2 SV=1//0	-2.0012	7.03E-06	0.00024126
