# Supplementary material for: Fibroblast A20 governs fibrosis susceptibility and its repression by DREAM promotes fibrosis in multiple organs
Source: Nat Commun. 2022 Oct 26;13:6358. doi: 10.1038/s41467-022-33767-y (PMC9606375; doi:10.1038/s41467-022-33767-y)
Supplement: Supplementary file 2 — Reporting Summary [file 41467_2022_33767_MOESM2_ESM.pdf]

nature portfolio

Corresponding Authors: Swati Bhattacharyya and John Varga
*DBPR and Manuscript Number: NCOMMS-21-06732B

Last updated by author(s): Jun 20, 2022

Reporting Summary

Nature Portfolio wishes to improve the reproducibility of the work that we publish. This form provides structure for consistency and transparency in reporting. For further information on Nature Portfolio policies, see our Editorial Policies and the Editorial Policy Checklist.

Please do not complete any field with "not applicable" or n/a. Refer to the help text for what text to use if an item is not relevant to your study.
For final submission: please carefully check your responses for accuracy; you will not be able to make changes later.

Statistics

For all statistical analyses, confirm that the following items are present in the figure legend, table legend, main text, or Methods section.

n/a	Confirmed	
☐	☒	The exact sample size (n) for each experimental group/condition, given as a discrete number and unit of measurement
☐	☒	A statement on whether measurements were taken from distinct samples or whether the same sample was measured repeatedly
☐	☒	The statistical test(s) used AND whether they are one- or two-sided<br>*Only common tests should be described solely by name; describe more complex techniques in the Methods section.*
☒	☐	A description of all covariates tested
☐	☒	A description of any assumptions or corrections, such as tests of normality and adjustment for multiple comparisons
☐	☒	A full description of the statistical parameters including central tendency (e.g. means) or other basic estimates (e.g. regression coefficient) AND variation (e.g. standard deviation) or associated estimates of uncertainty (e.g. confidence intervals)
☐	☒	For null hypothesis testing, the test statistic (e.g. F, t, r) with confidence intervals, effect sizes, degrees of freedom and P value noted<br>*Give P values as exact values whenever suitable.*
☒	☐	For Bayesian analysis, information on the choice of priors and Markov chain Monte Carlo settings
☒	☐	For hierarchical and complex designs, identification of the appropriate level for tests and full reporting of outcomes
☐	☒	Estimates of effect sizes (e.g. Cohen's d, Pearson's r), indicating how they were calculated

Our web collection on statistics for biologists contains articles on many of the points above.

Software and code

Policy information about availability of computer code

Data collection	No software was used for data collection
Data analysis	Image-J2 was used for image analysis. Graph pad prism version 8 was used for statistical data interpretation, DESeq2 R package was used for differential expression analysis between two conditions/groups, R package cluster Profiler to test the statistical enrichment of differential expression genes in KEGG pathways. Cluster 3.0 and TreeView V2 were used to create a heatmap.

For manuscripts utilizing custom algorithms or software that are central to the research but not yet described in published literature, software must be made available to editors and reviewers. We strongly encourage code deposition in a community repository (e.g. GitHub). See the Nature Portfolio guidelines for submitting code & software for further information.

Data

Policy information about availability of data

All manuscripts must include a data availability statement. This statement should provide the following information, where applicable:

- Accession codes, unique identifiers, or web links for publicly available datasets
- A description of any restrictions on data availability
- For clinical datasets or third party data, please ensure that the statement adheres to our policy

All the data will be available upon request. Accession code for RNA sequencing submission is GSE194380. No restriction of data availability except for clinical data. We have used already recruited patient skin and lung biopsies as well as fibroblasts explanted from those biopsies. Each individual is de-identified. We have enclosed those details in Table I of the manuscript that discloses required information for the study. Datasets used in this study include GSE59785[https://www.ncbi.nlm.nih.gov/geo/query/acc.cgi?acc=GSE59785], GSE45485[https://www.ncbi.nlm.nih.gov/geo/query/acc.cgi?acc=GSE45485], GSE32413[https://www.ncbi.nlm.nih.gov/geo/query/acc.cgi?acc=GSE32413], GSE194380[https://www.ncbi.nlm.nih.gov/geo/query/acc.cgi?acc=GSE194380], GSE9285[https://www.ncbi.nlm.nih.gov/geo/query/acc.cgi?acc=GSE9285]

Field-specific reporting

Please select the one below that is the best fit for your research. If you are not sure, read the appropriate sections before making your selection.

- [x] Life sciences
- [ ] Behavioural & social sciences
- [ ] Ecological, evolutionary & environmental sciences

Life sciences study design

All studies must disclose on these points even when the disclosure is negative.

Sample size	Studies using human samples utilized data from publicly available databases (including independent validation cohort when available), as indicated; or available biopsy samples for immunolabelling. For clinical studies (for both skin and lung biopsies and using fibroblasts from healthy and patients), we were not able to use sample size determination because of the lack of available samples. For patient skin biopsy, we used a 2nd cohort of patients to confirm our data. For lung biopsies, we used the number of available tissues. There were no exclusion criteria. For other patient related studies, we used a publicly available transcriptome dataset as mentioned in the manuscript text. For mouse studies, group sizes were determined following our previously published studies sample size determination. In addition, all mouse experiments were independently replicated for two times with no exclusion criteria."
Data exclusions	No data were excluded in any of the experiments.
Replication	Studies using analysis of human data were replicated by analyzing data from independent validation cohort when available, as indicated. Experiments with mice were replicated twice.
Randomization	We randomized the mice for PBS and bleomycin injections. The cells from patient and healthy volunteers were already collected, named, and assigned groups. This meant that we could not randomize the groups.
Blinding	All mouse experimental data were analyzed by observers blinded to group identity. We used de-identified tissue samples from already archived and numbered as healthy and patients' samples as mentioned in the manuscript table 1. However, during analysis, we were blinded to patients' characteristics and subgrouping such as disease severity, early or late stages of the disease.

Reporting for specific materials, systems and methods

We require information from authors about some types of materials, experimental systems and methods used in many studies. Here, indicate whether each material, system or method listed is relevant to your study. If you are not sure if a list item applies to your research, read the appropriate section before selecting a response.

Materials & experimental systems

n/a	Involved in the study	
[ ]	[x]	Antibodies
[ ]	[x]	Eukaryotic cell lines
[x]	[ ]	Palaeontology and archaeology
[ ]	[x]	Animals and other organisms
[ ]	[x]	Human research participants
[x]	[ ]	Clinical data
[x]	[ ]	Dual use research of concern

Methods

n/a	Involved in the study	
[x]	[ ]	ChIP-seq
[x]	[ ]	Flow cytometry
[x]	[ ]	MRI-based neuroimaging

Antibodies

Antibodies used	**A20 (rabbit, Abeam, ab92324); DREAM (Santa Cruz, sc166916); pro-collagen 1 (EMO Millipore, MAB1912, M-58 clone); F4/80 (rabbit, eBioscience, 14-4801-82); aSMA (rabbit, Abeam, ab5694); B-catenin (Abeam, ab32572); FN-EDA (Sigma, F6140); Perilipin (Abcam, ab61682); phospho-TAK (Cell Signaling, 4531S); phospho-FAK (Cell Signaling, 3284T); TNC-C (Abcam, ab108930); human Type I collagen (Southern Biotechnology, 1310-01) Western Blot: A20(Santa Cruz, Sc 166692); aSMA (Sigma, 5228); tubulin (Sigma-Aldrich); 8-actin (Sigma, A5441); GAPDH (Santa Cruz, sc 365062); pSmad-2 (Cell Signaling, 3108S); Ubiquitin/P401 (Santa Cruz, sc8017); aSMA (Sigma); Immunoprecipitation: TRAF6 (Santa Cruz, sc7221); Secondaries: Chicken anti-Rabbit 488 (ThermoFisher, A21441, Lot:1796684); Goat anti-Rabbit 594 (ThermoFisher, A11037, Lot: 1915919); Donkey anti-Goat 594 (ThermoFisher, A11058, Lot:1445994); Donkey anti-Goat 488 (ThermoFisher, A11055, Lot:1687906); Donkey anti-Mouse 594 (ThermoFisher, A21203, Lot:1163390); Chicken anti-Mouse 488 (ThermoFisher, A21200, Lot:1078785); Chicken anti-Rat 594 (ThermoFisher, A21471, Lot:1003225)**
Validation	From the manufacturers' websites, we found that a variety of methods were used for antibody validation including mouse knockout models, dominant negative mutants, morpholinos, siRNA, and most recently, gene editing.

Eukaryotic cell line

Policy information about cel

Cell line source(s)	Wild-type Mouse embryonic fibroblasts (MEFs); A20-/- MEFs; fibroblasts from forearm skin biopsies in SSc patients and healthy controls; fibroblasts from neonatal mouse lung and skin
Authentication	Foreskin fibroblasts are routinely prepared from anonymous discarded foreskins from circumcised young males at Feinberg

Authentication	Hospital Northwestern University. SSc skin fibroblasts and fibroblasts from age-matched healthy control subjects are routinely generated from 3 mm punch biopsies from the forearm. Fibroblast lines are established by standard explant methods and propagated. Fibroblasts are stored in the Northwestern Scleroderma Biorepository. Mouse embryonic fibroblasts (MEFs) were tested for absence of A20 using A20-specific antibody and by RT-PCR. Mouse skin and lung fibroblasts are obtained by standard explant methods from A20 fl mice and from mouse with A20 deleted in fibroblasts, as well as from wild type mice. Fibroblasts were explanted from DREAM-null mice, and tested for absence of DREAM by RT-PCR.
Mycoplasma contamination	All cell lines were tested for mycoplasma contamination
Commonly misidentified lines (See ICLAC register)	Not applicable

Animals and other organisms

Policy information about studies involving animals; ARRIVE guidelines recommended for reporting animal research

Laboratory animals	A20fl/fl mice (Dr. Averil Ma, University of California San Francisco), A20+/- mice (generated in lab), fibroblast-specific A20 deletion (A20fibcko mice, generated in lab), Tskl/+mice (C57BU6 background, The Jackson Laboratory), A20+/-; Tskl/+mice (generated in lab), DREAM-/-mice (Dr. Chinnaswamy Tiruppathi, University of Illinois, Chicago), COL1A2-CRE (The Jackson Laboratory) and wild type mice (C57BL/6 background) . Details provided in authentication section.<br>Sex: Both male and female were used for the study<br>Age: 6-8 weeks old mice were used for the study<br>Animals were allowed free access to food and water ad libitium and maintained with 12/12 h light and dark cycle, with temperatures of 65-75°F and 50-60 % of humidity conditions.
Wild animals	The study did not involve wild animals
Field-collected samples	Not applicable
Ethics oversight	All animal studies were conducted in accordance with NIH guidelines for the care and use of laboratory animals, and under protocols approved by the Northwestern University IACUC.

Note that full information on the approval of the study protocol must also be provided in the manuscript.

Human research participants

Policy information about studies involving human research participants

Population characteristics	We have provided this information in table I of the manuscript.
Recruitment	We used samples from patients that were previously recruited. There were no new patients recruited specifically for this study.
Ethics oversight	IRBs of Northwestern University and the University of Pittsburgh. All patients fulfilled ACR criteria for the classification of SSc

Note that full information on the approval of the study protocol must also be provided in the manuscript.

Clinical data

Policy information about clinical studies

All manuscripts should comply with the ICMJE guidelines for publication of clinical research and a completed CONSORT checklist must be included with all submissions.

Clinical trial registration	Not applicable
Study protocol	Not applicable
Data collection	Not applicable
Outcomes	Not applicable
