# Supplementary material for: Fibroblast A20 governs fibrosis susceptibility and its repression by DREAM promotes fibrosis in multiple organs
Source: Nat Commun. 2022 Oct 26;13:6358. doi: 10.1038/s41467-022-33767-y (PMC9606375; doi:10.1038/s41467-022-33767-y)
Supplement: Supplementary file 4 — Description of Additional Supplementary Files [file 41467_2022_33767_MOESM4_ESM.pdf]

Title: Supplementary Data 1:
Description: Circulating IgG autoantibody production from PBS-treated A20fl/fl vs A20+/- mice. Four mice from A20fl/fl and five mice from A20+/- groups were studied.

Title: Supplementary Data 2:

Description: Supplementary Data 2A: Genome wide transcript changes in the skin from bleomycin and PBS-treated A20fl/fl mice

Three mice from A20fl/fl and A20+/- groups were studied. Genes with >2-fold increase or

decrease and $p < 0.01$; FDR, 0.05 are shown.

Supplementary Data 2B: Genome wide transcript changes in the skin from bleomycin and PBS-treated A20+/- mice

Three mice from A20fl/fl and A20+/- groups were studied. Genes with >2-fold increase or

decrease and $p < 0.01$; FDR, 0.05 are shown.
